# Total syntheses of spiroviolene and spirograterpene A: a structural reassignment with biosynthetic implications†

**DOI:** 10.1039/d0sc04686h

**Published:** 2020-09-30

**Authors:** Hyung Min Chi, Charles J. F. Cole, Pengfei Hu, Cooper A. Taylor, Scott A. Snyder

**Affiliations:** Department of Chemistry, University of Chicago 5735 S. Ellis Avenue Chicago IL 60637 USA sasnyder@uchicago.edu

## Abstract

The recent natural product isolates spiroviolene and spirograterpene A are two relatively non-functionalized linear triquinane terpenes with a large number of structural homologies. Nevertheless, three significant areas of structural disparity exist based on their original assignments, one of which implies a key stereochemical divergence early in their respective biosyntheses. Herein, using two known bicyclic ketone intermediates, a core Pd-catalyzed Heck cyclization sequence, and several chemoselective transformations, we describe concise total syntheses of both natural product targets and propose that the structure of spiroviolene should be reassigned. As a result, these natural products possess greater homology than previously anticipated.

## Abstract 2

Concise syntheses of spiroviolene and spirograterpene A have been achieved from a common intermediate, indicating a structure revision of one is necessary along with implications for its biosynthesis.

## Graphical abstract

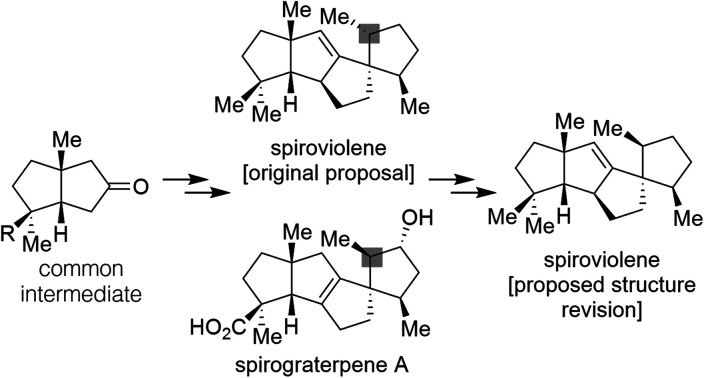

## Introduction

**Scheme 1:**
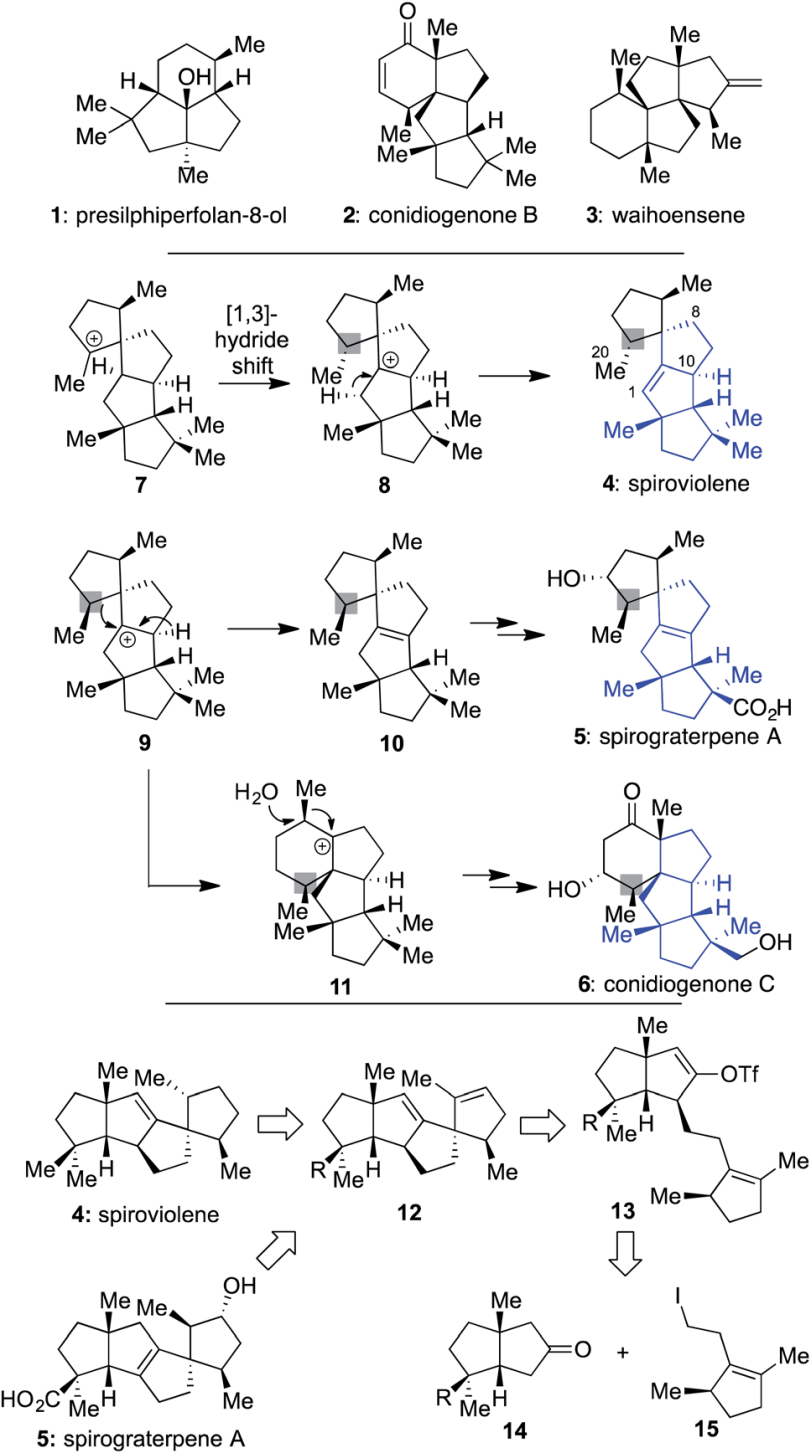
Unique terpene natural products, the conserved linear triquinane core of spiroviolene (**4**), spirograterpene A (**5**), and the conidiogenones (**2** and **6**), and a proposed synthesis of both **4** and **5***via* a common pathway and set of intermediates to clarify the stereochemical identify of the chiral center denoted with a gray box.

In recent years, chemists have devoted much attention to addressing the synthetic challenges posed by terpenes, such as **1–3** (Scheme 1), which possess a modicum of traditionally reactive functional groups.^1–4^ Indeed, efforts with such molecules have suggested that since functional groups are typically required to forge C–C bonds, they should be chosen strategically and used for multiple purposes to limit step counts since they often are target superfluous.^5^ In addition, when multiple quaternary centers are present,^6^ they should be used as an essential part of retrosynthetic planning, focused on the question of how the presence of one might assist in the formation of others.^2*b*^

It was against this backdrop that we became interested in the structures of spiroviolene (**4**)^7^ and spirograterpene A (**5**),^8^ two recently disclosed linear triquinanes characterized largely on the basis of NMR data. The first of these was isolated by Dickschat and co-workers from the treatment of a terpene cyclase analog (termed spiroviolene synthase, SvS) with geranylgeranyl diphosphate (GGPP).^7^ The other was obtained by the Yang group from *Penicillium granulatum* MCCC 3A00475 and was shown to possess modest anti-allergic activity.^8^ From a structural perspective, these molecules possess a high degree of structural homology, with both containing the same core carbon skeleton comprised of 4 rings and 3 all-carbon quaternary centers. However, they also have three main differences: their overall oxidation level, with **5** possessing an additional hydroxyl and carboxylic acid group as opposed to the saturated hydrocarbon units of **4**, the specific placement of their central alkene, and the stereochemical disposition of a methyl group at the highlighted position. It was the latter of these differences that especially caught our attention since its accuracy would require a divergence in how those chiral centers were originally set as part of their respective biosyntheses. Indeed, in the Dickschat work^7^ it was postulated that this key stereocenter might arise from intermediate **7***via* a facially selective 1,3-hydride shift to produce **8**; deuterium-labelling studies supported that stereochemical assessment, assuming that all stereocenters were set as drawn in the initial cyclization of linear GGPP leading to this polycycle.^9^ Subsequent proton elimination would then afford spiroviolene (**4**).^10^ By contrast, as postulated by Yang,^8^ if diastereomer **9** could also be obtained from a cyclization process involving GGPP, then loss of an alternate hydride could afford the distinctive alkene of **10**, ultimately leading to spirograterpene A (**5**) through further oxidations. Herein, we delineate a unified approach capable of achieving the expeditious total synthesis of both natural products. As a result, we also provide a body of evidence implicating that the structure of spiroviolene is in error and has a stereochemical disposition of its highlighted chiral center which matches that of spirograterpene A.

## Results and discussion

Given that we had already synthesized several members of the conidiogenones as single enantiomers and verified their absolute configuration in the process, including **2** and **6**,^2*b*^ we surmised that the stereochemical assignments for spirograterpene A (**5**) were likely accurate given that it was co-isolated with **6** and other members of the class. Biosynthetically, what was required to obtain these conidiogenone architectures from **9** was a 1,2-alkyl shift, instead of a β-hydride elimination, to afford reactive intermediate **11**. That material, following a further 1,2-alkyl shift and attack by water, could afford most of the structural elements of **6**.^8^ As such, to determine whether or not **4** and **5** were accurately assigned, we developed the retrosynthetic approach shown in the bottom of Scheme 1; the structures of **4** and **5** have been rotated here from their initial presentation. Our hope was that intermediates of type **12**, differing solely in terms of their R group, could afford the means to access both targets and address any stereochemical ambiguities. In that regard, we assumed that the central alkene within **12** could be isomerized to that of its tetrasubstituted variant in spirograterpene A under thermodynamic control, and that the distal alkene could be chemoselectively functionalized through hydrogenation or hydroboration, respectively, to afford the final patterning of both natural products. We hoped that the latter of these functionalizations could be effected without complete facial control to provide access to both diastereomers to aid in structural assignments. In turn, the key quaternary spirocyclic stereocenter within **12** was anticipated to arise *via* a Heck cyclization^11^ from vinyl triflate **13**, itself prepared from the regiospecific merger of bicyclic ketone **14** and alkyl iodide **15**. These final steps would mirror operations used in our syntheses of both **1** and **2**,^1,2*b*^ commencing from an intermediate set (**14**) already in hand and used as part of our conidiogenone syntheses;^2*b*^ as such, these bicyclic ketones could be envisioned as common intermediates leading to several different classes of natural products if successfully advanced to **4** and **5**.

**Scheme 2:**
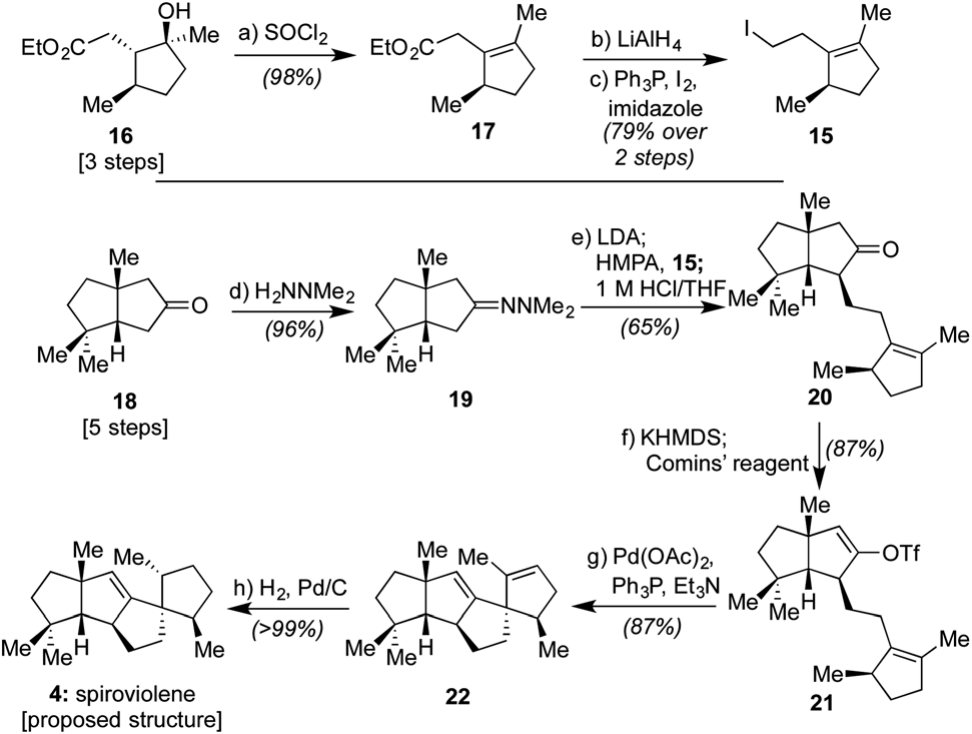
Total synthesis of spiroviolene (**4**) starting from known intermediate **18**: (a) pyridine (1.2 equiv.), CH_2_Cl_2_, then SOCl_2_ (1.1 equiv.), 0 °C to 40 °C, 12 h, 98%; (b) LiAlH_4_ (2.0 equiv.), Et_2_O, 0 °C, then **17**, Et_2_O, 0 °C to 23 °C, 30 min; (c) Ph_3_P (1.2 equiv.), imidazole (1.5 equiv.), CH_2_Cl_2_, 23 °C, 5 min, then I_2_ (1.2 equiv.), 0 °C to 23 °C, 1 h, 79% over 2 steps; (d) H_2_NNMe_2_ (5.0 equiv.), 23 °C, 12 h, 96%; (e) LDA (1.3 equiv.), THF, −78 °C, 15 min, then **18**, THF, 0 °C, 2 h, then HMPA (1.3 equiv.), −78 °C, 10 min, then **15** (1.2 equiv.), THF, −78 °C to 23 °C, 15 h, then 1 M HCl/THF (1 : 1), 23 °C, 12 h, 65%; (f) KHMDS (1.3 equiv.), THF, −78 °C to 0 °C, 2 h, then Comins' reagent (1.1 equiv.), THF, −78 °C, 1 h, 87%; (g) Pd(OAc)_2_ (10 mol%), Ph_3_P (20 mol%), toluene, 23 °C, then Et_3_N (2.0 equiv.), 90 °C, 20 h, 88%; (h) H_2_ (balloon pressure), Pd/C (10 wt%), EtOH, 23 °C, 30 min, >99%.

As shown in Scheme 2, our efforts commenced with the preparation of enantioenriched **15**. Using a procedure developed by Piva and co-workers,^12^ we were able to access γ-hydroxy ester **16** in 3 steps and in ∼90% ee. From here, subsequent regioselective elimination, as promoted by SOCl_2_, led to β,γ-unsaturated ester **17** in near quantitative yield. The desired alkyl iodide was then completed by reduction of the ester to a primary alcohol using LiAlH_4_ followed by an Appel reaction; these operations afforded **15** in 79% overall yield.

Next, following the conversion of known bicyclic ketone **18** to its corresponding hydrazone derivative (**19**), a subsequent regiospecific alkylation with **15** was achieved through the action of LDA and HMPA, using an acidic treatment (1 M HCl/THF) to cleave the hydrazone and afford ketone **20** in 65% isolated yield. In this event, the quaternary center uniting the two five-membered rings is presumed to afford sufficient steric bulk to drive its regioselectivity.^2*b*^ From here, treatment with KHMDS and Comins' reagent led to the regiospecific formation of vinyl triflate **21**, setting the stage for a stereospecific Heck cyclization leading to the requisite 1,3-*trans* stereochemical arrangement of substituents in tetracycle **22**.^11^ These operations proceeded in 87% overall yield. At this point, all that remained was controlled reduction of one of its two alkenes to reach the putative structure of spiroviolene (*i.e.***4**). Given that exploration of molecular models suggested standard hydrogenation might afford the undesired diastereomer preferentially, initial attempts focused on hydrogen atom transfer (HAT)-processes in a radical-type manifold.^13^ However, all efforts under such a regime failed. As such, an attempt was made under more standard conditions using a H_2_ atmosphere with Pd/C in EtOH. To our surprise, this event led to the smooth formation of a single product in near quantitative yield (98%). Its spectral properties (^1^H and ^13^C NMR) and optical rotation fully matched those reported by Dickschat and co-workers.^7^ Unclear, however, was the identity of the new chiral center within this synthetic material as the face of addition could not be confirmed.

**Scheme 3:**
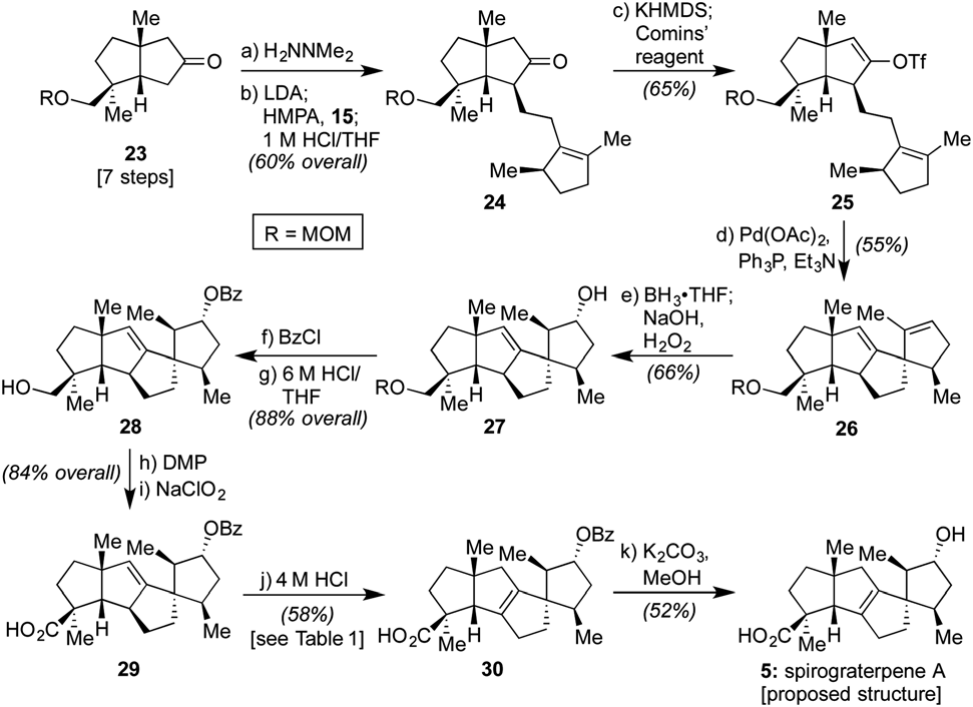
Total synthesis of spirograterpene A (**5**) starting from known intermediate **23**: (a) H_2_NNMe_2_ (3.0 equiv.), 23 °C, 12 h, 96%; (b) LDA (1.5 equiv.), THF, −78 °C, 15 min, then **23**, THF, 0 °C, 2 h, then HMPA (1.5 equiv.), −78 °C, 30 min, then **15** (1.2 equiv.), THF, −78 °C to 23 °C, 15 h, then 1 M HCl, 23 °C, 12 h, 63%; (c) KHMDS (1.3 equiv.), THF, −78 °C to 0 °C, 2 h, then Comins' reagent (1.1 equiv.), THF, −78 °C, 1 h, 65%; (d) Pd(OAc)_2_ (20 mol%), Ph_3_P (40 mol%), Et_3_N (3.0 equiv.), toluene, 23 °C, then **25**, toluene, 23 °C to 90 °C, 16 h, 55%; (e) BH_3_·THF (1.0 equiv.), THF, 0 °C, 5 h, then NaOH/H_2_O_2_ (1 : 1), 23 °C, 1 h, 66%; (f) BzCl (3.0 equiv.), Et_3_N (4.0 equiv.), 4-DMAP (1.0 equiv.), CH_2_Cl_2_, 23 °C, 2 h, 96%; (g) 6 M HCl/THF (1 : 2), 50 °C, 4 h, 92%; (h) Dess–Martin periodinane (1.5 equiv.), NaHCO_3_ (5.0 equiv.), CH_2_Cl_2_, 23 °C, 1 h, 85%; (i) 2-methyl-2-butene (10 equiv.), *t*-BuOH, then NaClO_2_ (35 equiv.), NaH_2_PO_4_ (50 equiv.), H_2_O, 23 °C, 1 h, 96%; (j) HCl (4 M in 1,4-dioxane), 80 °C, 48 h, 58%; (k) K_2_CO_3_ (3.0 equiv.), MeOH, 55 °C, 15 h, 52%.

As such, we endeavored next to synthesize spirograterpene A (**5**, *cf.*Scheme 1). As indicated in Scheme 3, the initial elements of our route were effected following the same general sequence as for spiroviolene, starting with the MOM-protected **23**.^2*b*^ Alternate modes of protection provided distinct challenges in appending alkyl iodide **15**, but with this group in place, that event, as well as subsequent vinyl triflate formation and Heck cyclization, afforded smooth access to tetracycle **26**. At this juncture, the remaining major operations consisted of olefin isomerization and a series of oxidative manipulations, events that, in principle, could be conducted in either order. In practice, however, we were unable to effect olefin migration to its arguably more stable tetrasubstituted isomer at this stage as a series of undesired rearrangements were observed under a variety of conditions. As such, we exposed **26** to a standard hydroboration/oxidation protocol. This event afforded a single product in 66% yield, drawn here as **27** to match the stereochemistry proposed for spirograterpene A, though again of unknown configuration at this stage (*vide infra*).

With this material in hand, efforts were once again made to effect olefin isomerization, but either no transformation or decomposition was observed. As a result, we protected the secondary alcohol as a benzoate ester and adjusted the oxidation state of the other alcohol motif to that of a carboxylic acid over three additional steps to deliver **29**.^14,15^ As shown in Table 1, the alkene within this material proved equally challenging to migrate, with a number of transition metals and photochemical conditions failing to afford any desired product.^16^ Under acidic conditions, including treatment with *p*-TsOH or TfOH, we observed the recovery of starting material, even at elevated temperatures. However, when 4 M HCl in 1,4-dioxane was used at 55 °C over the course of 12 h, 15% conversion to the desired product was detected by ^1^H NMR analysis based on the presence of a diagnostic allylic proton peak. By increasing the temperature (80 °C) and prolonging the reaction time (72 h), the desired alkene could be obtained in 58% isolated yield.^17^ Finally, methanolysis of the benzoate ester with K_2_CO_3_ at 55 °C afforded material which matched both the spectral (^1^H and ^13^C NMR) and optical rotation data obtained by the Yang group for spirograterpene A (**5**).^8^

**Table 1:** Conditions screened to effect the critical olefin isomerization

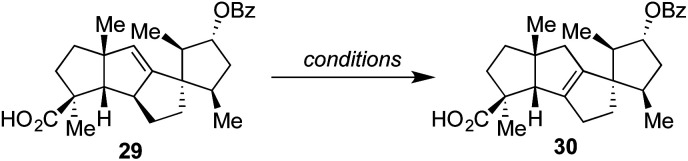		
Entry	Conditions	Result
1	[Pd(CH_3_CN)_4_](BF_4_)_2_, CH_3_CN, 23 °C, 12 h	No reaction
2	[Pd(CH_3_CN)_4_](BF_4_)_2_, CH_3_CN, 80 °C, 12 h	No reaction
3	UV (<365 nm), Et_2_O, 23 °C, 1 h	No reaction
4	UV (<365 nm), Et_2_O, 23 °C, 12 h	Decomposition
5	*p*-TsOH, toluene, 100 °C, 12 h	No reaction
6	*p*-TsOH, 1,4-dioxane, 100 °C, 12 h	No reaction
7	TfOH, 1,4-dioxane, 100 °C, 12 h	No reaction
8	HCl (4 M in 1,4-dioxane), 23 °C, 12 h	No reaction
9	HCl (4 M in 1,4-dioxane), 55 °C, 12 h	<15% conversion
10	HCl (4 M in 1,4-dioxane), 80 °C, 72 h	Full conversion (58% yield)

With both targets in hand, and both events proceeding with a fully facially selective alkene functionalization (*i.e.* hydrogenation and hydroboration, respectively) on intermediates differing solely by the presence of a remote MOM-protected alcohol in the case of **26** (*cf.*Scheme 3), our conclusion was that the one of the two natural products was misassigned and that the configuration about their highlighted centers as denoted in Scheme 1 is conserved. Unfortunately, despite multiple efforts encompassing a range of synthetic derivatives, we were unable to obtain suitable material for X-ray diffraction to aid in such a reassignment.^18^ As such, computational, chemical, and spectroscopic means were explored instead.

**Scheme 4:**
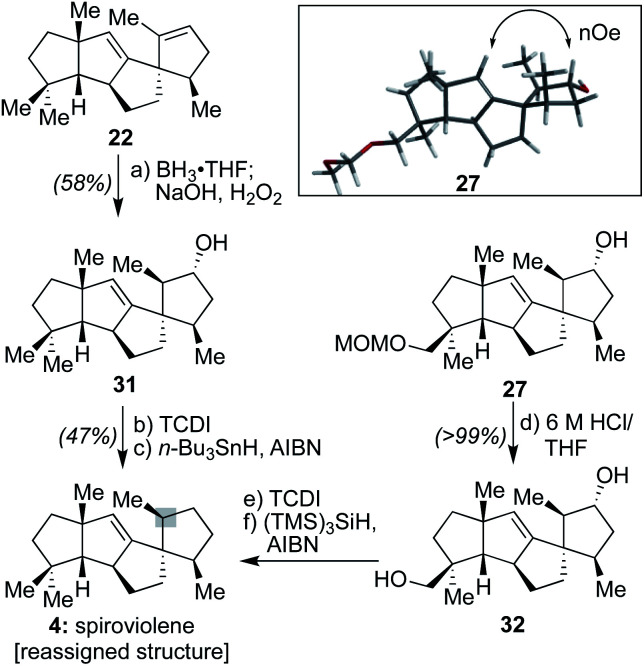
Synthetic and NMR support for the structural reassignment of spiroviolene (**4**): (a) BH_3_·THF (1.0 equiv.), THF, 0 °C, 5 h, then NaOH/H_2_O_2_ (1 : 1), 23 °C, 1 h, 58%; (b) TCDI (3.0 equiv.), 4-DMAP (0.5 equiv.), CH_2_Cl_2_/pyridine (1 : 1), 23 °C, 12 h; (c) *n*-Bu_3_SnH (2.0 equiv.), AIBN (0.15 equiv.), toluene, 110 °C, 10 min, 47%; (d) 6 M HCl/THF (1 : 2), 50 °C, 4 h, >99%; (e) TCDI (4.0 equiv.), 4-DMAP (0.5 equiv.), CH_2_Cl_2_/pyridine (1 : 1), 23 °C, 12 h; (f) (TMS)_3_SiH (4.0 equiv.), AIBN (0.2 equiv.), toluene, 110 °C, 30 min.

First, DFT transition state analysis for the hydroboration of **26** (see ESI†) revealed that in the potential energy surface for the transformation, there is a difference of 8.0 kcal mol^−1^ favoring hydroboration from the α-face, thereby supporting the stereochemical outcome originally proposed for spirograterpene A. In addition, NOE experiments with **27** (Scheme 4) revealed an interaction between the vinylic proton and the proton geminal to the hydroxyl group, one that would be possible only had the hydroboration taken place from the indicated, and computationally expected, face. These results are in agreement with the Mosher's ester analysis performed by the isolation team, which indicated that the absolute stereochemistry of the secondary alcohol is as drawn.^8^

Further, as shown in Scheme 4, to assess whether the MOM-protecting group of **26** played any role in leading to a differential facial preference for functionalization, alkene **22** was subjected to the same hydroboration/oxidation sequence to afford **31**. Subsequent treatment with 1,1′-thiocarbonyldiimidazole (TCDI) and subjection of the resultant crude thiocarbamate intermediate to typical Barton–McCombie deoxygenation conditions^19^ afforded a sample with spectral properties that matched that of spiroviolene. Finally, intermediate **27**, following conversion to diol **32** and a similar double Barton–McCombie deoxygenation, also afforded material with spectral properties that matched spiroviolene.^20^

Finally, we also considered the collated body of spectral data for natural spiroviolene collected by the Dickschat group. In particular, we examined key NOE correlations that served as a core element for the initial structural assignment. Specifically, the observed interaction between H-10 and C-20 (see Scheme 1 for positional numbering) as well as the interaction between H-8α and C-20, would appear to support both the original and revised structures. However, there is an additional interaction, albeit relatively weak, between the H-1 vinyl proton and C-20, one that could only be possible with the relative stereochemistry displayed in the revised structure.

As such, based on this collated evidence, we believe that the structure of spiroviolene should be reassigned to that of structure **4** as drawn within Scheme 4, with stereochemistry at the key center matching that of the originally assigned spirograterpene A. This result would thus suggest that **9** (*cf.*Scheme 1), not **8**, is a likely biosynthetic precursor for **4**, with implications for the stereochemical outcome of cyclizations and/or other events starting from GGPP to account for its formation. At what point in that biosynthesis this alteration would occur is unclear, and it may be distinct for the route used to reach **9** for spirograterpene A given that these natural products are produced by bacteria and fungi, respectively.

## Conclusions

In conclusion, we have achieved concise and enantioselective syntheses of both spiroviolene and spirograterpene A, in 10 and 18 steps, respectively, utilizing previously prepared bicyclic ketone building blocks coupled with a powerful Heck cyclization sequence and other chemoselective transformations. As a result of computational, synthetic, and spectroscopic investigations, we believe that a previously assigned chiral center within spiroviolene is in error, with the two natural products likely sharing an overall biosynthetic pathway despite being produced in different organisms. Efforts to extend several of the findings of this work, including toward the synthesis of other non-functionalized terpenes, are the subject of current endeavors.

## Author contributions

S. A. S., H. M. C., and P. H. conceived the project. S. A. S. directed the research, and all authors composed the manuscript and the ESI section.† H. M. C. and C. J. F. C. executed the total syntheses and C. A. T. performed the calculations. P. H. provided intellectual contribution for route design.

## Conflicts of interest

There are no conflicts to declare.

## Supplementary Material

SC-011-D0SC04686H-s001
