# Supplementary material for: Total syntheses of spiroviolene and spirograterpene A: a structural reassignment with biosynthetic implications
Source: Chem Sci. 2020 Sep 30;11(40):10939–44. doi: 10.1039/d0sc04686h (PMC8162393; doi:10.1039/d0sc04686h)
Supplement: SC-011-D0SC04686H-s001 [file SC-011-D0SC04686H-s001.pdf]

Electronic Supplementary Material (ESI) for Chemical Science.
This journal is © The Royal Society of Chemistry 2020

Total Syntheses of Spiroviolene and Spirograterpene A: A Structural Reassignment with Biosynthetic Implications

Hyung Min Chi, Charles J. F. Cole, Pengfei Hu, Cooper A. Taylor, Scott A. Snyder*

Department of Chemistry, The University of Chicago, 5735 S. Ellis Avenue, Chicago, IL 60637 (USA)

Supporting Information Section

Table of Contents

A. General Procedures…S2
B. Abbreviations…S2
C. Experimental Procedures and Characterization Data…S3–S21
Alkyl Iodide Side Chain **15**…S3–S4
Mosher Ester **S2**…S4
Total Synthesis of Spiroviolene (**4**)
Bicyclic Ketone **20**…S4–S5
Triflate **21**…S6
Diene **22**…S6–S7
Spiroviolene **4**…S7
Table S1…S8–S10
Total Synthesis of Spirograterpene A (**5**)
Bicyclic Ketone **24**…S10–S11
Diene **26**…S11–S12
Secondary Alcohol **27**…S12
Benzoate **S5**…S12–S13
Primary Alcohol **28**…S13
Carboxylic Acid **29**…S14
Tetrasubstituted Alkene **30**…S15
Spirograterpene A **5**…S15
Table S2…S16–S17
Alcohol **31**…S18
Barton Deoxygenation Product **4**…S18–S19
Diol **32**…S20
Double Barton Deoxygenation Product **4**…S20–S21
D. Computational General Information…S22
E. Potential Energy Surface and Minimized Structures…S22–S36
F. References…S37
G. NMR Spectra…S38–S72
H. HPLC Traces…S73

Experimental Data for Compounds

A. General Procedures. All reactions were carried out under an argon atmosphere with dry solvents under anhydrous conditions, unless otherwise noted. Dry tetrahydrofuran (THF), toluene, diethyl ether (Et_2O), dichloromethane (CH_2Cl_2), and acetonitrile (CH_3CN) were obtained by passing commercially available pre-dried, oxygen-free formulations through activated alumina columns. Yields refer to chromatographically and spectroscopically (^1H and ^{13}C NMR) homogeneous materials, unless otherwise stated. Reagents were purchased at the highest commercial quality and used without further purification, unless otherwise stated. Reactions were magnetically stirred and monitored by thin-layer chromatography (TLC) carried out on 0.25 mm E. Merck silica gel plates (60F-254) using UV light as visualizing agent, and an ethanolic solution of phosphomolybdic acid and cerium sulfate or a solution of $KMnO_4$ in aq. $NaHCO_3$ and heat as developing agents. SiliCycle silica gel (60, academic grade, particle size 0.040–0.063 mm) was used for flash column chromatography. Preparative thin-layer chromatography separations were carried out on 0.50 mm E. Merck silica gel plates (60F-254). NMR spectra were recorded on Bruker 400 and 500 MHz instruments and calibrated using residual undeuterated solvent as an internal reference. The following abbreviations were used to explain the multiplicities: s = singlet, d = doublet, t = triplet, q = quartet, br = broad, app = apparent. IR spectra were recorded on a Perkin-Elmer 1000 series FT-IR spectrometer. High-resolution mass spectra (HRMS) were recorded on Agilent 6244 Tof-MS using ESI (Electrospray Ionization) at the University of Chicago Mass Spectroscopy Core Facility. All *ee* values were determined by HPLC on Daicel Chiralcel or Chiralpak columns.

B. Abbreviations. THF = tetrahydrofuran, Et_2O = diethyl ether, EtOAc = ethyl acetate, 4-DMAP = 4-dimethylaminopyridine, Et_3N = triethylamine, *i*-PrOH = isopropanol, *t*-BuOH = *tert*-butanol, AIBN = 2,2’-azobis(2-methyl propionitrile), DCE = 1,2-dichloroethane, TMSOTf = trimethylsilyl trifluoromethanesulfonate, LDA = lithium diisopropyl amide, KHMDS = potassium hexamethyldisilazane, HMPA = hexamethylphosphoramide, Comins’ Reagent = *N*-(5-chloro-2-pyridyl)bis(trifluoromethanesulfonimide), $Pd(OAc)_2$ = palladium acetate, DMP = Dess–Martin Periodinane, MOM = methoxymethyl ether.

C. Experimental Procedures and Characterization Data

Synthesis of Alkyl Iodide Side Chain 15

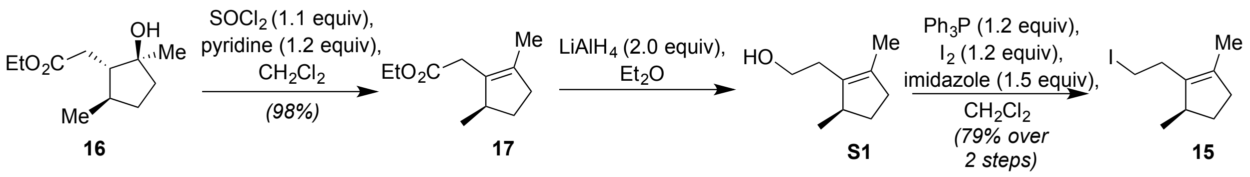

[Note: Compound **16** was prepared according to the procedure reported by Piva *et al.*[1] and all spectral properties matched published values.]

Alkyl Iodide 15. To a flame-dried, 250 mL flask was added **16** (1.67 g, 8.35 mmol, 1.0 equiv), pyridine (0.805 mL, 10.0 mmol, 1.2 equiv) and CH_2Cl_2 (40 mL). A reflux condenser was attached and the system was purged with Ar. The reaction mixture was cooled to 0 °C and $SOCl_2$ (0.670 mL, 9.19 mmol, 1.1 equiv) was added dropwise. The contents of the flask were then immersed into a preheated oil bath at 40 °C and heated at that temperature for 12 h. Upon completion, the mixture was poured over crushed ice and further diluted with CH_2Cl_2 (20 mL). The layers were separated and the aqueous layer was extracted with CH_2Cl_2 (3 × 20 mL). The organic layers were combined, dried (Na_2SO_4) and concentrated *in vacuo* at 5 °C, to give a brown oil. This crude product was further purified by flash column chromatography (silica gel, pentanes/Et_2O, 9/1) to give the desired volatile alkene **17** (1.50 g, 98% yield) as a pale brown oil. Pressing forward without any additional purification, to a flame-dried, 250 mL flask was added $LiAlH_4$ (0.63 g, 16.7 mmol, 2.0 equiv) and Et_2O (60 mL) and the suspension was cooled to 0 °C. A solution of **17** (1.50 g, 8.23 mmol, 1.0 equiv) in Et_2O (20 mL) was then added dropwise. The reaction contents were then warmed to 23 °C and stirred for 30 min. Upon completion, the reaction was quenched by the careful addition of H_2O (0.5 mL), followed by NaOH (1.0 mL, 2 N aqueous) and H_2O (1.5 mL). The resultant slurry was stirred for 30 min at 23 °C after which it was filtered through Celite, washing the pad with Et_2O (3 × 50 mL). The filtrate was then concentrated *in vacuo* at 5 °C and the crude alcohol **S1** (1.36 g) was taken to the next step without any further purification. Thus, next, to a flame-dried, 25 mL flask was added Ph_3P (0.472 g, 1.80 mmol, 1.2 equiv), imidazole (0.153 g, 2.25 mmol, 1.5 equiv), a portion of the crude alcohol **S1** (0.210 g, 1.50 mmol, 1.0 equiv) and CH_2Cl_2 (3 mL). This mixture was stirred for 5 min at 23 °C and then cooled to 0 °C at which time I_2 (0.457 g, 1.80 mmol, 1.2 equiv) was added slowly, portionwise. The mixture was then warmed to 23 °C and stirred for 1 h. Upon completion, the reaction was quenched by the addition of pentanes (5 mL) and stirred at 23 °C for 1 h. The contents of the flask were then filtered through a plug of silica gel eluting using pentanes and the eluted material was concentrated *in vacuo* at 5 °C to give an orange oil. This crude material was further purified by flash column chromatography (silica gel, pentanes/Et_2O, 20/1) to give alkyl iodide **15** (0.298 g, 79% yield) as a colorless oil. **15**: R_f = 0.81 (silica gel, hexanes/EtOAc, 4/1); $[\alpha]_D^{20}$ = +2.4° (*c* = 1.0, $CHCl_3$); IR (film) ν_{max} 2952, 2925, 2864, 2839, 1454, 1435, 1165, 742, 696 cm^{-1}; ^1H NMR (500 MHz, $CDCl_3$) δ 3.19 (td, *J* = 9.5, 5.0 Hz, 1 H),

3.05 (td, J = 9.4, 7.5 Hz, 1 H), 2.68 (ddd, J = 13.9, 9.6, 7.5 Hz, 2 H), 2.62–2.53 (m, 1 H), 2.28–2.12 (m, 2 H), 2.08–2.00 (m, 1 H), 1.62 (dd, J = 2.0, 1.1 Hz, 3 H), 1.31 (ddt, J = 12.7, 9.0, 6.4 Hz, 1 H), 0.98 (d, J = 6.9 Hz, 3 H); ^{13}C NMR (126 MHz, $CDCl_3$) δ 138.25, 134.72, 41.14, 36.80, 31.34, 31.27, 19.71, 14.43, 4.56.

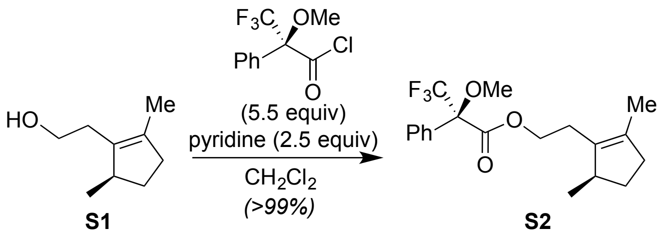

Mosher Ester S2. To a 4 mL vial was added **S1** (1.0 mg, 0.0071 mmol, 1.0 equiv), pyridine (0.0015 mL, 0.018 mmol, 2.5 equiv) and CH_2Cl_2 (0.5 mL). Mosher's acid chloride (0.0074 mL, 0.04 mmol, 5.5 equiv) was added at 23 °C. The reaction mixture was stirred for 6 h after which the reaction was quenched by the addition of saturated aqueous $NaHCO_3$ (1.5 mL) and Et_2O (2 mL). The mixture was vigorously stirred for 30 min to hydrolyze any remaining acid chloride. The organic layer was separated and the aqueous layer extracted with Et_2O (2 × 2 mL). The combined organic layers were combined, dried (Na_2SO_4), and concentrated to give the crude product. This crude material was further purified by flash column chromatography (silica gel, hexanes/EtOAc, 4/1) to give Mosher ester **S2** (2.5 mg, quant.) as a colorless oil. **S2**: R_f = 0.65 (silica gel, hexanes/EtOAc, 4/1); ^{1}H NMR (500 MHz, $CDCl_3$) δ 7.51 (dd, J = 7.2, 2.6 Hz, 2 H), 7.40 (dt, J = 5.3, 2.4 Hz, 3 H), 4.32–4.25 (m, 2 H), 3.57–3.52 (m, 3 H), 2.63 (d, J = 9.1 Hz, 1 H), 2.57 (dt, J = 15.1, 8.1 Hz, 1 H), 2.35–2.19 (m, 3 H), 2.11 (dt, J = 15.1, 7.2 Hz, 1 H), 1.99 (dtd, J = 13.4, 8.5, 4.9 Hz, 1 H), 1.56 (s, 3 H), 0.98 (dd, J = 6.7, 1.5 Hz, 3 H).

Total Synthesis of Spiroviolene (4)

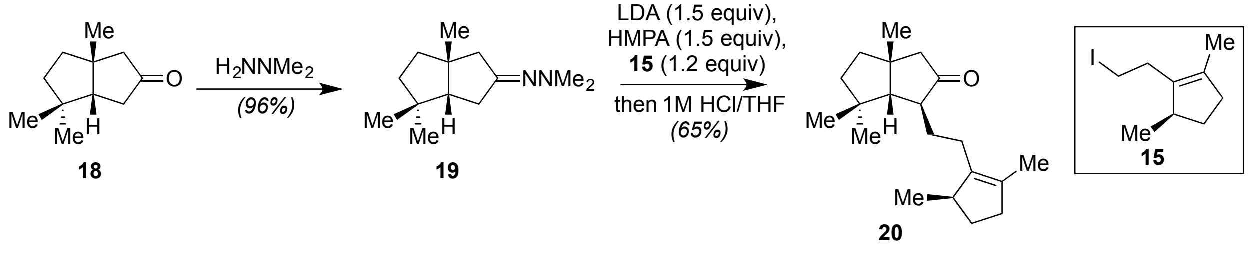

Bicyclic Ketone 20. To a flask containing **18**[2] (0.166 g, 1.00 mmol, 1.0 equiv) was added *N,N*-dimethyl hydrazine (0.380 mL, 5.00 mmol, 5.0 equiv) and the resultant mixture stirred at 23 °C for 12 h. Upon completion the reaction was quenched with NH_4Cl (2 mL) and extracted with Et_2O (3 × 2 mL). The combined organic layers were then washed with H_2O (1 mL), brine (1 mL), dried (Na_2SO_4), and concentrated to give the desired hydrazone **19** (0.200 g, 96% yield) as a yellow oil. To a flame-dried 30-mL Schlenk flask was added solid LDA (0.070 g, 0.65 mmol, 1.3 equiv, used to exclude any incorporation of hexanes) in a glove box at 23 °C. The flask was

capped with a septum and transferred from the glove box to a Schlenk manifold and place under positive pressure of N_2. The flask was cooled to −78 °C and THF (2.5 mL) was added. Once all solids had dissolved, a solution of a portion of hydrazone **19** (0.104 g, 0.50 mmol, 1.0 equiv) in THF (2.5 mL) was added dropwise over the course of 5 min, and the resulting pale yellow solution was stirred at 0 °C for 2 h. The reaction mixture was then recooled to −78 °C and HMPA (0.113 mL, 0.65 mmol, 1.3 equiv) was added dropwise. After stirring for 10 min, a solution of alkyl iodide **15** (0.150 g, 0.60 mmol, 1.2 equiv) in THF (0.6 mL) was added at −78 °C slowly over the course of 5 min. The yellow solution was stirred at –78 °C for 15 h, where temperature gradually warmed to room temperature. The reaction mixture was quenched with saturated aqueous NH_4Cl (5 mL) and extracted with Et_2O (3 × 5 mL). The combined organic layers were dried (Na_2SO_4), filtered, and concentrated to afford the crude product as a pale yellow oil. The crude oil was then further purified by flash column chromatography (silica gel, hexanes/EtOAc, 4/1) to give alkylated hydrazone **S3** (0.118 g, 71% yield) as a pale yellow oil. **S3**: R_f = 0.36 (hexanes/EtOAc, 4/1). [Note: First, solid LDA is necessary for the success of this alkylation. Hexanes from standard commercial *n*-BuLi solutions used for the preparation of LDA was found to be detrimental to the overall conversion and yield. Such efforts included using commercial 1.6 M or 2.5 M *n*-BuLi in THF and gave low conversion (< 30%); by contrast, using 11 M *n*-BuLi in THF gave a similar yield as using solid LDA. For safety concerns, we suggest using solid LDA. Second, hydrazone **19** needs to be used immediately after preparation; much lower conversion was observed when using material that had been stored in a refrigerator at 0 °C for 3 days. Third, both the reaction and the acidic workup are suggested to be performed under a N_2 or Ar atmosphere]. Pressing forward, to a 25-mL round bottomed flask was added alkylated hydrazone **S3** (0.118 g, 0.36 mmol, 1.0 equiv), THF (5 mL), and 1 M aqueous HCl (5 mL) at 23 °C. The reaction mixture was then vigorously stirred at 23 °C for 12 h. Upon completion, the resulting mixture was extracted with Et_2O (5 × 10 mL), and the combined organic layers were dried (Na_2SO_4), filtered, and concentrated to afford a yellow oil. The crude oil was purified by filtration through a short silica gel plug (hexanes/EtOAc, 4/1) to afford alkylated ketone **20** (0.094 g, 92% yield) as a pale yellow oil. **20**: R_f = 0.69 (silica gel, hexanes/EtOAc, 4/1); $[\alpha]_D^{20}$ = –2.3° (*c* = 1.0, $CHCl_3$); IR (film) ν_{max} 2950, 2864, 1738, 1457, 1410, 1385, 1376, 1367, 1351, 1333, 1261, 1169, 1092, 1018, 995, 805 cm^{-1}; ^1H NMR (500 MHz, $CDCl_3$) δ 2.68–2.59 (m, 1 H), 2.31 (d, *J* = 18.1 Hz, 1 H), 2.18–2.10 (m, 4 H), 2.03–1.90 (m, 2 H), 1.81–1.63 (m, 3 H), 1.61 (s, 3 H),1.59–1.52 (m, 4 H), 1.33–1.20 (m, 2 H), 1.19 (s, 3 H), 1.08 (s, 3 H), 0.97 (d, *J* = 6.9 Hz, 3 H), 0.89 (s, 3 H); ^{13}C NMR (125 MHz, $CDCl_3$) δ 138.98, 131.92, 63.96, 53.51, 50.64, 44.93, 42.31, 41.62, 41.36, 40.13, 36.73, 31.65, 31.42, 31.24, 30.56, 29.85, 24.86, 24.53, 19.78, 14.22. [Note: Despite efforts to obtain HRMS data no successful ionization was achieved with either ESI or CI].

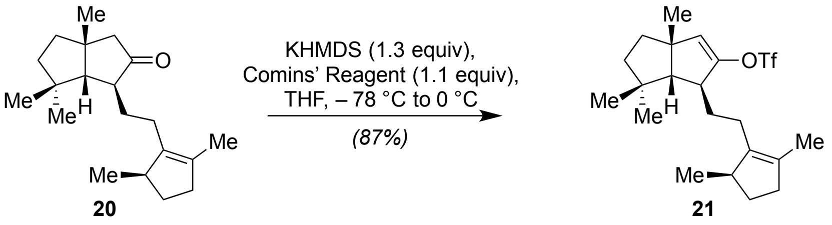

Triflate 21. To a flame-dried 10-mL Schlenk flask was added a solution of ketone **20** (57.7 mg, 0.20 mmol, 1.0 equiv) in THF (2 mL). The resulting yellow solution was cooled to −78 °C and a solution of KHMDS (0.260 mL, 1.0 M in THF, 0.26 mmol, 1.3 equiv) was added. The reaction mixture was stirred at 0 °C for 2 h. Subsequently, the reaction mixture was cooled down to −78 °C) and a solution of Comins' reagent (86.4 mg, 0.22 mmol, 1.1 equiv) in THF (0.37 mL) was added dropwise via syringe. After stirring for 1 h at –78 °C, the yellow solution was diluted with hexanes (5 mL). This mixture was subjected directly to purification by flash column chromatography (silica gel, hexanes/Et_3N, 99/1) to give triflate **21** (73.2 mg, 87% yield) as a colorless oil. **21**: R_f = 0.86 (silica gel, hexanes/EtOAc, 4/1), 0.31 (silica gel, hexanes); $[\alpha]_D^{20}$ = –8.6° (*c* = 1.0, $CHCl_3$); IR (film) ν_{max} 2952, 2864, 1655, 1457, 1423, 1249, 1211, 1144, 1054, 915, 873 cm^{-1}; ^1H NMR (500 MHz, $CDCl_3$) δ 5.42 (d, *J* = 1.1 Hz, 1 H), 2.68–2.60 (m, 2 H), 2.29–2.21 (m, 1 H), 2.19–2.11 (m, 2 H), 2.01 (ddd, *J* = 13.0, 8.3, 4.2 Hz, 1 H), 1.87 (t, *J* = 12.7 Hz, 1 H), 1.78 (ddt, *J* = 14.6, 10.3, 5.0 Hz, 1 H), 1.69–1.63 (m, 2 H), 1.62 (s, 3 H), 1.54 (d, *J* = 2.4 Hz, 1 H), 1.45 (td, *J* = 6.9, 3.9 Hz, 2 H), 1.30 (ddd, *J* = 12.5, 6.2, 2.8 Hz, 1 H), 1.25 (s, 3 H), 1.20–1.12 (m, 1 H), 1.05 (s, 3 H), 0.98 (s, 3 H), 0.96 (s, 3 H); ^{13}C NMR (125 MHz, $CDCl_3$) δ 149.19, 138.85, 131.65, 124.98, 118.7 (q, *J* = 320.7 Hz), 63.49, 52.62, 45.83, 42.37, 41.88, 40.33, 37.42, 36.75, 32.93, 31.43, 30.09, 29.92, 24.56, 24.26, 19.78, 14.12; HRMS (ESI) calcd for $C_{21}H_{32}F_3O_3S^+$ $[M + H]^+$ 421.2024, found 421.2019

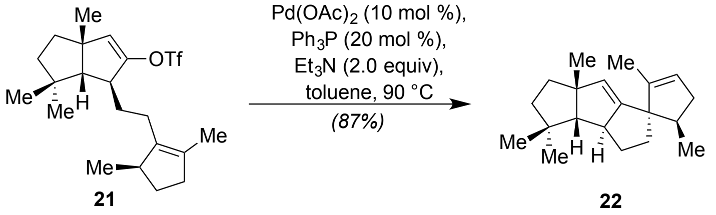

Diene 22. To a flame-dried 4-mL scintillation vial was added triflate **21** (30.0 mg, 0.071 mmol), Ph_3P (3.7 mg, 0.014 mmol, 0.2 equiv), $Pd(OAc)_2$ (1.6 mg, 0.0071 mmol, 0.1 equiv), and toluene (1.2 mL). After sparging the reaction mixture with argon for 30 min at 23 °C, Et_3N (0.0195 mL, 0.14 mmol, 2.0 equiv) was added via syringe, and the vial was sealed with a Teflon-lined cap. The reaction mixture was heated at 90 °C for 20 h. The yellowish-black mixture was cooled to 23 °C and diluted with hexanes (1 mL). The reaction mixture was subjected directly to purification by flash column chromatography (silica gel, hexanes/Et_3N, 99/1) to give diene **22** (16.9 mg, 88% yield) as a colorless oil. **22**: R_f = 0.72 (silica gel, hexanes); $[\alpha]_D^{20}$ = +54.2° (*c* = 1.0, C_6D_6); IR (film) ν_{max} 3035, 2942, 2864, 1458, 1383, 1371, 1013, 856 cm^{-1}; ^1H NMR (500

MHz, $CDCl_3$) δ 5.32 (s, 1 H), 4.86 (d, *J* = 2.9 Hz, 1 H), 2.80 (dtd, *J* = 9.6, 6.6, 3.1 Hz, 1 H), 2.30–2.14 (m, 3 H), 1.97–1.90 (m, 1 H), 1.74–1.61 (m, 3 H), 1.66 (s, 3 H), 1.56–1.48 (m, 3 H), 1.39 (dd, *J* = 10.8, 5.9 Hz, 1 H), 1.29 (s, 3 H), 1.09 (dt, *J* = 12.0, 4.8 Hz, 1 H), 1.03 (s, 3 H), 1.02 (d, *J* = 6.9 Hz, 3 H), 1.00 (s, 3 H); ^{13}C NMR (125 MHz, $CDCl_3$) δ 151.43, 147.37, 128.06, 123.21, 66.29, 63.72, 58.95, 56.85, 47.16, 41.30, 41.06, 40.72, 38.97, 37.89, 32.54, 32.36, 29.21, 26.06, 16.46, 14.36. [Note: Despite efforts to obtain HRMS data no successful ionization was achieved with either ESI or CI].

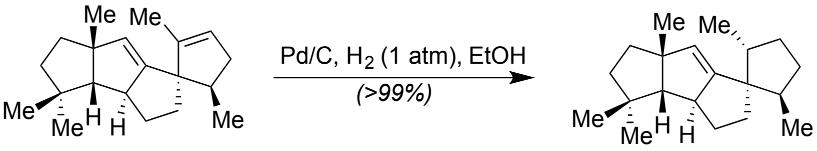

22 **4** [proposed structure]

(–)-Spiroviolene (4).[3] To a 4-mL scintillation vial at 23 °C was added diene **22** (9.0 mg, 0.033 mmol, 1.0 equiv), Pd/C (20.0 mg, 10 wt %, reduced dry powder), and EtOH (0.33 mL). The vial was sealed with a septum and the reaction mixture was sparged with hydrogen for 5 min at 23 °C. The reaction mixture was then stirred for 30 min at 23 °C. Upon completion, the black mixture was filtered through a short plug of Celite and silica gel, rinsing with hexanes (3 × 2 mL). The filtrate was evaporated and purified via flash column chromatography (silica gel, hexanes) to afford (–)-spiroviolene (**4**, 9.1 mg, quantitative) as a colorless oil. **4**: R_f = 0.84 (silica gel, hexanes); $[\alpha]_D^{20}$ = –5.4° (*c* = 0.2, C_6D_6); IR (film) ν_{max} 3032, 2943, 2866, 2361, 1463, 1370, 1260, 853, 808 cm^{-1}; ^{1}H NMR (500 MHz, C_6D_6) δ 4.82 (d, *J* = 2.9 Hz, 1 H), 2.78 (dddd, *J* = 12.3, 6.4, 6.2, 2.9 Hz, 1 H), 1.93 (ddd, *J* = 12.7, 12.7, 6.9 Hz, 1 H), 1.86–1.63 (m, 7 H), 1.64–1.55 (m, 3 H), 1.48–1.35 (m, 3 H), 1.35 (s, 3 H), 1.14–1.07 (m, 1 H), 1.05 (s, 3 H), 1.04 (s, 3 H), 0.98 (d, *J* = 6.7 Hz, 3 H), 0.95 (d, *J* = 6.7 Hz, 3 H); ^{13}C NMR (125 MHz, C_6D_6) δ 148.96, 128.98, 66.13, 63.70, 59.42, 53.79, 46.64, 44.76, 41.27, 40.83, 39.58, 38.60, 33.06, 32.37, 31.32, 30.70, 29.16, 26.10, 15.17, 15.09. [Note: Despite efforts to obtain HRMS data no successful ionization was achieved with either ESI or CI]. All NMR spectral data matched that reported in the literature; see comparison in Table S1.[3] Literature $[\alpha]_D^{21}$ = –5.6° (*c* = 0.2, C_6D_6).[3]

Table S1. Comparison of NMR data of spiroviolene (**4**) between our synthetic sample and the natural isolate.[3]

^{1}H NMR

**Synthetic 4** (500 MHz)	**Natural 4** (500 MHz)
4.82 (d, *J* = 2.9 Hz, 1 H)	4.81 (d, *J* = 2.9 Hz, 1 H)
2.78 (dddd, *J* = 12.3, 6.4, 6.2, 2.9 Hz, 1 H)	2.77 (dddd, *J* = 12.5, 6.4, 6.4, 2.9 Hz, 1 H)
1.93 (ddd, *J* = 12.7, 12.7, 6.9 Hz, 1 H)	1.92 (ddd, *J* = 12.7, 6.9, 6.9 Hz, 1 H)
1.86–1.63 (m, 7 H)	1.81 (m, 1 H)
	1.79 (m, 1 H)
	1.74 (m, 1 H)
	1.73 (m, 1 H)
	1.72 (m, 1 H)
	1.69 (m, 1 H)
	1.67 (m, 1 H)
1.64–1.55 (m, 3 H)	1.60 (m, 1 H)
	1.59 (m, 1 H)
	1.58 (m, 1 H)
1.48–1.35 (m, 3 H)	1.43 (dddd, *J* = 11.8, 6.6, 1.5, 1.5 Hz, 1 H)
	1.38 (m, 1 H)
	1.33 (m, 1 H)
1.35 (s, 3 H)	1.34 (s, 3 H)
1.14–1.07 (m, 1 H)	1.09 (dddd, *J* = 12.2, 12.2, 11.3, 7.6 Hz, 1 H)
1.05 (s, 3 H)	1.04 (s, 3 H)

1.04 (s, 3 H)	1.03 (s, 3 H)
0.98 (d, *J* = 6.7 Hz, 3 H)	0.97 (d, *J* = 6.7 Hz, 3 H)
0.95 (d, *J* = 6.7 Hz, 3 H)	0.94 (d, *J* = 6.7 Hz, 3 H)
7.16 (s, C_6D_6)	7.16 (s, C_6D_6)

^{13}C NMR

**Synthetic 4** (500 MHz)	**Natural 4** (500 MHz)
148.96	148.9
128.98	128.9
66.13	66.0
63.70	63.7
59.42	59.4
53.79	53.8
46.64	46.6
44.83	44.7
41.27	41.3
40.83	40.8
39.58	39.5
38.60	38.6
33.06	33.1
32.37	32.4
31.32	31.3

30.70	30.7
29.16	29.1
26.10	26.1
15.17	15.2
15.09	15.1
128.06 (s, C_6D_6)	128.06 (s, C_6D_6)

Total Synthesis of Spirograterpene A (5)

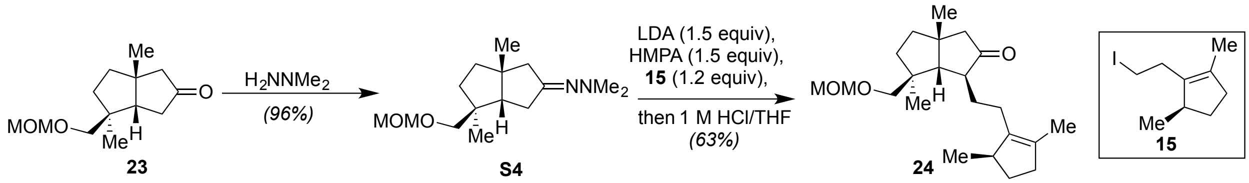

Bicyclic Ketone 24. To a flask containing **23**[2] (1.37 g, 6.05 mmol, 1.0 equiv) was added *N,N*-dimethyl hydrazine (1.38 mL, 18.15 mmol, 3.0 equiv) and the resultant mixture stirred at 23 °C for 12 h. Upon completion, the reaction contents were quenched with NH_4Cl (5 mL) and diluted with Et_2O (5 mL). After stirring for 30 min, the resultant mixture was transferred to a separatory funnel and further diluted with NH_4Cl (30 mL) and Et_2O (50 mL). The organic layer was separated and further washed with NH_4Cl (2 × 30 mL), H_2O (30 mL), brine (30 mL), dried (Na_2SO_4) and concentrated to give hydrazone **S4** (1.56 g, 96% yield) as a yellow oil. Pressing forward without any further purification, to a flame-dried, 250 mL Schlenk flask was added solid LDA in a glovebox at 23 °C. The flask was then transferred from the glovebox, attached to a Schlenk manifold, and placed under positive pressure of N_2. THF (35 mL) was then added and the flask was cooled to –78 °C. After all solids had dissolved, freshly prepared **S4** was added as a solution in THF (20 mL) slowly over the course of 5 min. Once that addition was complete, the flask was then warmed to 0 °C and the contents were stirred for 2 h. The reaction contents were then recooled to –78 °C and HMPA (1.51 mL, 8.70 mmol, 1.5 equiv) was added dropwise. After stirring at –78 °C for 30 min, a solution of alkyl iodide **15** (1.74 g, 7.00 mmol, 1.2 equiv) in THF (5 mL) was added over the course of 5 min. The reaction contents were then allowed to slowly warm to 23 °C over the course of 5 h and were stirred at 23 °C for an additional 10 h. Upon completion, the reaction contents were quenched by the addition of 1 N HCl (50 mL) and stirred under a N_2 atmosphere for an additional 12 h. Upon completion, the reaction mixture was diluted with Et_2O (50 mL) and transferred to a separatory funnel. After separating the resultant layers,

the aqueous layer was extracted with Et_2O (2 × 50 mL). The combined organic layers were washed with saturated aqueous $NaHCO_3$ (30 mL), H_2O (2 × 30 mL) and brine (30 mL), dried (Na_2SO_4) and concentrated. Purification of the resultant yellow oil via flash column chromatography (silica gel, hexanes/EtOAc, 4/1) gave alkylated product **24** (1.43 g, 63% yield) as a pale yellow oil. **24**: R_f = 0.50 (silica gel, hexanes/EtOAc, 4/1); $[\alpha]_D^{20}$ = +0.9° (*c* = 0.75, $CHCl_3$); IR (film) ν_{max} 2948, 2865, 1737, 1454, 1148, 1110, 1048 cm^{-1}; ^1H NMR (500 MHz, $CDCl_3$) δ 4.63 (s, 2 H), 3.36 (s, 3 H), 3.35–3.29 (m, 2 H), 2.68–2.58 (m, 1 H), 2.33 (dd, *J* = 18.1, 1.8 Hz, 1 H), 2.22 (q, *J* = 6.6 Hz, 3 H), 2.19–2.09 (m, 1 H), 1.98 (ddt, *J* = 12.0, 7.3, 3.6 Hz, 2 H), 1.81–1.76 (m, 3 H), 1.67 (td, *J* = 6.3, 2.5 Hz, 2 H), 1.61 (dd, *J* = 2.1, 1.1 Hz, 3 H), 1.49 (dt, *J* = 12.6, 6.3 Hz, 1 H), 1.33–1.23 (m, 4 H), 1.18 (s, 3 H), 0.96 (d, *J* = 6.9 Hz, 3 H), 0.95 (s, 3 H); ^{13}C NMR (126 MHz, $CDCl_3$) δ 221.52, 138.97, 131.92, 96.85, 76.66, 59.24, 55.32, 52.98, 50.46, 46.33, 45.13, 41.54, 39.28, 36.86, 36.71, 31.39, 31.19, 30.86, 24.29, 21.21, 19.77, 14.22; HRMS (ESI) calcd for $C_{22}H_{36}O_3^+$ $[M]^+$ 348.2700, found 348.2694.

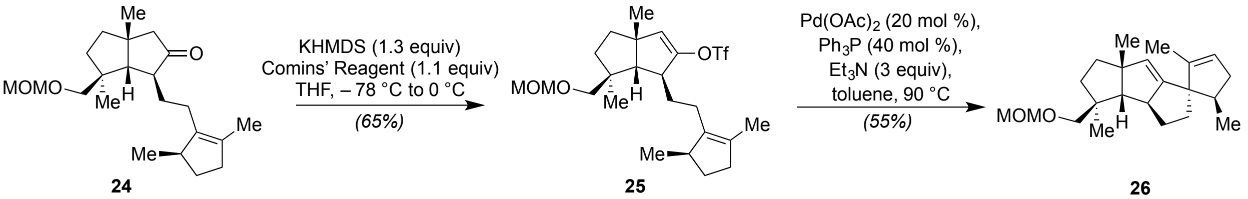

Diene 26. To a flame-dried, 50 mL flask was added ketone **24** (0.593 g, 1.70 mmol, 1.0 equiv) and THF (17 mL) at 23 °C. The flask was cooled to –78 °C and KHMDS (4.42 mL, 0.5 M in toluene, 2.2 mmol, 1.3 equiv) was added dropwise. The reaction contents were warmed to 0 °C and stirred for 2 h. The reaction mixture was recooled to –78 °C and a solution of Comins' reagent (0.734 g, 1.87 mmol, 1.1 equiv) in THF (3 mL) was added over the course of 10 min. The reaction was maintained at –78 °C for 20 min before being warmed to 23 °C and stirred for 1 h. Upon completion the reaction was quenched with saturated aqueous $NaHCO_3$ (5 mL) and diluted with Et_2O (20 mL) and H_2O (10 mL). The mixture was then transferred to a separatory funnel and the aqueous layer was extracted with Et_2O (2 × 15 mL). The combined organic layers were washed with H_2O (2 × 25 mL) and brine (25 mL), dried (Na_2SO_4), and concentrated. The resultant yellow oil was further purified by flash column chromatography (silica gel, hexanes/EtOAc, 20/1) to give **25** (0.531 g, 65% yield) as a colorless oil. Next, to a flame-dried, 20-mL pressure vessel at 23 °C were added $Pd(OAc)_2$ (49.4 mg, 0.22 mmol, 0.2 equiv), Ph_3P (0.115 g, 0.44 mmol, 0.4 equiv) and Et_3N (0.460 mL, 3.3 mmol, 3.0 equiv). The contents of the flask were suspended in toluene (8 mL) and Ar was bubbled through the reaction mixture for 15 min. A solution of triflate **25** (0.528 g, 1.1 mmol, 1.0 equiv) in toluene (3 mL) was then added at 23 °C and the reaction mixture was heated to 90 °C for 16 h. Upon completion, the reaction contents were diluted with hexanes (5 mL) and directly purified via flash column chromatography (silica gel, hexanes/EtOAc, 20/1) to give the desired cyclization product **26** (0.200 g, 55% yield) as a pale yellow oil. **26**: R_f = 0.68 (silica gel, hexanes/EtOAc, 9/1); $[\alpha]_D^{20}$ = +23.6° (*c* = 0.33, $CHCl_3$); IR (film) ν_{max} 3034, 2928, 2863, 1449, 1373, 1110, 1049 cm^{-1}; ^1H

NMR (500 MHz, $CDCl_3$) δ 5.32 (p, J = 1.7 Hz, 1 H), 4.89 (dd, J = 3.0, 0.7 Hz, 1 H), 4.64 (s, 2 H), 3.38 (s, 3 H), 3.36 (q, J = 9.0 Hz, 2 H), 2.89 (dtd, J = 12.3, 6.6, 3.0 Hz, 1 H), 2.27 (dddd, J = 13.3, 7.5, 2.4, 1.3 Hz, 1 H), 2.24–2.15 (m, 2 H), 1.94 (ddt, J = 14.9, 8.3, 2.3 Hz, 1 H), 1.78 (d, J = 6.7 Hz, 1 H), 1.76–1.65 (m, 6 H), 1.55–1.40 (m, 2 H), 1.25 (s, 3 H), 1.07 (s, 3 H), 1.02 (d, J = 6.9 Hz, 3 H); ^{13}C NMR (126 MHz, $CDCl_3$) δ 151.63, 147.25, 127.80, 123.32, 96.88, 75.35, 63.67, 61.65, 58.03, 56.83, 55.26, 47.16, 45.33, 41.19, 38.93, 37.35, 36.96, 32.48, 31.66, 21.70, 16.45, 14.40; HRMS (ESI) calcd for $C_{22}H_{34}O_2^+$ $[M]^+$ 330.2559, found 330.2557.

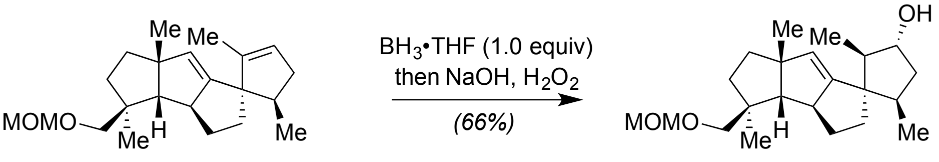

Secondary Alcohol 27. To a flame-dried, 5-mL pressure vessel at 23 °C was added **26** (129.0 mg, 0.39 mmol, 1.0 equiv) and THF (2 mL). The contents of the vessel were then cooled to 0 °C and BH_3•THF (0.390 mL, 1.0 M in THF, 0.39 mmol, 1.0 equiv) was added. The reaction was maintained at 0 °C with stirring and after 5 h, NaOH (0.33 mL, 6 M aqueous) and H_2O_2 (0.33 mL, 30% w/w) were added simultaneously. The reaction mixture was then warmed to 23 °C and stirred for 1 h, after which time it was diluted with Et_2O (10 mL) and H_2O (10 mL). The layers were separated and the aqueous layer was further extracted with Et_2O (2 × 10 mL). The combined organic layers were washed with brine (15 mL), dried (Na_2SO_4), and concentrated to give a yellow oil. This crude material was further purified by flash column chromatography (silica gel, hexanes/EtOAc, 6/1) to give alcohol **27** (89.7 mg, 66% yield) as a colorless oil. **27**: R_f = 0.33 (silica gel, hexanes/EtOAc, 4/1); $[\alpha]_D^{20}$ = –6.7° (c = 0.67, $CHCl_3$); IR (film) ν_{max} 3362, 3030, 2931, 2869, 1456, 1371, 1111, 1050 cm^{-1}; ^1H NMR (500 MHz, $CDCl_3$) δ 4.80 (d, J = 2.9 Hz, 1 H), 4.64 (s, 2 H), 3.85 (td, J = 8.8, 3.8 Hz, 1 H), 3.37 (s, 3 H), 3.34 (d, J = 12.4 Hz, 2 H), 2.76 (dtd, J = 12.7, 6.5, 2.9 Hz, 1 H), 2.14 (ddq, J = 13.5, 10.4, 6.7 Hz, 1 H), 1.95 (td, J = 12.9, 7.0 Hz, 1 H), 1.86–1.62 (m, 8 H), 1.61–1.53 (m, 2 H), 1.52–1.42 (m, 2 H), 1.24 (s, 3 H), 1.05 (s, 3 H), 0.99 (d, J = 6.8 Hz, 3 H), 0.87 (d, J = 6.8 Hz, 3 H); ^{13}C NMR (126 MHz, $CDCl_3$) δ 148.98, 128.77, 96.87, 78.55, 75.17, 63.48, 61.14, 58.28, 55.26, 53.20, 53.10, 45.55, 43.11, 41.12, 39.65, 37.77, 36.73, 32.52, 31.57, 21.60, 14.80, 12.62; HRMS (ESI) calcd for $C_{22}H_{36}O_3^+$ $[M]^+$ 348.2664, found 348.2664.

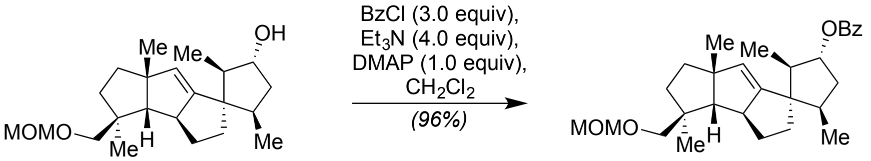

Benzoate S5. To a flame-dried, 4-mL vial at 23 °C was added **27** (19.8 mg, 0.057 mmol, 1.0 equiv) and CH_2Cl_2 (0.6 mL). To this solution was then added BzCl (0.020 mL, 0.17 mmol, 3.0 equiv), Et_3N (0.032 mL, 0.228 mmol, 4.0 equiv) and 4-DMAP (7.0 mg, 0.057 mmol, 1.0 equiv). The reaction mixture was stirred at 23 °C for 2 h. Upon completion, the reaction

contents were quenched with saturated aqueous NH_4Cl (1 mL) and diluted with CH_2Cl_2 (5 mL) and H_2O (5 mL). The layers were separated and the aqueous layer was extracted with CH_2Cl_2 (3 × 5 mL). The combined organic layers were dried (Na_2SO_4) and concentrated to give an orange oil. Purification of this crude material by flash column chromatography (silica gel, hexanes/EtOAc, 10/1) gave the desired benzoate **S5** (24.7 mg, 96% yield) as a pale yellow oil. **S5**: R_f = 0.73 (silica gel, hexanes/EtOAc, 4/1); $[\alpha]_D^{20}$ = –44.3° (*c* = 0.28, $CHCl_3$); IR (film) ν_{max} 2927, 2871, 1718, 1279, 1113, 1047, 712 cm^{-1}; ^1H NMR (500 MHz, $CDCl_3$ δ 8.04 (d, *J* = 7.9 Hz, 2 H), 7.55 (t, *J* = 7.2 Hz, 1 H), 7.44 (t, *J* = 7.5 Hz, 2 H), 5.01–4.93 (m, 2 H), 4.65 (s, 2 H), 3.38 (s, 3 H), 3.35 (d, *J* = 11.6 Hz, 2 H), 2.79 (dd, *J* = 12.8, 6.7 Hz, 1 H), 2.23–2.14 (m, 1 H), 2.03 (qd, *J* = 13.3, 12.4, 8.6 Hz, 3 H), 1.89–1.66 (m, 6 H), 1.59–1.46 (m, 3 H), 1.26 (s, 3 H), 1.06 (s, 3 H), 1.03 (d, *J* = 6.8 Hz, 3 H), 0.92 (d, *J* = 6.7 Hz, 3 H); ^{13}C NMR (126 MHz, $CDCl_3$) δ 166.98, 148.31, 132.85, 129.69, 129.21, 128.43, 96.93, 81.37, 75.28, 63.68, 61.26, 58.34, 55.27, 52.57, 49.91, 45.56, 43.54, 39.63, 38.74, 37.72, 36.83, 32.53, 31.53, 29.85, 21.65, 14.59, 12.72; HRMS (ESI) calcd for $C_{29}H_{40}O_4^+$ $[M]^+$ 452.2927, found 452.2943.

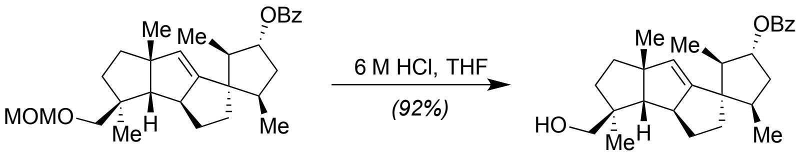

S5 **28**

Primary Alcohol 28. To a 4-mL vial at 23 °C containing a solution of MOM ether **S5** (57.0 mg, 0.126 mmol, 1.0 equiv) in THF (2.5 mL) was added 6 M HCl (1.5 mL) dropwise. The reaction mixture was then heated to 50 °C for 4 h. Upon completion, the reaction contents were cooled to 23 °C and diluted with brine (5 mL). The resultant layers were separated and the aqueous layer was then extracted with Et_2O (3 × 5 mL). The combined organic layers were then dried (Na_2SO_4) and concentrated. Purification of the resultant residue by flash column chromatography (silica gel, hexanes/EtOAc, 5/1) gave alcohol **28** (47.5 mg, 92% yield) as a colorless oil. **28**: R_f = 0.29 (silica gel, hexanes/EtOAc, 4/1); $[\alpha]_D^{20}$ = –17.5° (*c* = 0.35, MeOH); IR (film) ν_{max} 3417, 2952, 2923, 2870, 1718, 1704, 1452, 1281, 1115, 712 cm^{-1}; ^1H NMR (500 MHz, $CDCl_3$) δ 8.04 (d, *J* = 7.2 Hz, 2 H), 7.55 (t, *J* = 7.4 Hz, 1 H), 7.43 (t, *J* = 7.7 Hz, 2 H), 4.98 (d, *J* = 2.9 Hz, 1 H), 4.98–4.94 (m, 1 H), 3.51 (d, *J* = 10.6 Hz, 1 H), 3.41 (d, *J* = 10.6 Hz, 1 H), 2.80 (dtd, *J* = 12.7, 6.5, 2.8 Hz, 1 H), 2.23–2.15 (m, 1 H), 2.08–1.97 (m, 3 H), 1.89–1.77 (m, 2 H), 1.76–1.63 (m, 5 H), 1.53–1.43 (m, 2 H), 1.26 (s, 3 H), 1.04 (s, 3 H), 1.03 (d, *J* = 6.7 Hz, 3 H), 0.92 (d, *J* = 6.7 Hz, 3 H); ^{13}C NMR (126 MHz, $CDCl_3$) δ 166.98, 148.34, 132.86, 129.67, 129.10, 128.42, 81.33, 70.26, 63.72, 60.97, 58.30, 52.55, 49.84, 46.65, 43.50, 39.58, 38.69, 37.56, 36.30, 32.48, 31.38, 21.00, 14.58, 12.69; HRMS (ESI) calcd for $C_{54}H_{69}O_4^+$ $[2M + H]^+$ 845.4992, found 845.4984.

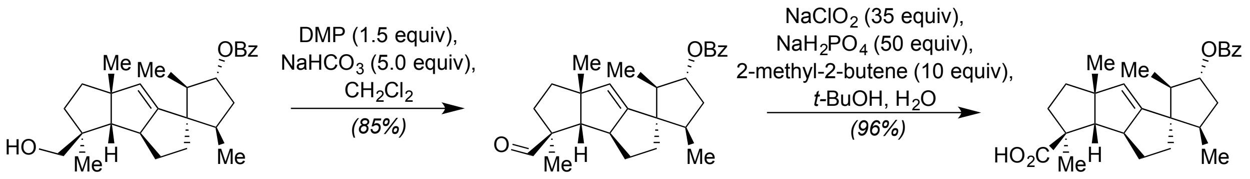

Carboxylic Acid 29. To a flame-dried, 4-mL vial at 23 °C was added **28** (42.8 mg, 0.105 mmol, 1.0 equiv), $NaHCO_3$ (44.1 mg, 0.525 mmol, 5.0 equiv) and CH_2Cl_2 (2 mL). To this suspension was added Dess–Martin periodinane (66.8 mg, 0.158 mmol, 1.5 equiv). After stirring the resultant mixture at 23 °C for 1 h, the reaction contents were quenched with a 1:1 mixture of saturated aqueous $NaHCO_3$ (2.5 mL) and $Na_2S_2O_3$ (2.5 mL). The resultant biphasic mixture was then stirred vigorously for 30 min after which time the resultant layers were separated and the aqueous layer was extracted with Et_2O (3 × 5 mL). The combined organic layers were then dried (Na_2SO_4) and concentrated to give a yellow oil which was further purified by flash column chromatography (silica gel, hexanes/EtOAc, 20/1) to give aldehyde **S6** (35.8 mg, 85% yield) as a colorless oil. Next, to a 4-mL vial at 23 °C containing a solution of aldehyde **S6** (35.8 mg, 0.088 mol, 1.0 equiv) and 2-methyl-2-butene (0.932 mL, 0.88 mmol, 10.0 equiv) in *t*-BuOH (1 mL) was added a solution of $NaClO_2$ (294.0 mg, 3.26 mmol, 35.0 equiv) and NaH_2PO_4 (528.0 mg, 4.40 mmol, 50.0 equiv) in H_2O (2 mL). The reaction mixture was stirred at 23 °C for 1 h after which time the reaction mixture was diluted with H_2O (5 mL) and EtOAc (5 mL). The layers were separated and the aqueous layer was extracted with EtOAc (3 × 5 mL). The combined organic layers were dried (Na_2SO_4) and concentrated to give pale yellow residue. Purification of that crude material by flash column chromatography (silica gel, hexanes/EtOAc, 5/1) gave carboxylic acid **29** (35.0 mg, 96% yield) as a pale yellow oil. **29**: R_f = 0.32 (silica gel, hexanes/EtOAc, 6/1); $[\alpha]_D^{20}$ = –20.4° (*c* = 0.46, MeOH); IR (film) ν_{max} 2953, 2932, 2869, 1717, 1698, 1452, 1280, 1114, 713 cm^{-1}; ^1H NMR (500 MHz, $CDCl_3$) δ 8.04 (d, *J* = 7.3 Hz, 2 H), 7.55 (t, *J* = 7.4 Hz, 1 H), 7.44 (t, *J* = 7.7 Hz, 2 H), 5.02 (d, *J* = 2.7 Hz, 1 H), 4.96 (td, *J* = 9.1, 3.5 Hz, 1 H), 2.73 (dtd, *J* = 12.7, 6.6, 2.8 Hz, 1 H), 2.47 (dd, *J* = 6.5, 1.4 Hz, 1 H), 2.30 (ddd, *J* = 13.8, 7.0, 3.9 Hz, 1 H), 2.23–2.14 (m, 1 H), 2.03 (tdd, *J* = 13.2, 8.5, 4.8 Hz, 3 H), 1.92–1.66 (m, 4 H), 1.61–1.43 (m, 3 H), 1.31 (s, 3 H), 1.21 (s, 3 H), 1.03 (d, *J* = 6.8 Hz, 3 H), 0.92 (d, *J* = 6.7 Hz, 3 H); ^{13}C NMR (126 MHz, $CDCl_3$) δ 184.45, 166.98, 148.13, 132.89, 129.67, 129.26, 128.43, 81.25, 63.38, 61.72, 58.33, 52.49, 52.22, 49.81, 43.51, 39.74, 38.68, 38.35, 36.85, 32.39, 31.33, 30.37, 21.77, 14.59, 12.68; HRMS (ESI) calcd for $C_{54}H_{70}O_2^+$ $[2M - H_2O]^+$ 798.5223, found 798.5219.

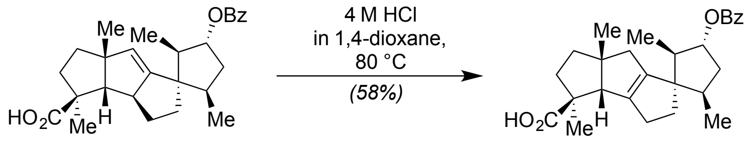

29 → **30**

Tetrasubstituted Alkene 30. To a flame-dried, 4-mL vial at 23 °C was added carboxylic acid **29** (19.9 mg, 0.047 mmol, 1.0 equiv) and HCl (1 mL, 4 M in dioxane). The reaction mixture was then heated to 80 °C for 48 h. Upon completion, the mixture was diluted with H_2O (5 mL) and EtOAc (5 mL). The layers were then separated and the aqueous layer was extracted with EtOAc (3 × 5 mL). The combined organic layers were dried (Na_2SO_4) and concentrated to give a brown oil. Purificaiton of that residue by flash column chromatography (silica gel, hexanes/EtOAc, 5/1) gave the desired isomerized product **30** (11.5 mg, 58% yield) as a pale yellow oil. **30**: R_f = 0.55 (silica gel, hexanes/EtOAc, 2/1); $[\alpha]_D^{20}$ = –8.5° (*c* = 0.15, MeOH); IR (film) ν_{max} 2953, 2929, 2869, 1717, 1695, 1279, 1113 cm^{-1}; ^1H NMR (500 MHz, $CDCl_3$) δ 8.05 (d, *J* = 8.4 Hz, 2 H), 7.55 (t, *J* = 7.4 Hz, 1 H), 7.44 (t, *J* = 7.7 Hz, 2 H), 5.00 (td, *J* = 9.1, 3.7 Hz, 1 H), 2.83 (d, *J* = 2.3 Hz, 1 H), 2.32–1.81 (m, 11 H), 1.71–1.54 (m, 3 H), 1.23 (s, 3 H), 1.18 (s, 3 H), 1.00 (d, *J* = 6.9 Hz, 3 H), 0.87 (d, *J* = 6.8 Hz, 3 H); ^{13}C NMR (126 MHz, $CDCl_3$) δ 183.90, 166.94, 147.96, 145.95, 132.89, 130.90, 129.69, 128.44, 80.96, 61.95, 59.48, 56.97, 52.85, 50.53, 47.40, 43.11, 39.63, 39.40, 38.59, 38.38, 30.10, 29.70, 20.94, 14.44, 13.34; HRMS (ESI) calcd for $C_{27}H_{34}O_4Na^+$ $[M + Na]^+$ 445.2354, found 445.2344.

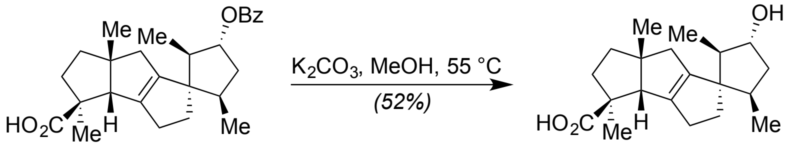

30 → **5**

Spirograterpene A (5).[5] To a solution of **30** (11.5 mg, 0.027 mmol, 1.0 equiv) in MeOH (0.25 mL) in a 4-mL vial was added K_2CO_3 (11.2 mg, 0.081 mmol, 3.0 equiv). The vial was sealed and the reaction contents heated to 55 °C. After 15 h, the reaction was quenched by the addition of NH_4Cl (1 mL). The aqueous layer was extracted with Et_2O (3 × 2 mL), dried (Na_2SO_4) and concentrated to give a yellow oil. This crude material was further purified by flash column chromatography (silica gel, hexanes/EtOAc, 2/1) to give **5** (4.7 mg, 52% yield). **5**: R_f = 0.29 (silica gel, hexanes/EtOAc, 2/1); $[\alpha]_D^{20}$ = –18.0° (*c* = 0.11, MeOH); IR (film) ν_{max} 3400, 3313, 3203, 2960, 2925, 2855, 1698, 1373, 1260, 1028, 800, 668 cm^{-1}; ^1H NMR (500 MHz, CD_3OD) δ 3.78 (dt, *J* = 8.4, 6.8 Hz, 1 H), 2.80 (d, *J* = 2.6 Hz, 1 H), 2.30–2.21 (m, 1 H), 2.20–2.11 (m, 5 H), 2.10–2.04 (m, 1 H), 2.03–1.97 (m, 1 H), 1.73 (dd, *J* = 10.4, 6.8 Hz, 2 H), 1.69–1.60 (m, 2 H), 1.59–1.50 (m, 2 H), 1.19 (s, 3 H), 1.15 (s, 3 H), 0.98 (d, *J* = 6.9 Hz, 4 H), 0.85 (d, *J* = 6.9 Hz, 3 H); ^{13}C NMR (126 MHz, CD_3OD) δ 182.54, 149.20, 147.25, 78.54, 63.08, 61.00, 57.72, 54.87, 54.37, 48.60, 44.02, 42.59, 40.65, 39.49, 30.64, 30.41, 21.79, 14.93, 13.53; HRMS (ESI) calcd for $C_{20}H_{30}O_3^+$ $[M]^+$ 318.2915, found 318.2194. All NMR spectral data matched that reported in the literature; see comparison in Table S2.[5] Literature $[\alpha]_D^{25}$ = –22.2° (*c* = 0.35, MeOH).[5]

Table S2. Comparison of NMR data of spirograterpene A (**5**) between our synthetic sample and the natural isolate.[5]

^{1}H NMR

<table>
<tr><th>Synthetic 5 (500 MHz)</th><th>Natural 5 (600 MHz)</th></tr>
<tr><td>3.80 (dt, J = 8.4, 6.8 Hz, 1 H)</td><td>3.80 (dt, J = 8.1, 7.0 Hz, 1 H)</td></tr>
<tr><td>2.82 (d, J = 2.6, Hz 1 H)</td><td>2.81 (d, J = 1.8, Hz 1 H)</td></tr>
<tr><td>2.30–2.21 (m, 1 H)</td><td>2.28 (m, 1 H)</td></tr>
<tr><td rowspan="4">2.20–2.11 (m, 5 H)</td><td>2.17 (m, 1 H)</td></tr>
<tr><td>2.16 (m, 1 H)</td></tr>
<tr><td>2.14–2.19 (m, 2 H)</td></tr>
<tr><td>2.12 (m, 1 H)</td></tr>
<tr><td>2.10–2.04 (m, 1 H)</td><td>2.06 (m, 1 H)</td></tr>
<tr><td>2.03–1.97 (m, 1 H)</td><td>2.00 (dt, J = 16.1, 2.5 Hz, 1 H)</td></tr>
<tr><td>1.73 (dd, J = 10.4, 6.8 Hz, 2 H)</td><td>1.74–1.76 (m, 2 H)</td></tr>
<tr><td rowspan="2">1.69–1.60 (m, 2 H)</td><td>1.68 (m, 1 H)</td></tr>
<tr><td>1.65 (m, 1 H)</td></tr>
<tr><td rowspan="2">1.59–1.50 (m, 2 H)</td><td>1.57 (m, 1 H)</td></tr>
<tr><td>1.57 (m, 1 H)</td></tr>
<tr><td>1.21 (s, 3 H)</td><td>1.22 (s, 3 H)</td></tr>
<tr><td>1.17 (s, 3 H)</td><td>1.17 (s, 3 H)</td></tr>
<tr><td>1.00 (d, J = 6.9, Hz 3 H)</td><td>1.00 (d, J = 6.9, Hz 3 H)</td></tr>
<tr><td>0.87 (d, J = 6.9, Hz 3 H)</td><td>0.87 (d, J = 6.9, Hz 3 H)</td></tr>
<tr><td>3.33 (p, CD_3OD)</td><td>3.33 (p, CD_3OD)</td></tr>
</table>

^{13}C NMR

**Synthetic 5** (500 MHz)	**Natural 5** (600 MHz)
182.54	182.30
149.20	149.07
147.25	147.33
78.54	78.51
63.08	63.02
61.00	60.98
57.72	57.72
54.87	54.83
54.37	54.23
48.60	48.57
44.02	44.01
42.59	42.56
40.65	40.61
39.49	39.46
30.64	30.62
30.41	30.40
21.79	21.72
14.93	14.95
13.53	13.54
49.00 (p, CD_3OD)	49.00 (p, CD_3OD)

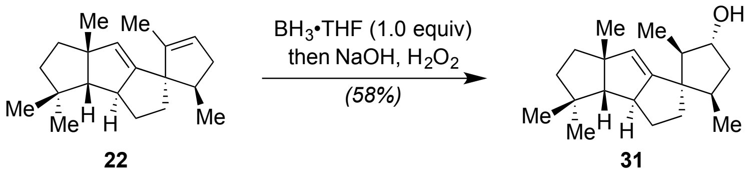

Alcohol 31. To a flame-dried 10 mL vial was added diene **22** (14.0 mg, 0.05 mmol, 1.0 equiv) and THF (0.25 mL). The reaction mixture was cooled to 0 °C and BH_3•THF (0.05 mL, 0.05 mmol, 1.0 equiv, 1.0 M in THF) was added. The reaction was stirred at this temperature for 4 h, after which NaOH (0.05 mL, 6 M aqueous) and H_2O_2 (0.05 mL, 30% w/w) were added simultaneously. The reaction mixture was warmed to 23 °C and stirred for 1 h, after which it was diluted with Et_2O (1 mL) and H_2O (1 mL). The layers were separated and the aqueous layer was further extracted with Et_2O (3 × 1 mL). The combined organic layers were washed with brine (1 mL), dried (Na_2SO_4) and concentrated to give a yellow oil. The crude material was further purified by preparative TLC (silica gel, hexanes/EtOAc, 4/1) to give alcohol **31** (7.7 mg, 54% yield) as a colorless oil. **31**: R_f = 0.20 (silica gel, hexanes/EtOAc, 4/1); ^{1}H NMR (500 MHz, $CDCl_3$) δ 4.77 (d, *J* = 2.7 Hz, 1 H), 3.85 (td, *J* = 9.4, 8.9, 3.6 Hz, 1 H), 2.68 (dtd, *J* = 12.5, 6.4, 2.8 Hz, 1H), 2.21–2.09 (m, 1 H), 1.99–1.90 (m, 1 H), 1.85–1.74 (m, 2 H), 1.72–1.64 (m, 2 H), 1.62 (td, *J* = 4.2, 1.6 Hz, 1 H), 1.60–1.56 (m, 1 H), 1.55–1.50 (m, 3 H), 1.44 (d, *J* = 11.0 Hz, 1 H), 1.41–1.37 (m, 1 H), 1.28 (d, *J* = 1.6 Hz, 3 H), 1.02 (d, *J* = 1.5 Hz, 3 H), 1.00–0.97 (m, 6 H), 0.87 (d, *J* = 6.8 Hz, 3 H).

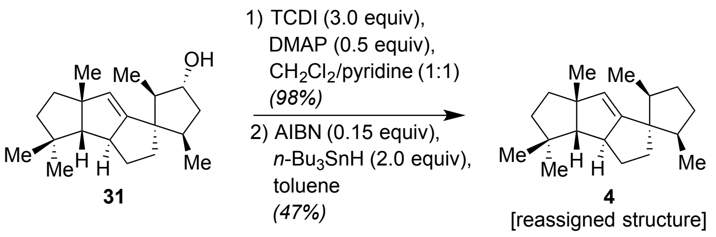

Barton Deoxygenation to Spiroviolene (4). To a 4-mL scintillation vial at 23 °C was added alcohol **31** (7.7 mg, 0.027 mmol, 1.0 equiv), 1,1'-thiocarbonyldiimidazole (14.4 mg, 0.081 mmol, 3.0 equiv) and 4-DMAP (1.7 mg, 0.0135 mmol, 0.5 equiv). This mixture was dissolved in CH_2Cl_2/pyridine (0.6 mL, 1:1) and the solution was then stirred for 12 h at 23 °C. Once complete, the contents were directly purified by column chromatography (silica gel, hexanes/EtOAc, 19/1) to afford the desired thionoimidazolide intermediate (10.6 mg, 98% yield) as a colorless oil. Next, to a 4-mL scintillation vial at 23 °C was added the resulting thionoimidazole (10.6 mg, 0.027 mmol, 1.0 equiv) and toluene (0.6 mL). To this solution was then added tri-*n*-butyltin hydride (0.015 mL, 0.054 mmol, 2.0 equiv) and AIBN (0.7 mg, 0.0041 mmol, 0.15 equiv). The resultant mixture was heated to 110 °C for 10 min. Upon completion, the reaction contents were cooled to 23 °C, the reaction mixture was concentrated directly, and then purified by flash column chromatography (silica gel, hexanes) to afford deoxygenated product **4** (3.6 mg, 47% yield) whose spectral data matched that of natural spiroviolene (see Figures S1 and S2).[3,4]

Figure S1. ^{1}H NMR comparison of the Barton product (top) to synthetic **4** (bottom).

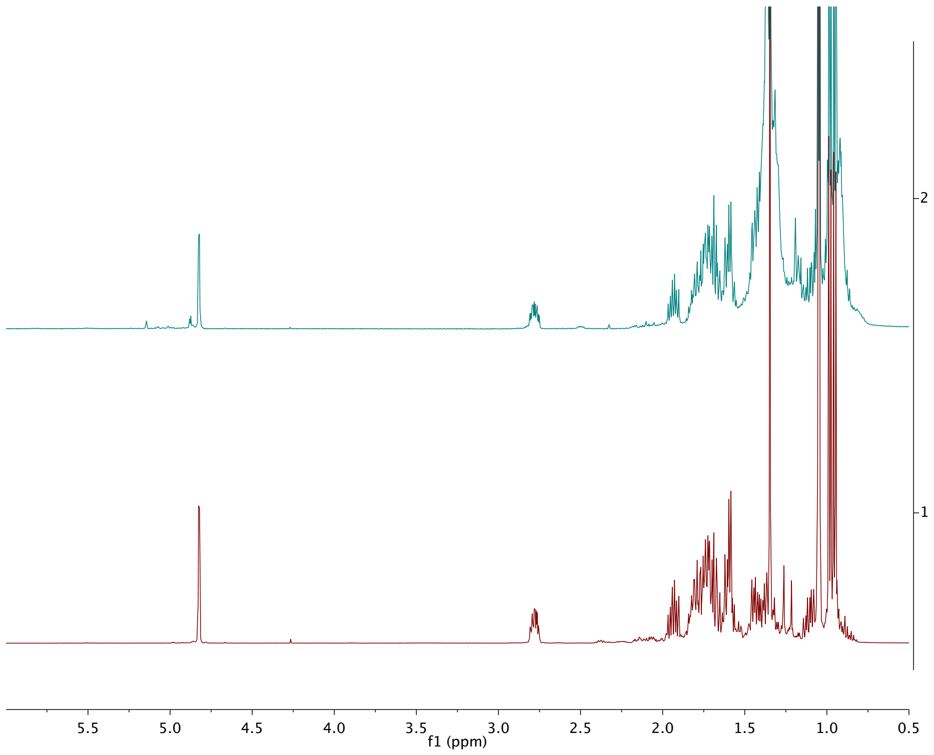

Figure S2. ^{13}C NMR comparison of the Barton product (top) to synthetic **4** (bottom).

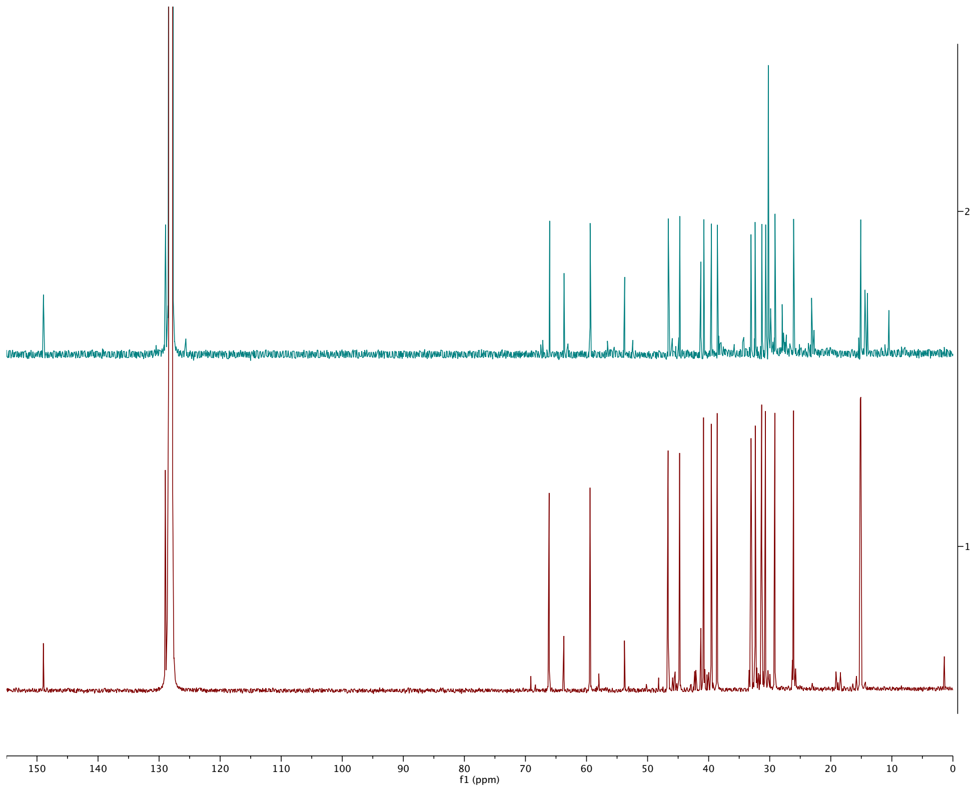

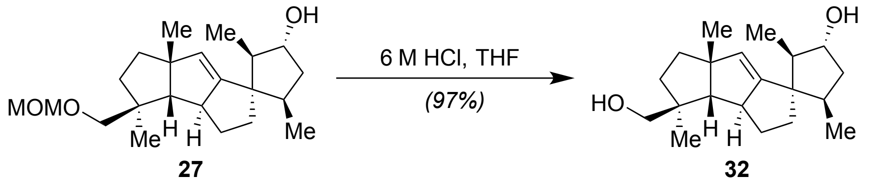

Diol (32). To a 4-mL vial at 23 °C containing a solution of MOM ether **27** (12.0 mg, 0.034 mmol, 1.0 equiv) in THF (0.7 mL) was added 6 M HCl (0.3 mL) dropwise. The reaction mixture was then heated to 50 °C for 4 h. Upon completion, the reaction contents were cooled to 23 °C and diluted with brine (5 mL). The resultant layers were separated and the aqueous layer was then extracted with Et_2O (3 × 5 mL). The combined organic layers were then dried (Na_2SO_4) and concentrated. Purification of the resultant residue by flash column chromatography (silica gel, hexanes/EtOAc, 2/1) gave alcohol **32** (10.1 mg, 97% yield) as a colorless oil. **32**: R_f = 0.12 (silica gel, hexanes/EtOAc, 4/1); ^{1}H NMR (400 MHz, $CDCl_3$) δ 4.81 (d, *J* = 2.9 Hz, 1 H), 3.85 (td, *J* = 8.7, 3.9 Hz, 1 H), 3.50 (d, *J* = 10.6 Hz, 1 H), 3.40 (d, *J* = 10.7 Hz, 1 H), 2.76 (dtd, *J* = 12.5, 6.4, 2.8 Hz, 1 H), 2.22–2.08 (m, 1 H), 1.95 (td, *J* = 12.9, 7.0 Hz, 1 H), 1.86–1.75 (m, 2 H), 1.75–1.63 (m, 5 H), 1.63–1.57 (m, 1 H), 1.53–1.41 (m, 3 H), 1.23 (s, 3 H), 1.03 (s, 3 H), 0.99 (d, *J* = 6.7 Hz, 3 H), 0.87 (d, *J* = 6.8 Hz, 3 H).

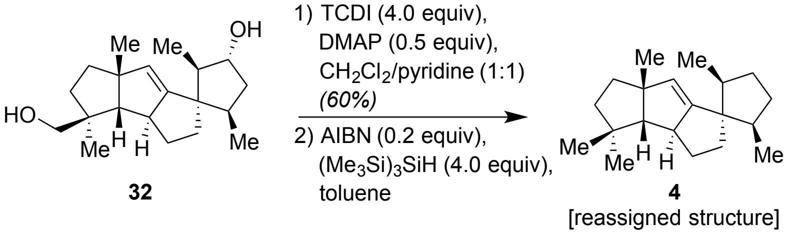

Double Barton Deoxygenation to Spiroviolene (4). To a 4-mL scintillation vial at 23 °C was added alcohol **32** (5.3 mg, 0.017 mmol, 1.0 equiv), 1,1'-thiocarbonyldiimidazole (12.4 mg, 0.070 mmol, 4.0 equiv) and 4-DMAP (1.1 mg, 0.009 mmol, 0.5 equiv). This mixture was dissolved in CH_2Cl_2/pyridine (0.6 mL, 1:1) and the solution was then stirred for 18 h at 23 °C. Once complete, the contents were directly purified by column chromatography (silica gel, hexanes/EtOAc, 3/2) to afford the desired thionoimidazolide intermediate (4.3 mg, 52% yield) as a pale yellow oil. Next, to a 4-mL scintillation vial at 23 °C was added the resulting bis-thionoimidazole (4.3 mg, 0.009 mmol, 1.0 equiv) and toluene (0.2 mL). To this solution was then added tris(trimethyl)silane (0.010 mL, 0.033 mmol, 4.0 equiv) and AIBN (0.3 mg, 0.002 mmol, 0.2 equiv). The resultant mixture was heated to 110 °C for 1 h. Upon completion, the reaction contents were cooled to 23 °C, the reaction mixture was concentrated directly, and then purified by flash column chromatography (silica gel, hexanes) to afford deoxygenated product **4** whose spectral data matched that of natural spiroviolene (see Figures S3 and S4).[3,4]

Figure S3. ^{1}H NMR comparison of the Double Barton product (top) to synthetic **4** (bottom).

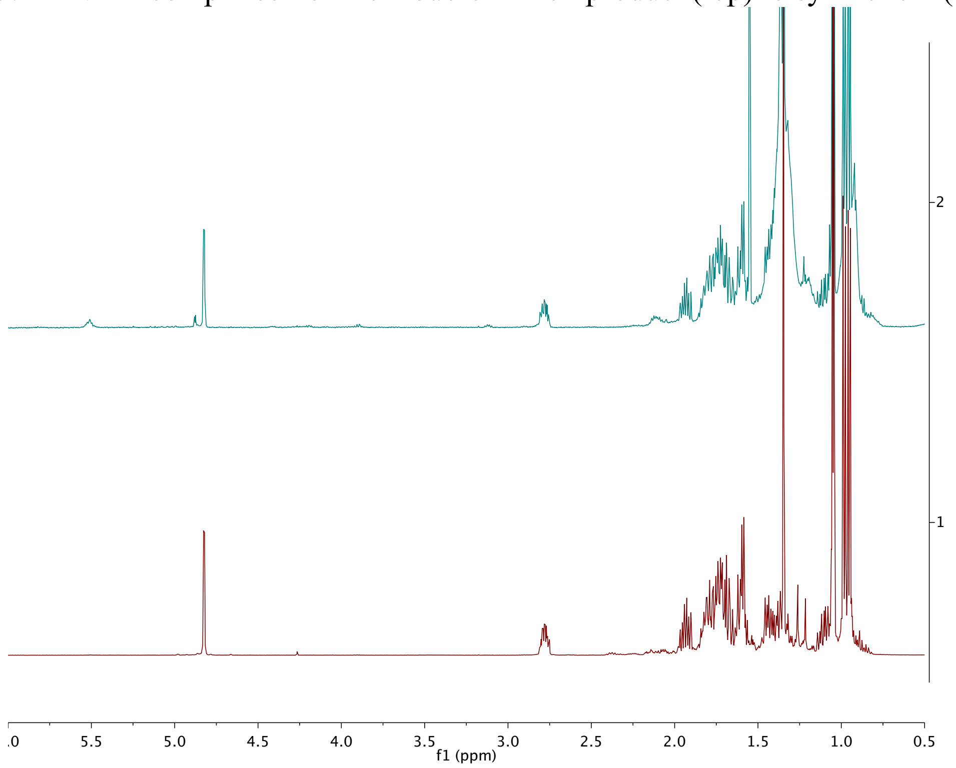

Figure S4. ^{13}C NMR comparison of the Double Barton product (top) to synthetic **4** (bottom).

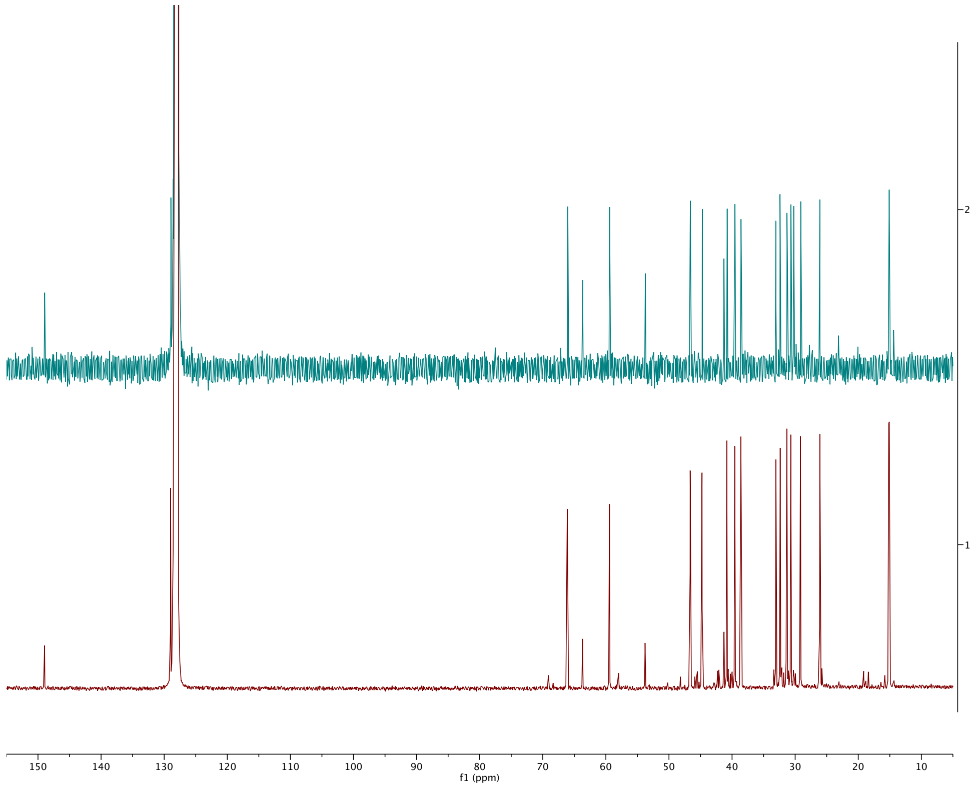

D. Computational General Information

DFT optimizations were performed through Gaussian 09 on the Midway2 Cluster at the University of Chicago's Research Computing Center. The ground state geometry optimizations and free energies (kcal/mol) of BH_3 and **26** were determined separately using the B3LYP/cc-pvDZ level of theory in the gas phase before being combined for subsequent transition state calculations using the B3LYP/cc-pvDZ level of theory in the gas phase. All ground state structures display no imaginary frequencies. Transition state structures display one negative frequency corresponding to the bond forming event. The complex and product were derived from IRC calculations using the B3LYP/cc-pvDZ level of theory in the gas phase. Further single point energy calculations were then performed at the B3LYP/aug-cc-pvDZ level of theory in the gas phase.

E. Potential Energy Surface and Minimized Structures

Table S1. Computed zero-point corrected energies for both the alpha- and beta-face hydroboration of **26** calculated at the B3LYP/aug-cc-pvDZ level of theory in the gas phase.

structure	E_0, Hartree	ZPE (HO), Hartree	ΔH (0 K), Hartree	rel. ΔH (0 K), kcal/mol
**BH_3**	-26.6122	0.0260	-26.5862	--
**26**	-894.7778	0.4830	-894.2948	--
**Combined**	-921.3900	0.5091	-920.8810	8.60
**alpha pathway**				
**α-complex**	-921.4101	0.5154	-920.8947	0.00
**α-TS**	-921.4083	0.5151	-920.8932	0.95
**α -product**	-921.4232	0.5172	-920.9060	-7.08
**beta pathway**				
**β-complex**	-921.3971	0.5140	-920.8831	7.29
**β-TS**	-921.3955	0.5150	-920.8804	8.98
**β-product**	-921.4314	0.5181	-920.9134	-11.73

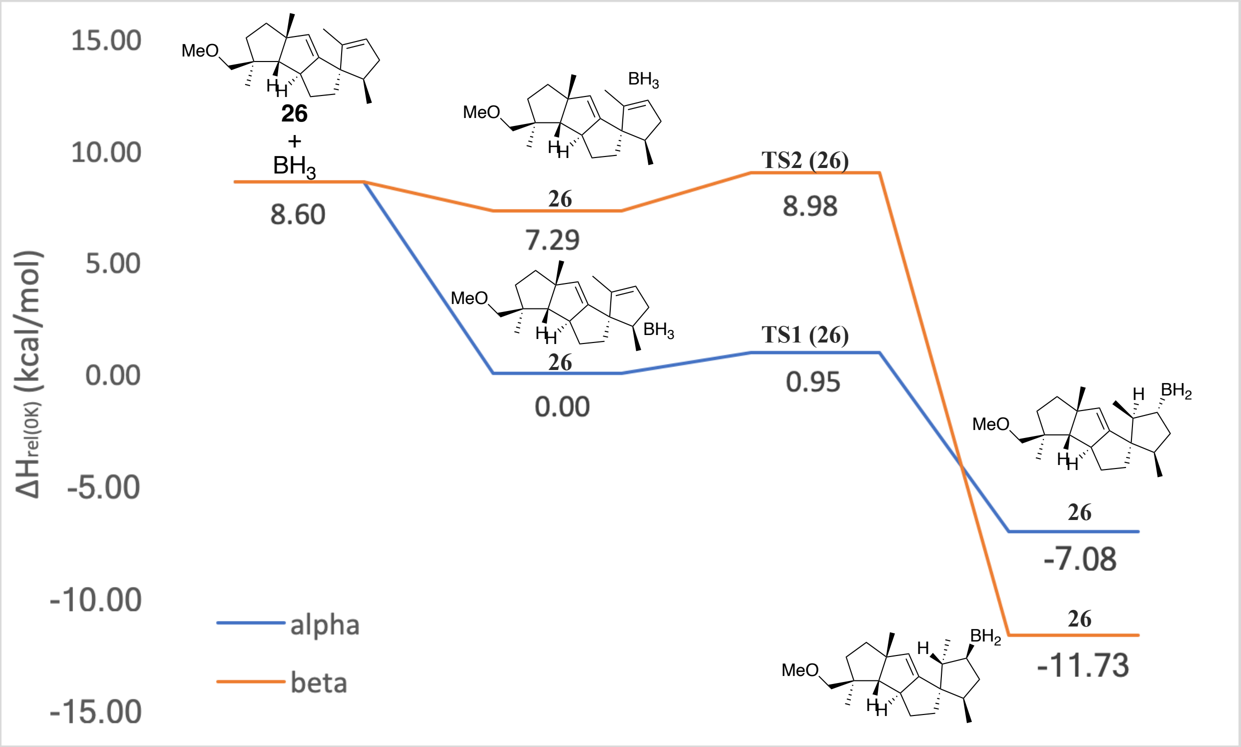

Figure S5. Computational potential energy surface (B3LYP/aug-cc-pvDZ//B3LYP/cc-pvDZ) of both the α- and β-face hydroboration of **26** indicates kinetic preference for the α-face hydroboration via transition state TS1. Values are relative, zero-point energy corrected total energies [$\Delta H_{rel(0K)}$(kcal/mol)].

26 – BH_3

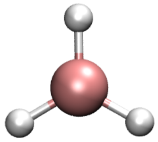

E(RB3LYP) = -26.6122185835

Zero-point correction= 0.026011 (Hartree/Particle)

0 1

B	0.00000000	0.00000000	0.00000000
H	0.00000000	1.20421200	0.00000000
H	1.04287800	-0.60210600	0.00000000
H	-1.04287800	-0.60210600	0.00000000

26 – hydroboration precursor

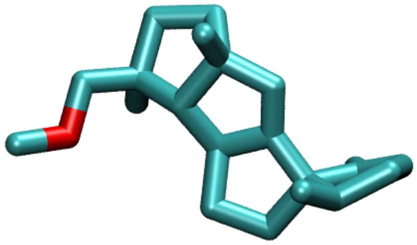

E(RB3LYP) = -894.777830243

Zero-point correction= 0.483041 (Hartree/Particle)

0 1

C	2.73547800	2.02701900	0.61398400
C	2.62574800	0.46914100	0.60587900
C	1.30372200	0.16938200	-0.19715600
C	0.71904500	1.56024000	-0.66475100
C	1.30984200	2.54518800	0.37749800
C	0.11997500	-0.51759100	0.52262900
C	-1.10501600	0.20455000	0.00325900
C	-0.78784700	1.35744900	-0.59893800
C	3.83264900	-0.12292700	-0.13399600
C	-0.25278000	-1.99145500	0.30793000
C	-1.75138100	-1.99984600	0.69345600
C	-2.36254200	-0.64195400	0.18888800
C	-3.25458100	-0.82723300	-1.09701700
C	-4.31468900	0.29381500	-1.01587800
C	-4.39091200	0.56851300	0.46366200
C	-3.35273600	0.05159100	1.13894900
H	1.54818500	-0.44062500	-1.08104700
H	0.17460200	-0.35332300	1.61417500
C	1.15071600	1.95001400	-2.08989300
C	2.62648300	-0.06870900	2.04780100
C	-3.09164000	0.20350200	2.60744600
C	-2.55046700	-0.94884600	-2.44577400
O	3.73195200	-1.53567400	-0.14337900
C	4.80080800	-2.16127200	-0.81593900
H	3.39723900	2.36676600	-0.20140600
H	3.17635900	2.40339400	1.55080900
H	0.72547400	2.48347000	1.31093400
H	1.28319500	3.59593400	0.04542900
H	-1.49045800	2.06474700	-1.04748600

H	3.86970200	0.26145100	-1.17555900
H	4.77591700	0.19042800	0.36394200
H	0.34282500	-2.69848700	0.90584400
H	-0.12059200	-2.26022700	-0.75453300
H	-1.83374100	-2.06138400	1.79097900
H	-2.29495200	-2.86695000	0.28782200
H	-3.79779900	-1.77167500	-0.91393600
H	-5.27780600	-0.01645900	-1.45717900
H	-3.99715400	1.19830300	-1.57134200
H	-5.18368700	1.16538600	0.92285800
H	2.24399200	2.05652400	-2.17844000
H	0.70011600	2.91297700	-2.38510400
H	0.82938300	1.18963200	-2.81990800
H	3.60038700	0.13710600	2.52336000
H	2.46599100	-1.15587400	2.07363800
H	1.85395600	0.41493300	2.66548200
H	-2.10726100	0.66911900	2.79123400
H	-3.08415700	-0.76865400	3.13015100
H	-3.85844600	0.83292300	3.08378200
H	-1.84477800	-1.79542100	-2.46293100
H	-1.98589700	-0.03840600	-2.69772200
H	-3.28986800	-1.12160700	-3.24588400
H	4.62795100	-3.24659400	-0.77407300
H	4.85912100	-1.84930100	-1.87894600
H	5.77731600	-1.93614200	-0.34013900

X26 - α-complex

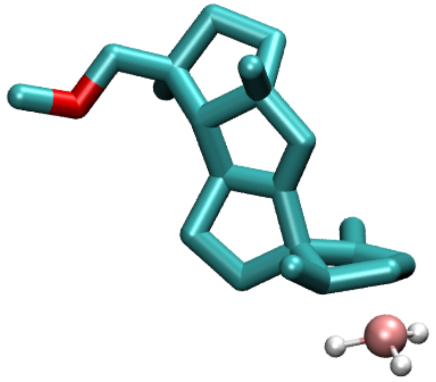

E(RB3LYP) = -921.410125570

Zero-point correction= 0.515413 (Hartree/Particle)

0 1

C	-2.97731000	1.83050500	-1.01339300
C	-2.82253000	0.30501800	-0.72426800
C	-1.54432100	0.20456400	0.19278200
C	-1.00183700	1.67443000	0.39463800
C	-1.57580700	2.42921600	-0.83240100
C	-0.31862400	-0.59766800	-0.30310700
C	0.87107500	0.25932800	0.07187100
C	0.50977400	1.50363200	0.40990900
C	-4.05265300	-0.19909900	0.04135200
C	0.07279900	-1.96414800	0.27782700
C	1.58429100	-2.03278500	-0.04556300
C	2.15135300	-0.57705000	0.10255700
C	3.04912500	-0.38406400	1.37730600
C	3.99437000	0.77862100	1.01352400
C	4.11664600	0.66324100	-0.48978100
C	3.08175300	-0.07846400	-1.01947900
H	-1.83709800	-0.21294800	1.16868700
H	-0.33277100	-0.70503900	-1.40250000
C	-1.48355900	2.32026400	1.70632900
C	-2.71909100	-0.47398800	-2.04756200
C	2.65251100	-0.08045600	-2.45816100
C	2.34592400	-0.24270200	2.72450600
O	-3.90013500	-1.57978700	0.31584900
C	-4.98756500	-2.12574200	1.02811500
H	-3.67477400	2.28679500	-0.28975200
H	-3.39601700	2.02039700	-2.01442100
H	-0.95628200	2.21470800	-1.71962200
H	-1.58809300	3.52347500	-0.70117000
H	1.17866200	2.31065500	0.72015100
H	-4.16809200	0.36689900	0.99024500
H	-4.97305300	-0.02620200	-0.55731700
H	-0.48958200	-2.80978700	-0.14623000
H	-0.09378100	-1.96713600	1.36895200
H	1.71908400	-2.38000900	-1.08205700
H	2.13067300	-2.74151700	0.59355900
H	3.67297400	-1.29168100	1.41682600

H	4.96024100	0.71823500	1.53696800
H	3.54420600	1.75720100	1.26557300
H	4.71297900	1.35852400	-1.08082800
H	-2.58146000	2.40651300	1.74467800
H	-1.06787800	3.33601500	1.81980400
H	-1.16493700	1.72578300	2.57768700
H	-3.66373100	-0.37942600	-2.60897500
H	-2.54106100	-1.54459300	-1.87352700
H	-1.91653600	-0.08493200	-2.69345400
H	2.43522000	-1.09601600	-2.82038800
H	3.42204500	0.35708700	-3.10673000
H	1.73055400	0.51676300	-2.55509300
H	1.71333800	-1.11784800	2.94525100
H	1.70693200	0.65199700	2.76740400
H	3.09162900	-0.16636100	3.53308200
H	-4.77278100	-3.19156600	1.19303700
H	-5.12587100	-1.63240100	2.01194500
H	-5.93800900	-2.03551300	0.46359700
B	4.90836600	-1.00895700	-1.03982200
H	4.20986400	-2.00459800	-1.03293700
H	5.65153700	-1.00948300	-0.08108200
H	5.38317600	-0.74683800	-2.12105700

26 - α-TS

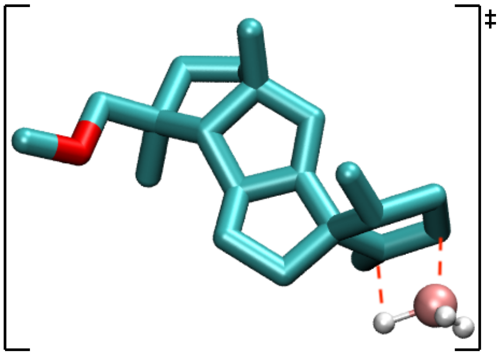

E(RB3LYP) = -921.408270252

Zero-point correction= 0.515109 (Hartree/Particle)

0 1

C	-2.95026200	1.91681500	-0.85833300
C	-2.80829100	0.37239300	-0.68940700

C	-1.53118800	0.19181700	0.21728500
C	-0.97031400	1.63662300	0.52311200
C	-1.54114600	2.48567900	-0.64237300
C	-0.31595800	-0.58839600	-0.33581500
C	0.88516800	0.22723500	0.09074500
C	0.53930300	1.44780800	0.51906100
C	-4.04319600	-0.18129400	0.03296400
C	0.05780100	-1.99462400	0.15554700
C	1.56749300	-2.06235800	-0.17353700
C	2.15375000	-0.62564000	0.05729700
C	3.06524500	-0.51334800	1.33078100
C	3.99853100	0.67847500	1.03756100
C	4.14783900	0.65471200	-0.47721200
C	3.07747700	-0.07835500	-1.05266400
H	-1.82881600	-0.29019300	1.16141600
H	-0.33391600	-0.62358700	-1.43961200
C	-1.43951400	2.19112800	1.88037400
C	-2.70750500	-0.30146900	-2.06913700
C	2.55936000	0.17136400	-2.44551700
C	2.37251800	-0.46715600	2.69060100
O	-3.90219900	-1.58101000	0.19521300
C	-4.99421100	-2.17351800	0.86185300
H	-3.63728300	2.32215400	-0.09542400
H	-3.37456800	2.18736800	-1.83821200
H	-0.92924100	2.32984900	-1.54697400
H	-1.54047500	3.56741100	-0.43114100
H	1.21906300	2.22260000	0.88263800
H	-4.15548200	0.30704200	1.02449700
H	-4.96135500	0.04680200	-0.55050500
H	-0.51676700	-2.80372300	-0.32035100
H	-0.10691200	-2.06481400	1.24447200
H	1.69503800	-2.34808200	-1.23080800
H	2.10571300	-2.81504800	0.42129700
H	3.69093400	-1.41968500	1.30739500
H	4.95831300	0.58951700	1.56972600
H	3.53434800	1.63024000	1.35556300
H	4.57687800	1.50965900	-1.00227900
H	-2.53595900	2.29085600	1.92796100
H	-1.00803000	3.18932700	2.06658200
H	-1.12830300	1.52971800	2.70487400

H	-3.65104100	-0.15999100	-2.62245400
H	-2.53356200	-1.38286500	-1.97811300
H	-1.90246400	0.13399500	-2.68140100
H	2.06016300	-0.71263000	-2.86986800
H	3.37582700	0.46391200	-3.11896300
H	1.82784200	0.99425400	-2.40801800
H	1.74883000	-1.36017600	2.86048000
H	1.72818500	0.41798600	2.79920700
H	3.12615700	-0.43821400	3.49484100
H	-4.78900900	-3.25115500	0.93875700
H	-5.12744500	-1.76100000	1.88286800
H	-5.94404900	-2.02876900	0.30778100
B	4.82985200	-0.80826700	-1.10702400
H	3.90657700	-1.57464200	-1.45671700
H	5.34880800	-1.38948000	-0.18345800
H	5.43293400	-0.54658100	-2.12175100

26 - α-product

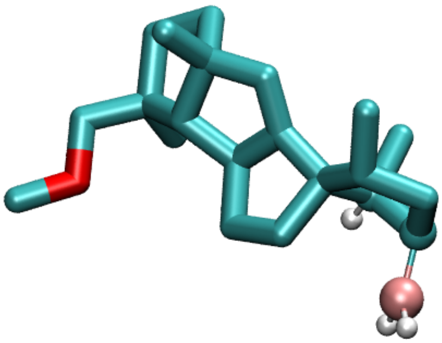

E(RB3LYP) = -921.423190537

Zero-point correction= 0.517197 (Hartree/Particle)

0 1

C	3.16663000	1.55518400	1.17884600
C	2.86715600	0.09448500	0.71695900
C	1.60413700	0.22844900	-0.21594700
C	1.23281700	1.75741800	-0.28203300
C	1.84064600	2.31504800	1.03200200
C	0.28612900	-0.47168900	0.19364700
C	-0.79811700	0.53418900	-0.15800400
C	-0.28551000	1.75560900	-0.36286500

C	4.05699000	-0.44151700	-0.08948400
C	-0.16299400	-1.78669000	-0.49443700
C	-1.59238300	-1.48842900	-1.02387300
C	-2.11542800	-0.19775000	-0.30292900
C	-3.32746300	0.41839100	-1.10347900
C	-4.60079800	0.01901100	-0.30635900
C	-4.14672800	-1.07321000	0.68036600
C	-2.72197400	-0.61112900	1.09039200
H	1.86438600	-0.12601900	-1.22571200
H	0.26030000	-0.62652500	1.28462700
C	1.83209000	2.47355100	-1.50618400
C	2.65776000	-0.81673400	1.93935100
C	-3.30349600	1.91799700	-1.41416400
O	3.77974300	-1.76421600	-0.51457200
C	4.82878300	-2.33573200	-1.26265800
H	3.93244600	2.01095000	0.52751400
H	3.56773800	1.58941400	2.20435900
H	1.17265000	2.07541600	1.87639700
H	1.97044300	3.40958600	1.01968300
H	-0.83969200	2.66070000	-0.61206900
H	4.24465700	0.20888900	-0.97014400
H	4.97800100	-0.42499700	0.53258600
H	-0.15884300	-2.63200800	0.21025300
H	0.51293000	-2.06140400	-1.31855000
H	-2.27752900	-2.35114100	-0.89704700
H	-1.56100000	-1.28988600	-2.10736100
H	-3.35553400	-0.10199700	-2.07570700
H	-5.41051000	-0.32039600	-0.97099100
H	-4.98540400	0.89908200	0.23665700
H	2.93383400	2.44610800	-1.50558800
H	1.52787000	3.53398800	-1.52723600
H	1.48597900	2.00514600	-2.44174800
H	3.59762500	-0.89693500	2.51132500
H	2.36252500	-1.83200500	1.63868100
H	1.89332000	-0.41797100	2.62424400
H	-2.47087700	2.19091800	-2.07878300
H	-3.22882800	2.52789600	-0.49926800
H	-4.23907000	2.20350800	-1.92412200
H	4.51920400	-3.35328000	-1.54234300
H	5.03932000	-1.75928700	-2.18682200

H	5.76850500	-2.39631500	-0.67646200
H	-4.79832000	-1.09780300	1.58426800
B	-4.22649500	-2.55790700	0.19486500
H	-3.70366000	-3.42757400	0.85361000
H	-4.87198100	-2.88406800	-0.77463400
C	-2.73205700	0.51590500	2.13065100
H	-1.71186900	0.85679900	2.36522400
H	-3.29804900	1.39679000	1.78828000
H	-3.19458400	0.16663700	3.06873600
H	-2.14859700	-1.45310800	1.51160500

26 - β-complex

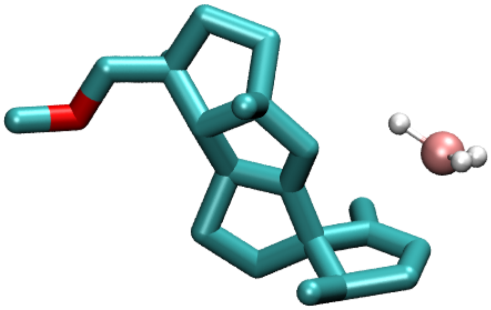

E(RB3LYP) = -921.397063451

Zero-point correction= 0.513998 (Hartree/Particle)

0 1

C	-2.81440000	2.13055200	-0.46156900
C	-2.74956400	0.57861900	-0.58809300
C	-1.50396500	0.16905300	0.28874100
C	-0.83792600	1.50679100	0.80472600
C	-1.37270000	2.57570300	-0.18336000
C	-0.34783000	-0.62318500	-0.36491900
C	0.91231800	0.06193600	0.11830600
C	0.65323900	1.22675900	0.72399900
C	-4.02577100	-0.04382700	-0.00942400
C	-0.06237800	-2.09046200	-0.01746800
C	1.42850500	-2.22311900	-0.40379700
C	2.12567000	-0.85629800	-0.03444800
C	3.04950000	-0.99395300	1.23614700
C	4.18572700	0.02989200	1.02425700
C	4.25611500	0.14471000	-0.48231100

C	3.14586600	-0.36135200	-1.08401400
H	-1.85916500	-0.41516300	1.15179500
H	-0.38654800	-0.55094100	-1.46614500
C	-1.23897300	1.85556600	2.25012700
C	-2.64429700	0.18265300	-2.07196400
C	2.94322300	-0.54169700	-2.55795400
C	2.39207000	-0.95268000	2.61260300
O	-3.94594100	-1.45505400	-0.10995600
C	-5.08251900	-2.11076300	0.40478400
H	-3.46463800	2.41707300	0.38325200
H	-3.24213000	2.60081500	-1.36143000
H	-0.78134300	2.54616500	-1.11330300
H	-1.30687100	3.60197700	0.21260500
H	1.39178100	1.90399800	1.15819800
H	-4.14683500	0.25488700	1.05369200
H	-4.91752100	0.32932400	-0.55806800
H	-0.70306200	-2.81216000	-0.54697600
H	-0.20053200	-2.25012700	1.06576000
H	1.49283700	-2.39984900	-1.48841700
H	1.92768500	-3.07362300	0.08506700
H	3.51493300	-1.98960300	1.12021100
H	5.13713200	-0.31482400	1.46203400
H	3.95773700	1.00384700	1.48974000
H	-2.32524500	2.01357800	2.34942500
H	-0.73727300	2.77987900	2.58336400
H	-0.95448200	1.04825700	2.94427300
H	-3.55613000	0.50008300	-2.60524000
H	-2.55015700	-0.90554800	-2.19487200
H	-1.78949500	0.66799000	-2.56854400
H	1.95205500	-0.17993300	-2.87475300
H	2.99947000	-1.61055400	-2.82469500
H	3.70653600	-0.00500400	-3.13634300
H	1.61703500	-1.72924000	2.71787700
H	1.91967400	0.01937600	2.81609300
H	3.14726200	-1.13313300	3.39587900
H	-4.92447300	-3.19225600	0.28241000
H	-5.23383500	-1.89144000	1.48166900
H	-6.00682200	-1.82123500	-0.13582700
B	3.53939800	2.17267100	-1.26142900
H	2.36729800	2.10797300	-1.52452300

H	3.86093100	2.65009900	-0.19969800
H	4.32619500	2.20364700	-2.17771200
H	5.16706300	0.41337000	-1.01622300

26 - β-TS

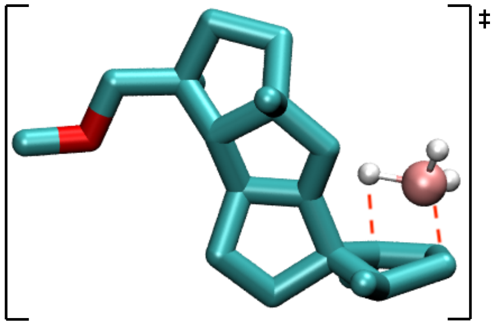

E(RB3LYP) = -921.395467019

Zero-point correction= 0.515037 (Hartree/Particle)

0 1

C	2.95198100	1.74487700	1.17762600
C	2.73152500	0.27358100	0.73266700
C	1.55142700	0.38155600	-0.30660000
C	0.90758400	1.81858500	-0.12973800
C	1.55154600	2.37125900	1.16930200
C	0.37584300	-0.61640400	-0.20542500
C	-0.86798900	0.23901800	-0.12372300
C	-0.58420700	1.54555200	-0.06380000
C	3.99233200	-0.27437000	0.05628800
C	0.01148400	-1.55452000	-1.36503500
C	-1.47272000	-1.87914900	-1.07038000
C	-2.10977900	-0.60864300	-0.37683400
C	-3.22767400	0.04499600	-1.27023500
C	-4.16691800	0.76433300	-0.28072700
C	-4.09141600	-0.11700400	0.96420200
C	-2.94987300	-0.94720100	0.90922100
H	1.98257100	0.30643300	-1.31716800
H	0.45063100	-1.23685800	0.70389700
C	1.21928200	2.75074200	-1.31711700
C	2.41992400	-0.59547500	1.96534500
C	-2.90077900	-2.29506900	1.58330100

C	-2.78446700	0.86177200	-2.48049500
O	3.74260400	-1.59298300	-0.40237500
C	4.85784500	-2.17497600	-1.03837900
H	3.59757800	2.26646900	0.44896500
H	3.45267200	1.81556700	2.15666300
H	0.97858100	2.03198200	2.04687400
H	1.57431500	3.47244900	1.20036100
H	-1.31184600	2.35893800	-0.02801000
H	4.27898600	0.37532700	-0.79807700
H	4.84476700	-0.27322300	0.76941600
H	0.63950200	-2.45671300	-1.42168400
H	0.10869500	-1.01471800	-2.32278600
H	-1.51846300	-2.74989900	-0.40182900
H	-2.03147400	-2.15343900	-1.97833400
H	-3.80165200	-0.82084600	-1.65178200
H	-5.19489200	0.82387900	-0.67254000
H	-3.83695900	1.79544600	-0.08027200
H	2.30132400	2.94338200	-1.40859100
H	0.71687900	3.72536000	-1.19474100
H	0.87498800	2.31048700	-2.26697700
H	3.25681900	-0.53444700	2.68099100
H	2.29836300	-1.65229400	1.68989200
H	1.51036900	-0.26363000	2.48921900
H	-1.87623300	-2.60730100	1.83330300
H	-3.33562100	-3.05467100	0.91238300
H	-3.49321900	-2.28064100	2.50810000
H	-2.12050700	0.28005100	-3.14096200
H	-2.24818300	1.77665800	-2.19148400
H	-3.66341200	1.15870700	-3.07667700
H	4.56635100	-3.18593100	-1.35857600
H	5.17425600	-1.59513200	-1.92962700
H	5.72851300	-2.25332400	-0.35556600
B	-3.02102000	0.40774900	2.25349900
H	-1.91597300	-0.14902700	2.17569200
H	-2.78519900	1.56716800	2.01906500
H	-3.52045800	0.04547100	3.29435500
H	-5.00750500	-0.46731900	1.44122500

26 - β-product

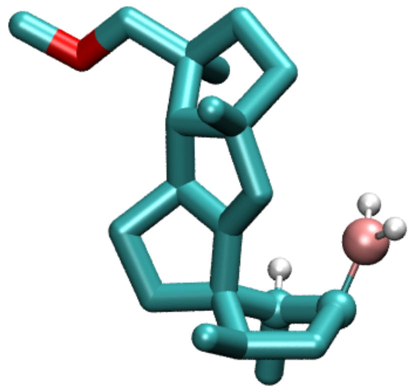

E(RB3LYP) = -921.431436740

Zero-point correction= 0.518052 (Hartree/Particle)

0 1

C	-2.87493800	1.96384800	-0.72390700
C	-2.70580200	0.42131500	-0.64050800
C	-1.51756700	0.23594800	0.37876100
C	-0.84779900	1.65911800	0.57573600
C	-1.45748700	2.52607900	-0.55622100
C	-0.36015400	-0.72281700	0.01470800
C	0.90094100	0.09043200	0.18482500
C	0.64357000	1.37812000	0.47745400
C	-3.98260200	-0.22383500	-0.09139500
C	-0.02331800	-1.94524900	0.88962800
C	1.47400300	-2.20217800	0.57036700
C	2.11034000	-0.82479800	0.16583500
C	3.31535400	-0.34734600	1.02638800
C	3.97103700	0.77840000	0.17588900
C	3.35364200	0.64929100	-1.25238400
C	2.72220200	-0.76612100	-1.27923400
H	-1.94298900	-0.09136900	1.34017000
H	-0.43329600	-1.07468800	-1.02709800
C	-1.17100300	2.27348100	1.95180600
C	-2.43426400	-0.14629500	-2.04593100
C	3.72579500	-1.87837600	-1.61192300
C	3.02936100	0.02600200	2.48127600
O	-3.78240400	-1.62119100	0.03803000
C	-4.91356200	-2.29645500	0.53956700
H	-3.51776600	2.31856800	0.10110500

H	-3.35654500	2.28114900	-1.66265800
H	-0.88349200	2.38918300	-1.48612700
H	-1.44735800	3.60289300	-0.32282800
H	1.38047500	2.13350400	0.75564900
H	-4.23403100	0.21604900	0.89723700
H	-4.84052000	-0.02131600	-0.76835400
H	-0.66163600	-2.81869500	0.68739800
H	-0.14951200	-1.67912000	1.95363600
H	1.55491200	-2.91226800	-0.26755200
H	2.00327100	-2.66170400	1.41958800
H	4.01196700	-1.20292600	1.05229300
H	5.06712500	0.66793300	0.16969300
H	3.76475500	1.77183800	0.60643500
H	4.14295100	0.72485300	-2.03285100
H	-2.25245300	2.45166700	2.07162700
H	-0.65795500	3.24156300	2.08108000
H	-0.84903200	1.60822300	2.76936900
H	-3.27635400	0.10131200	-2.71363800
H	-2.34093000	-1.24096600	-2.02340800
H	-1.52077200	0.27298900	-2.49505700
H	3.27988600	-2.88226500	-1.52553700
H	4.61825000	-1.85307800	-0.96648600
H	4.07335700	-1.76623000	-2.65210700
H	2.57468800	-0.81480700	3.03197100
H	2.34879900	0.88632600	2.56542200
H	3.96613300	0.28751600	3.00102500
H	-4.65940600	-3.36441200	0.60497000
H	-5.19716400	-1.93416200	1.54902800
H	-5.79358000	-2.17808000	-0.12519800
H	1.91028700	-0.81471300	-2.02361900
B	2.35413100	1.78460800	-1.67990600
H	1.57910000	1.59698400	-2.58968000
H	2.48092900	2.91336800	-1.26463700

F. References

1. S. Feuillastre, B. Pelotier and O. Piva, *Eur. J. Org. Chem.* 2014, 1753–1759.
2. P. Hu, H. M. Chi, K. C. DeBacker, X. Gong, J. H. Keim, I. T. Hsu and S. A. Snyder, *Nature* 2019, **569**, 703−707.
3. P. Rabe, J. Rinkel, E. Dolja, T. Schmitz, B. Nubbemeyer, T. H. Luu and J. S. Dickschat, *Angew. Chem. Int. Ed.* 2017, **56**, 2776 –2779.
4. C. H. Kim, H. J. An, W. K. Shin, W. Y. Yu, S. K. Woo, S. K. Jung and E. Lee, *Chem. Asian J.* 2008, **3**, 1523–1534.
5. S. Niu, Z.-W. Fan, C.-L. Xie, Q. Liu, Z.-H. Luo, G. Liu and X.-W. Yang, *J. Nat. Prod.* 2017, **80**, 2174−2177.

G. NMR Spectra

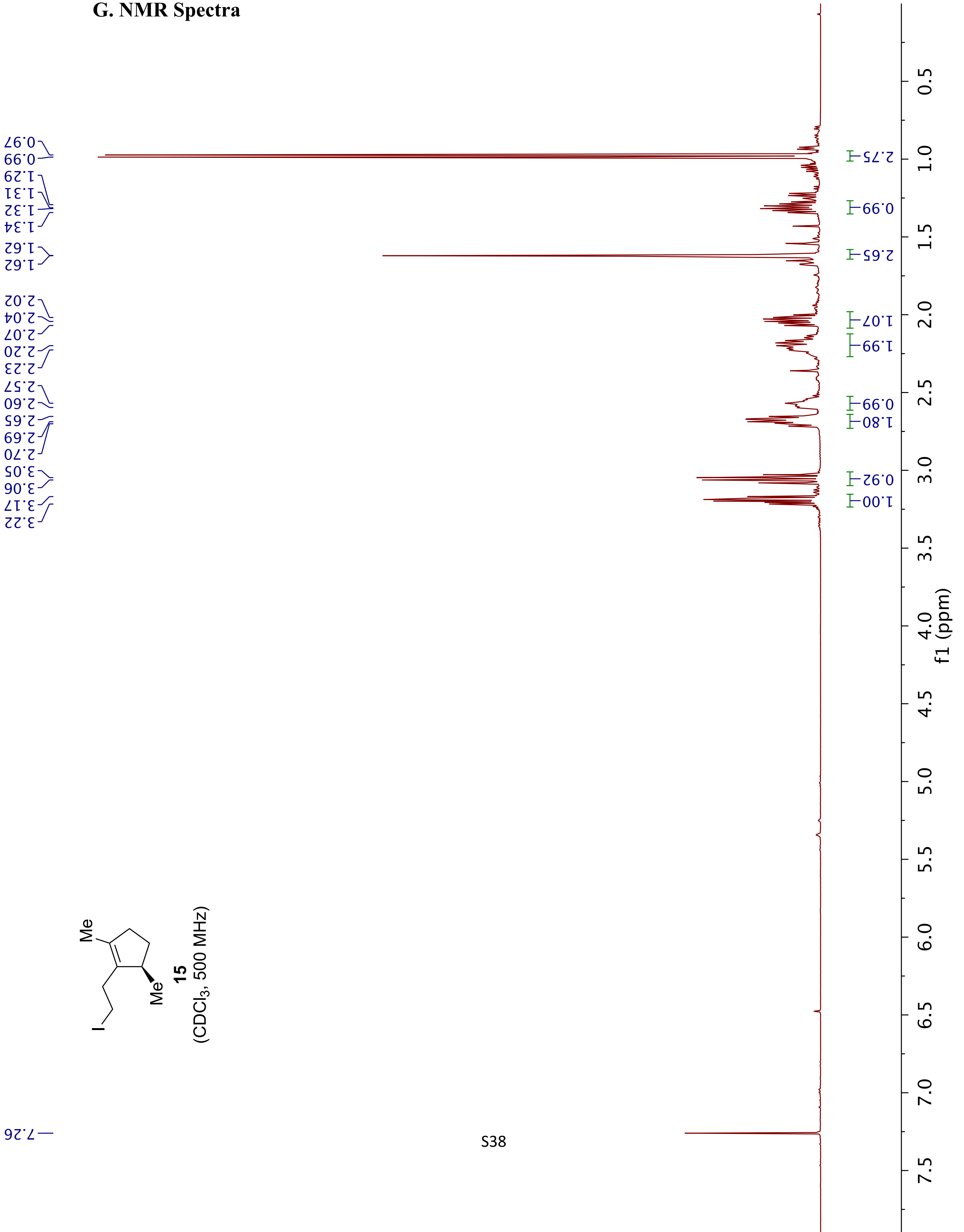

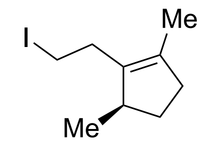

15
($CDCl_3$, 126 MHz)

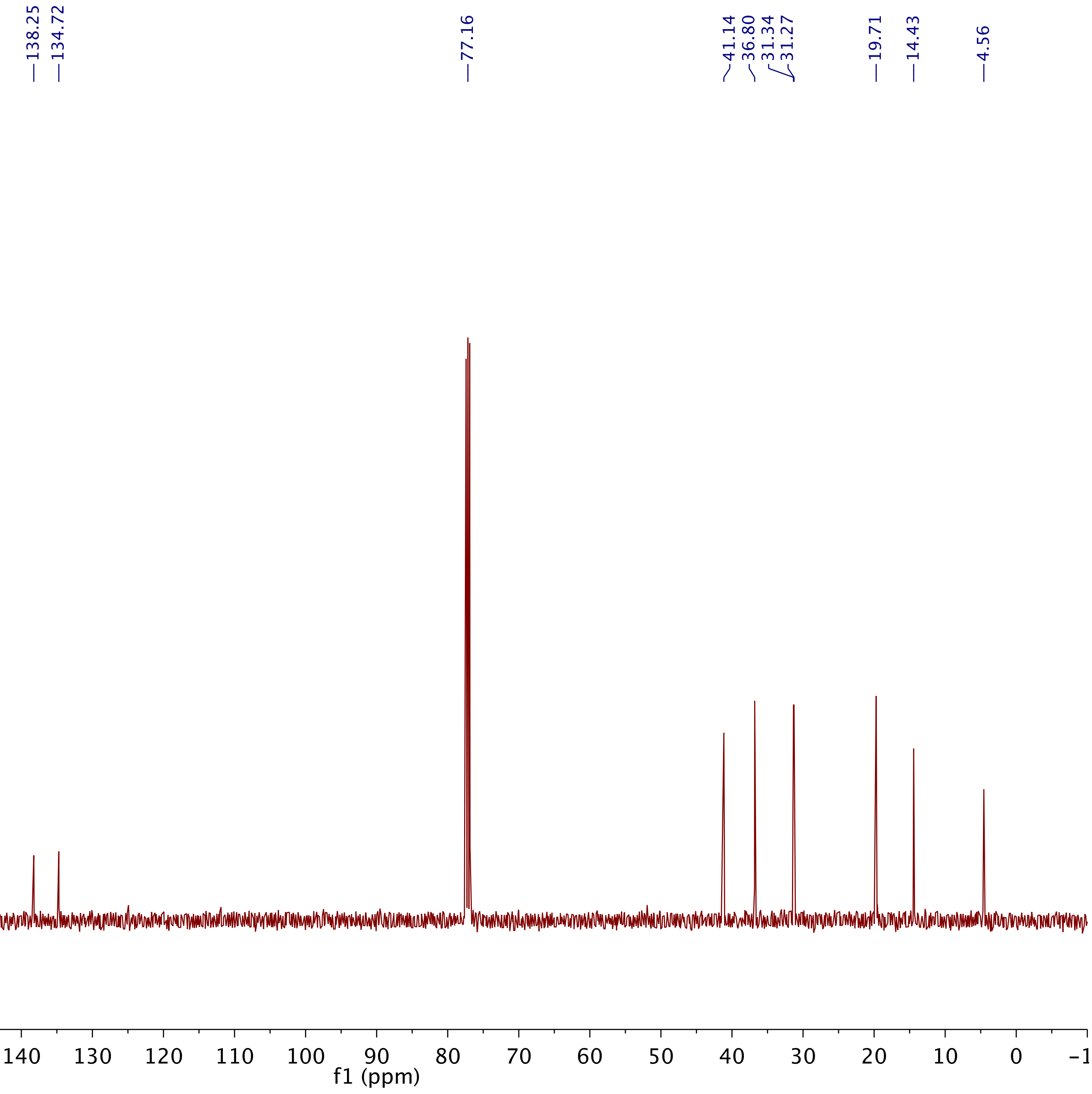

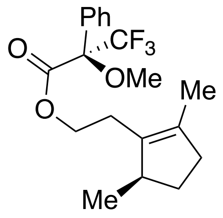

S2
(CDCl$_3$, 500 MHz)

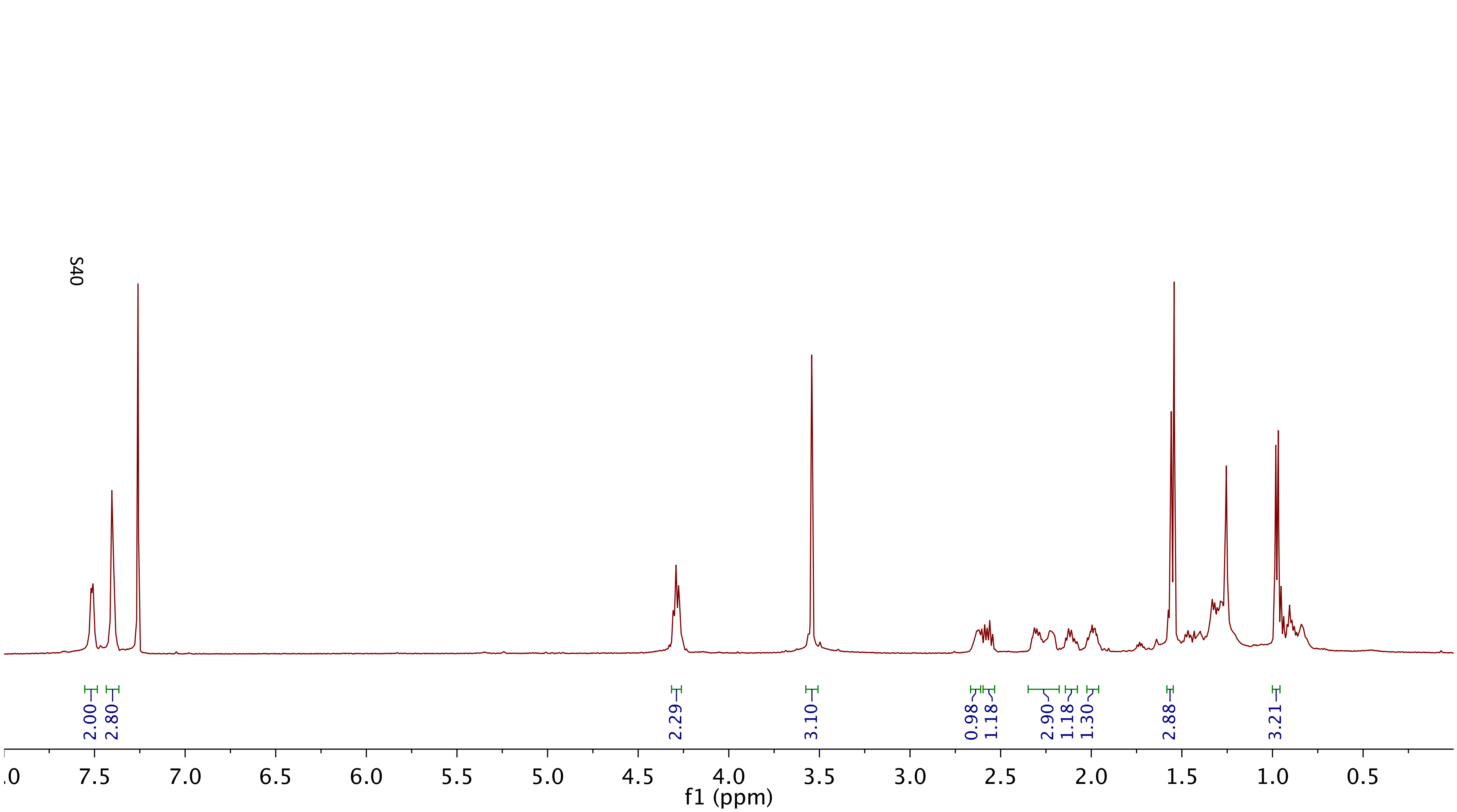

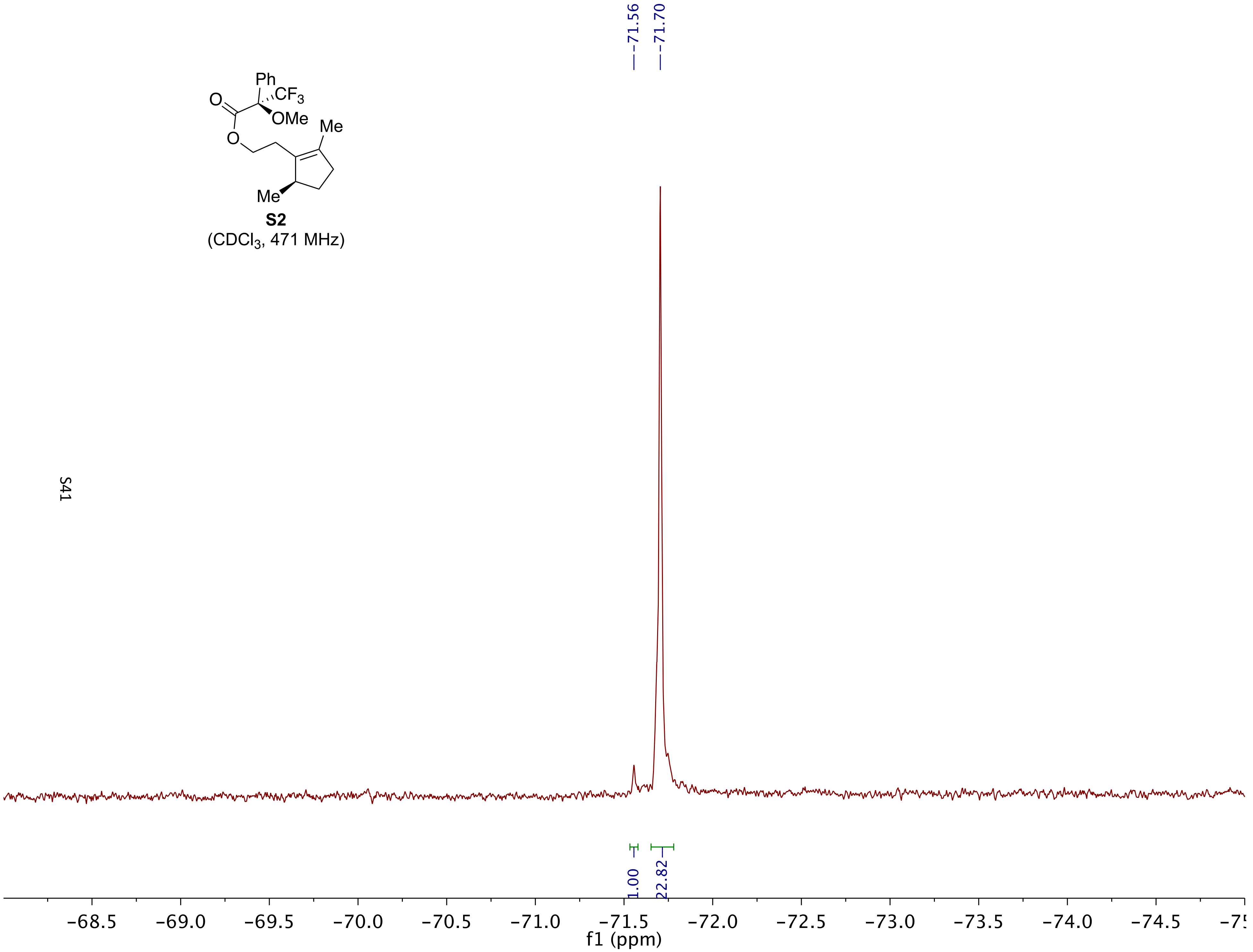

-71.56
-71.70
Ph
CF3
O
OMe
O
Me
Me
S2
(CDCl3, 471 MHz)
1.00
22.82
-68.5
-69.0
-69.5
-70.0
-70.5
-71.0
-71.5
-72.0
-72.5
-73.0
-73.5
-74.0
-74.5
-75
f1 (ppm)

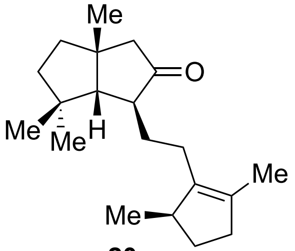

20

($CDCl_3$, 500 MHz)

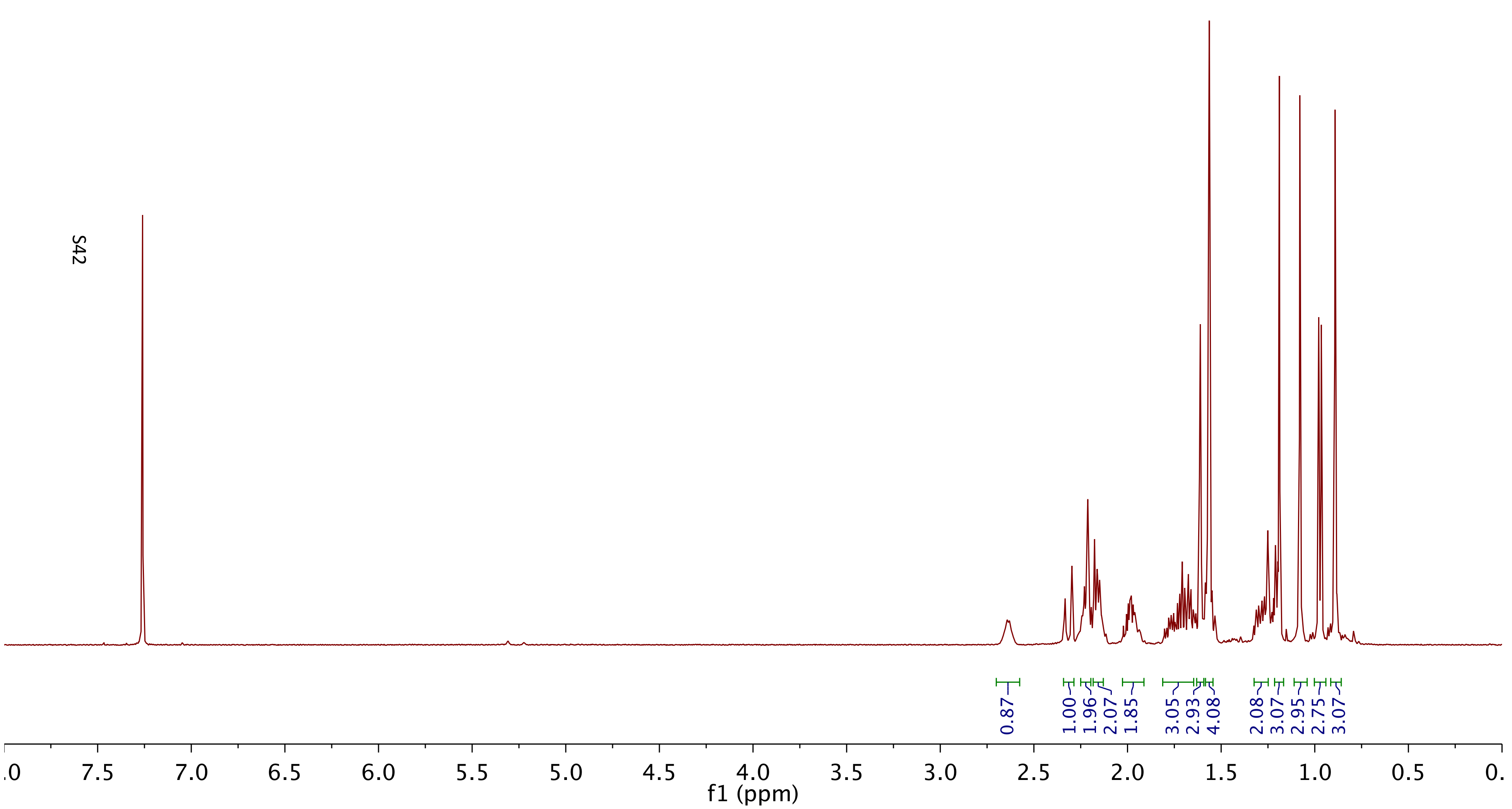

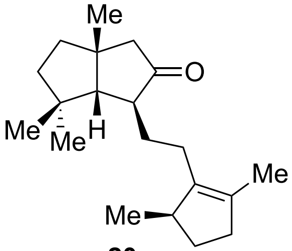

20
($CDCl_3$, 126 MHz)

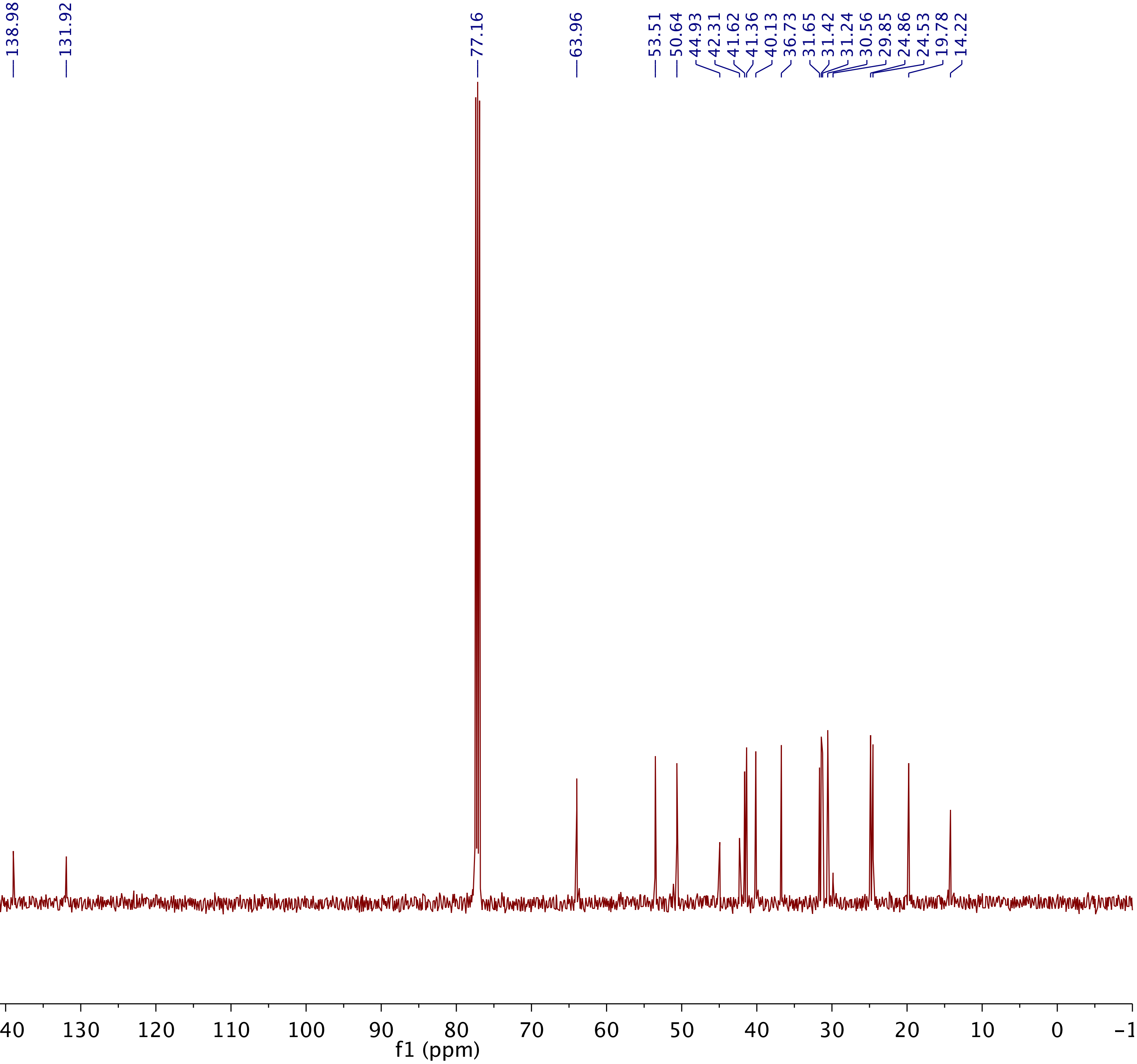

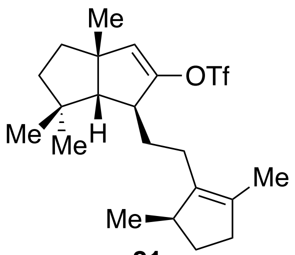

21

(CDCl$_3$, 500 MHz)

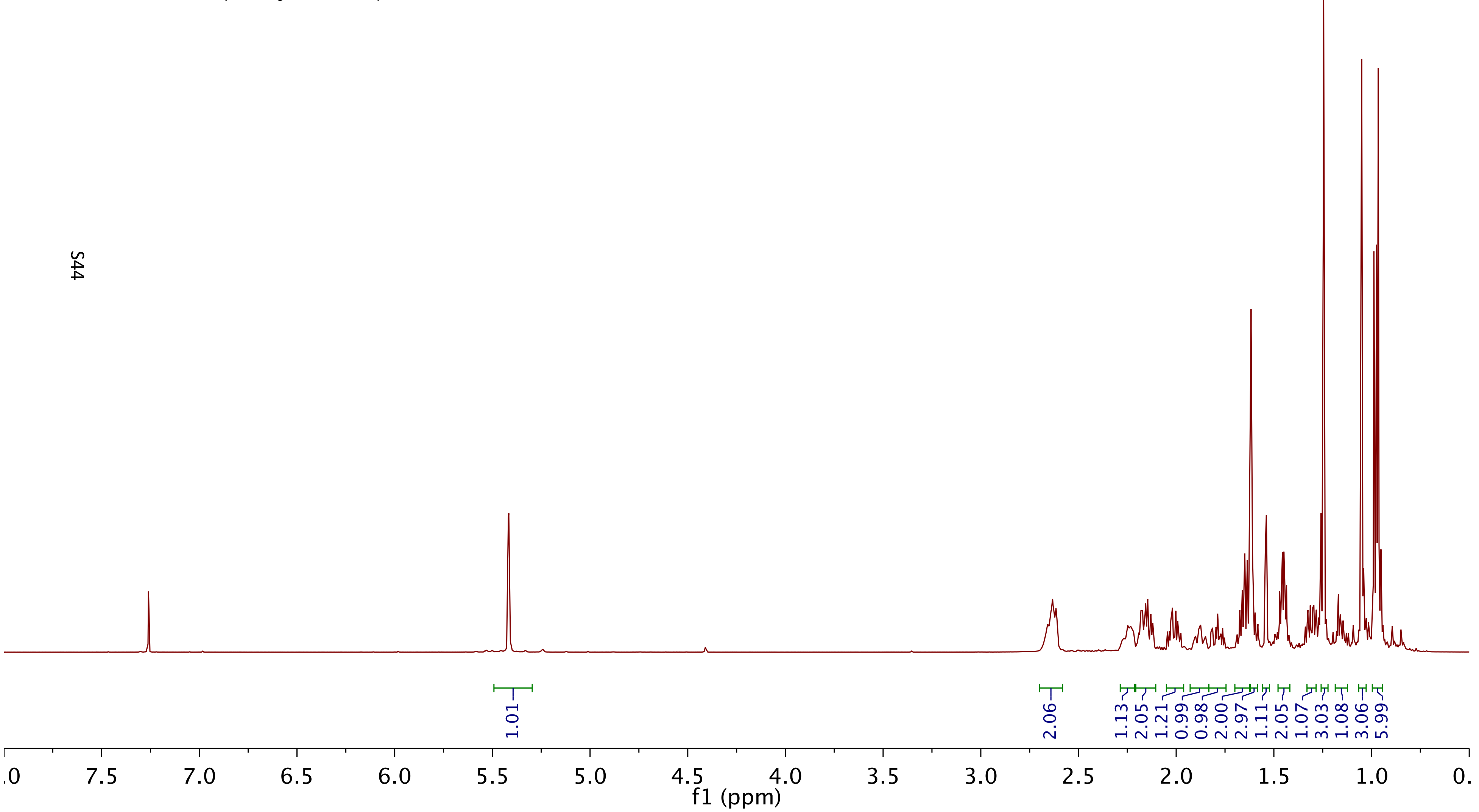

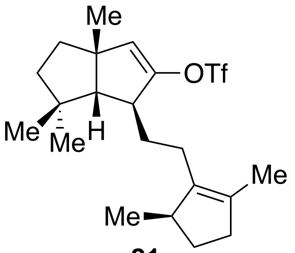

21

($CDCl_3$, 126 MHz)

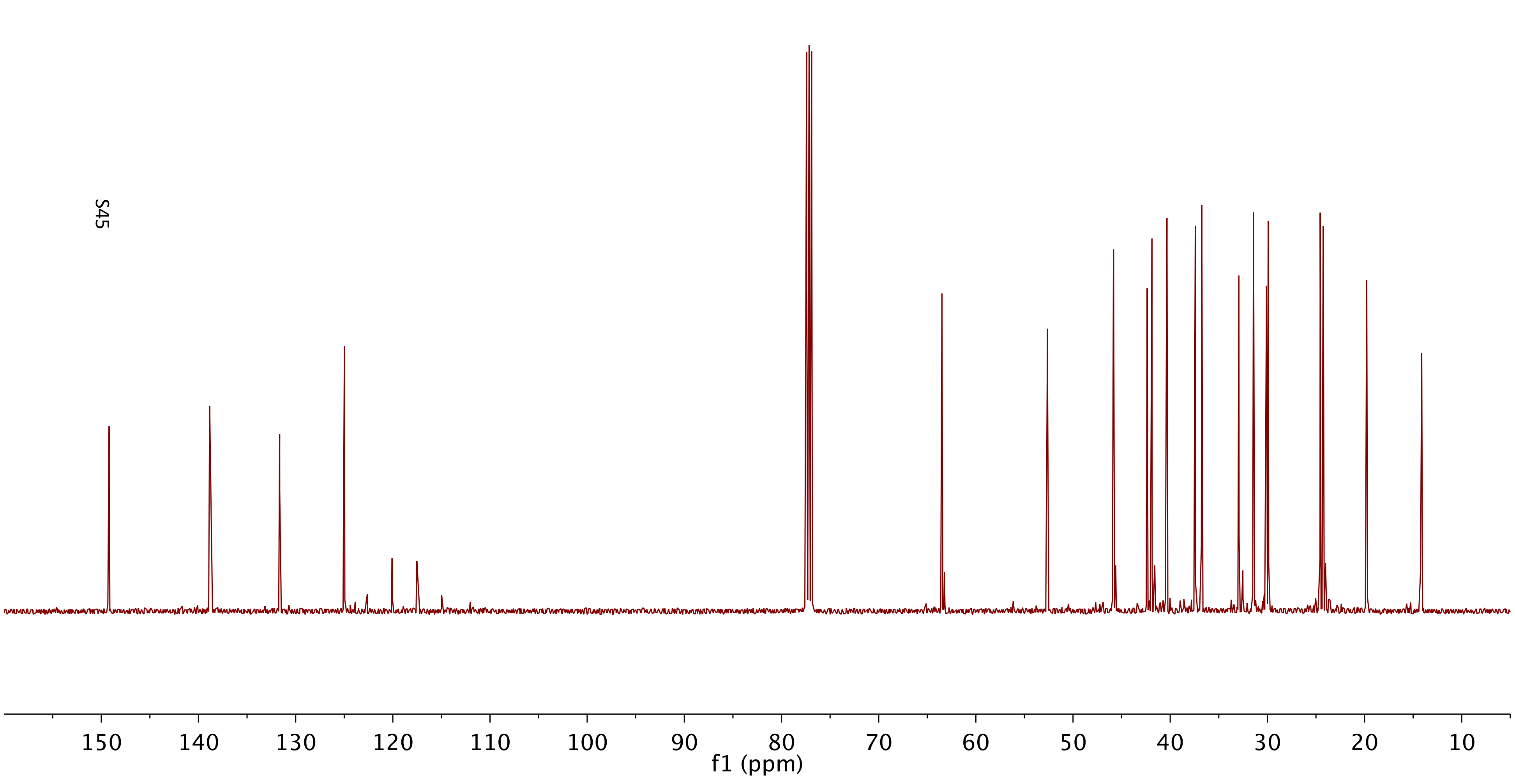

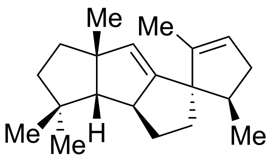

22
($CDCl_3$, 500 MHz)

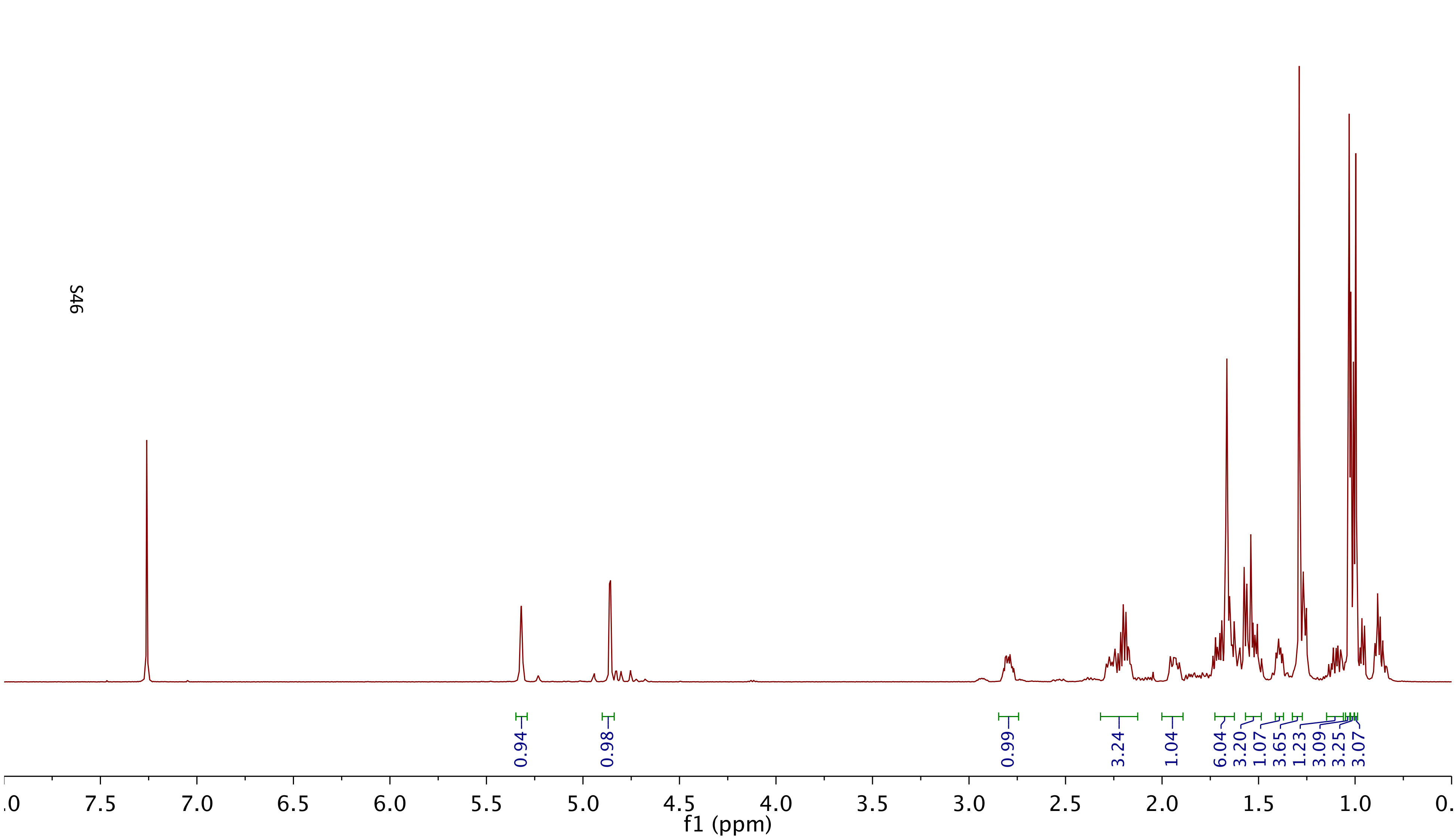

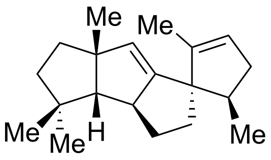

22
($CDCl_3$, 126 MHz)

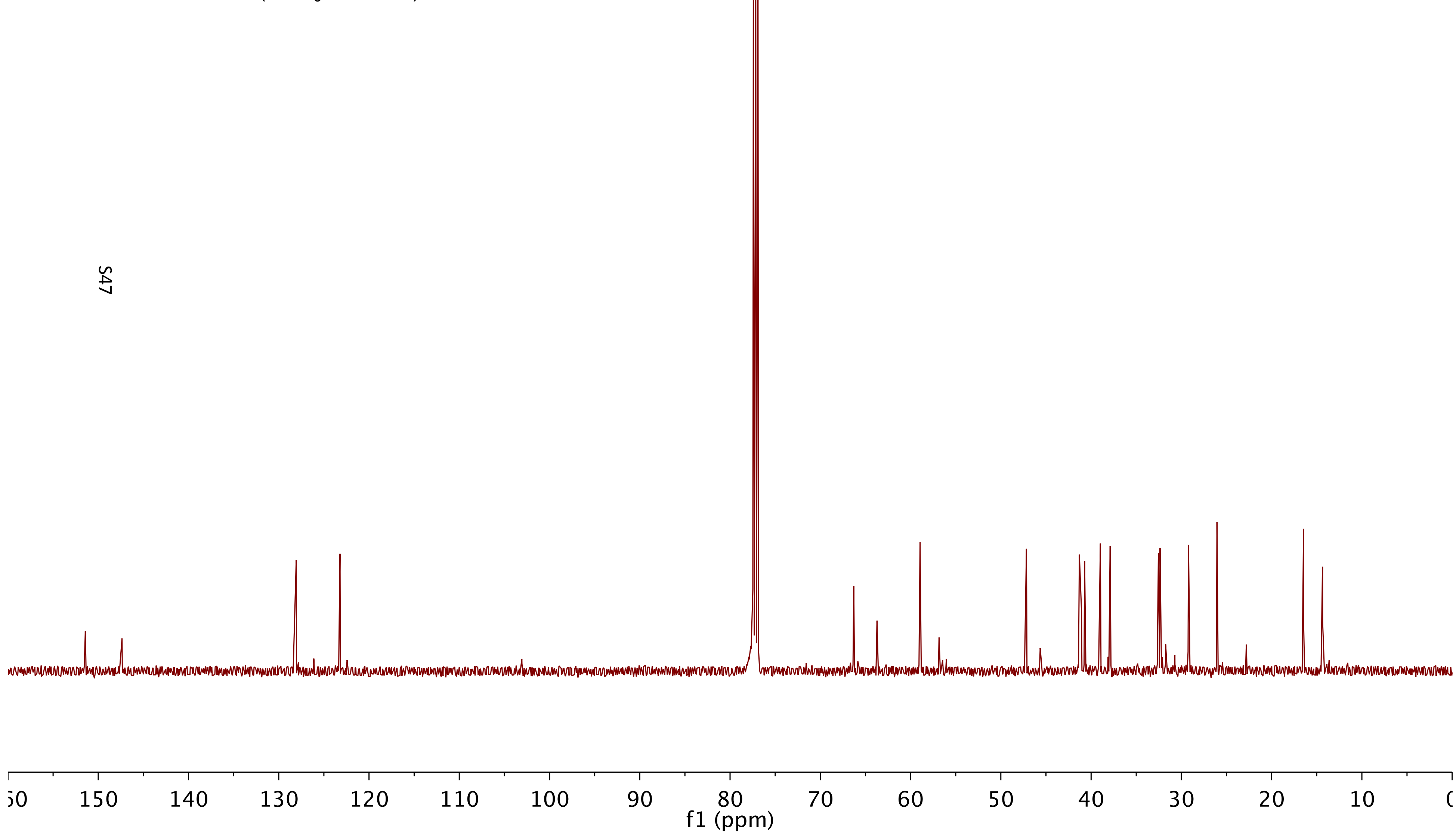

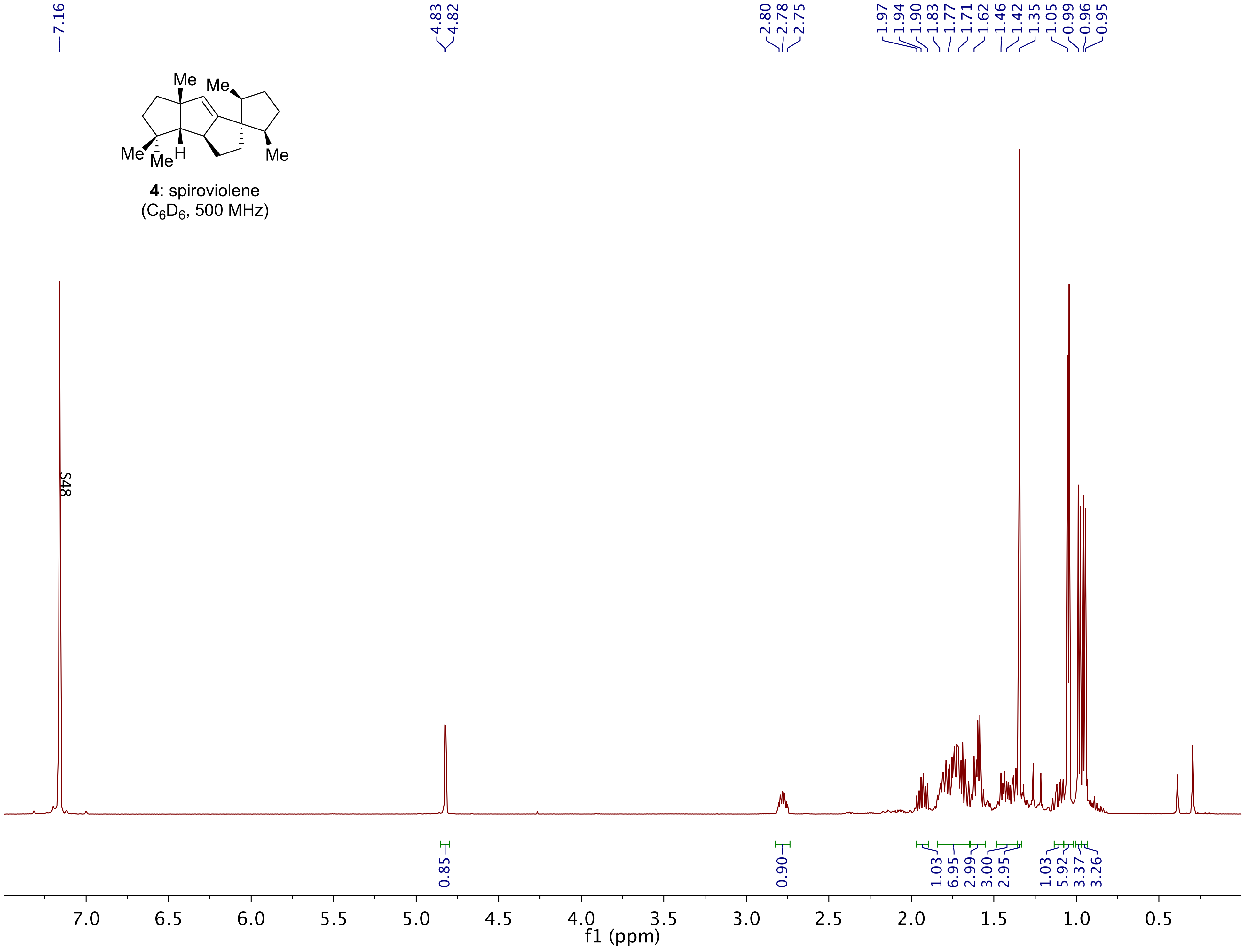

4: spiroviolene
(C_6D_6, 500 MHz)

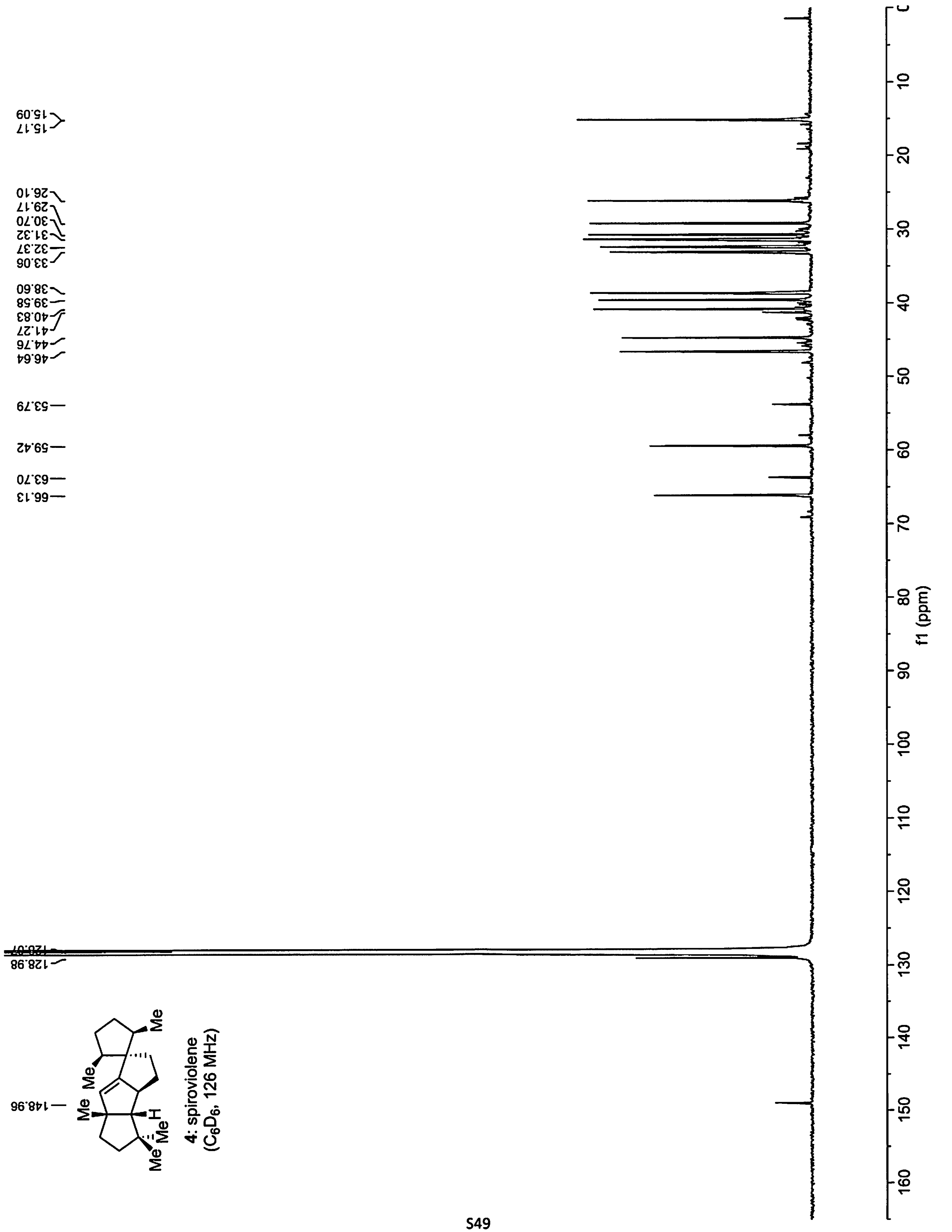
15.09
15.17
26.10
29.17
30.70
31.32
32.37
33.06
38.60
39.58
40.83
41.27
44.76
46.64
53.79
59.42
63.70
66.13
128.98
148.96
4: spiroviolene
(C_6D_6, 126 MHz)
Me
Me
Me
Me
Me
H
f1 (ppm)
0
10
20
30
40
50
60
70
80
90
100
110
120
130
140
150
160

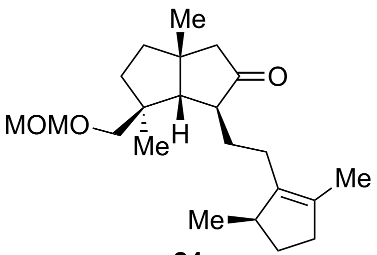

24
($CDCl_3$, 500 MHz)

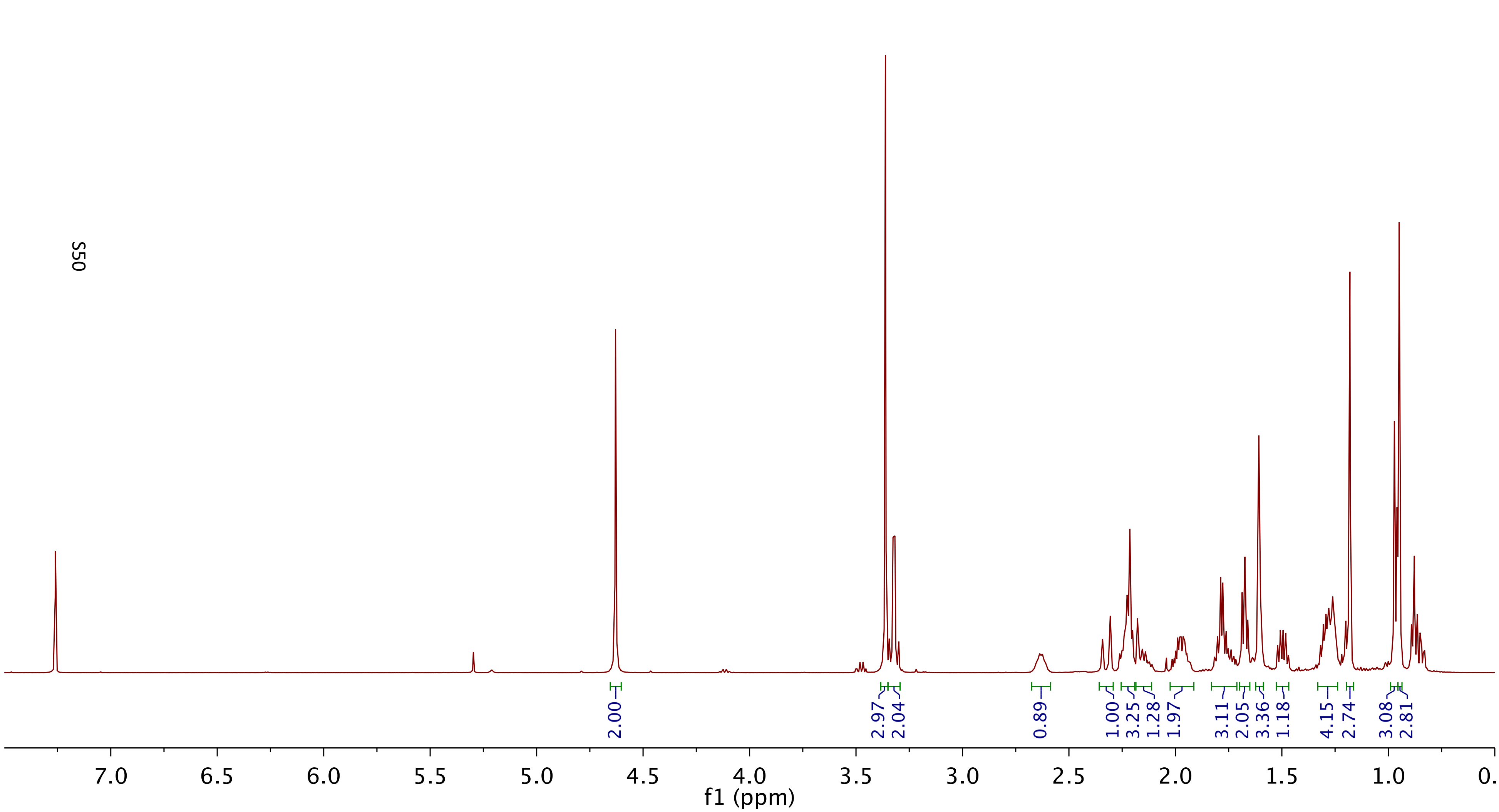

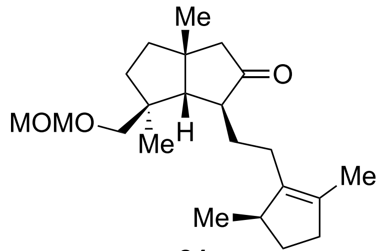

24
($CDCl_3$, 126 MHz)

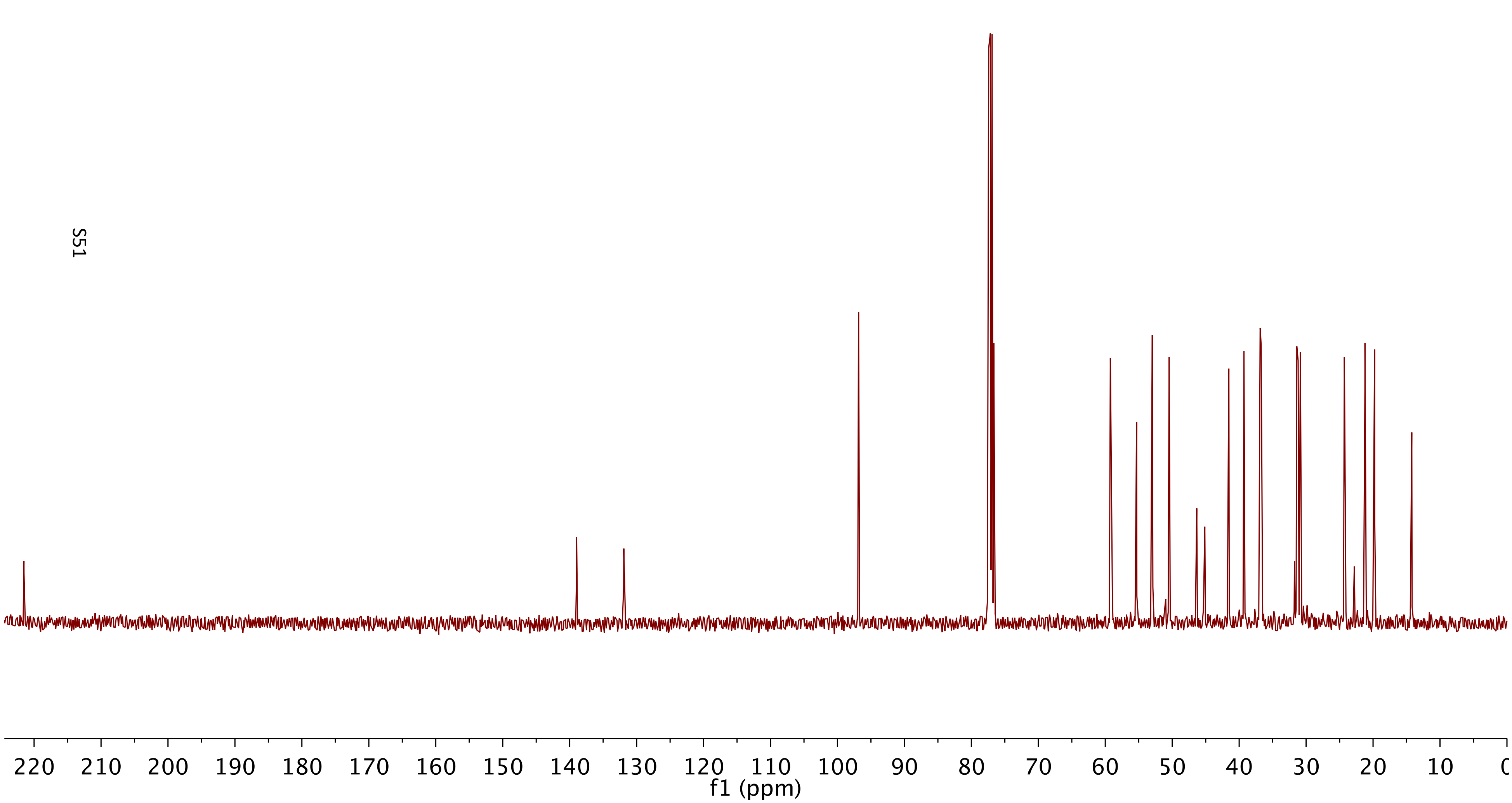

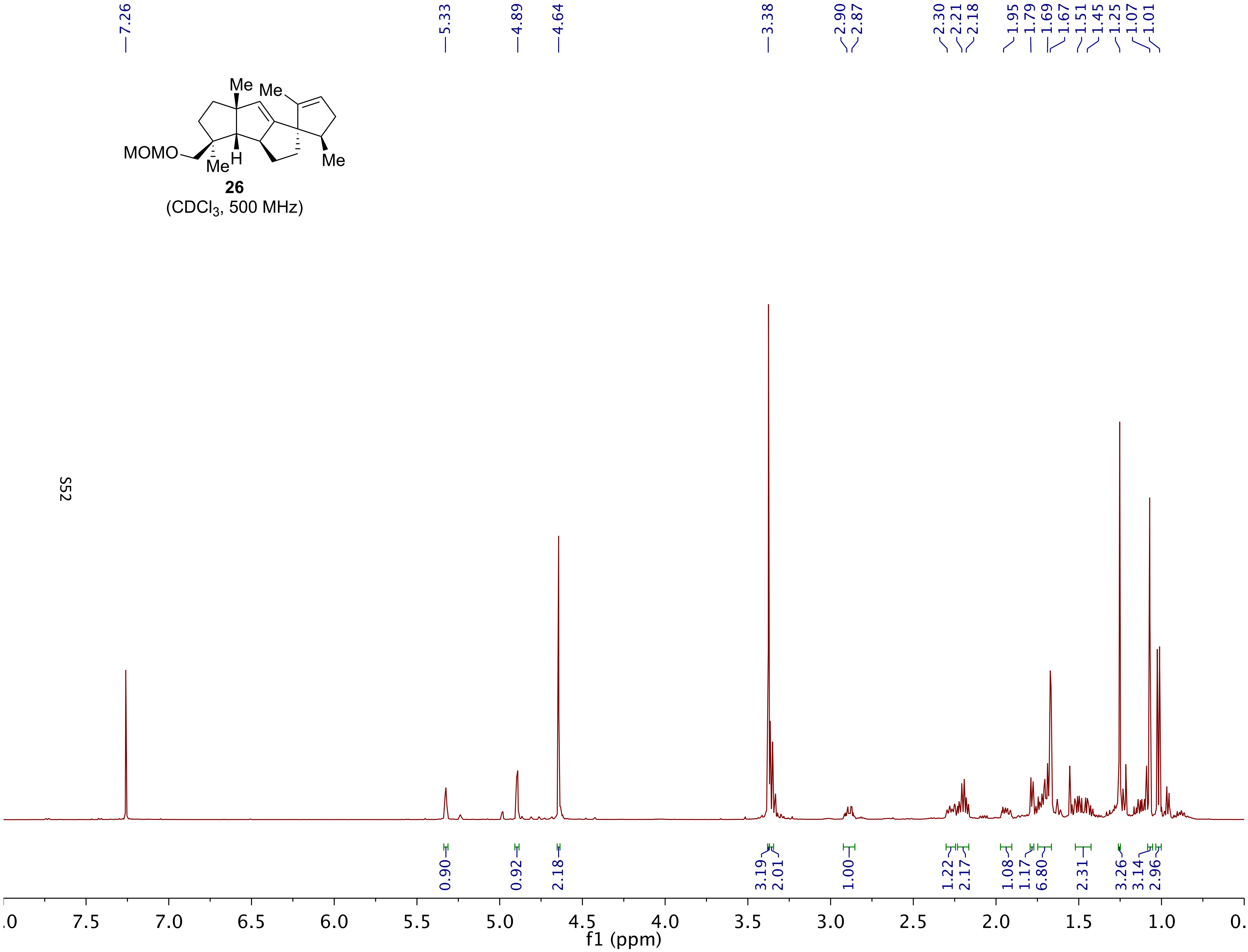
7.26
5.33
4.89
4.64
3.38
2.90
2.87
2.30
2.21
2.18
1.95
1.79
1.69
1.67
1.51
1.45
1.25
1.07
1.01
MOMO
Me
Me
Me
H
Me
26
(CDCl3, 500 MHz)
0.90
0.92
2.18
3.19
2.01
1.00
1.22
2.17
1.08
1.17
6.80
2.31
3.26
3.14
2.96
7.5
7.0
6.5
6.0
5.5
5.0
4.5
4.0
3.5
3.0
2.5
2.0
1.5
1.0
f1 (ppm)

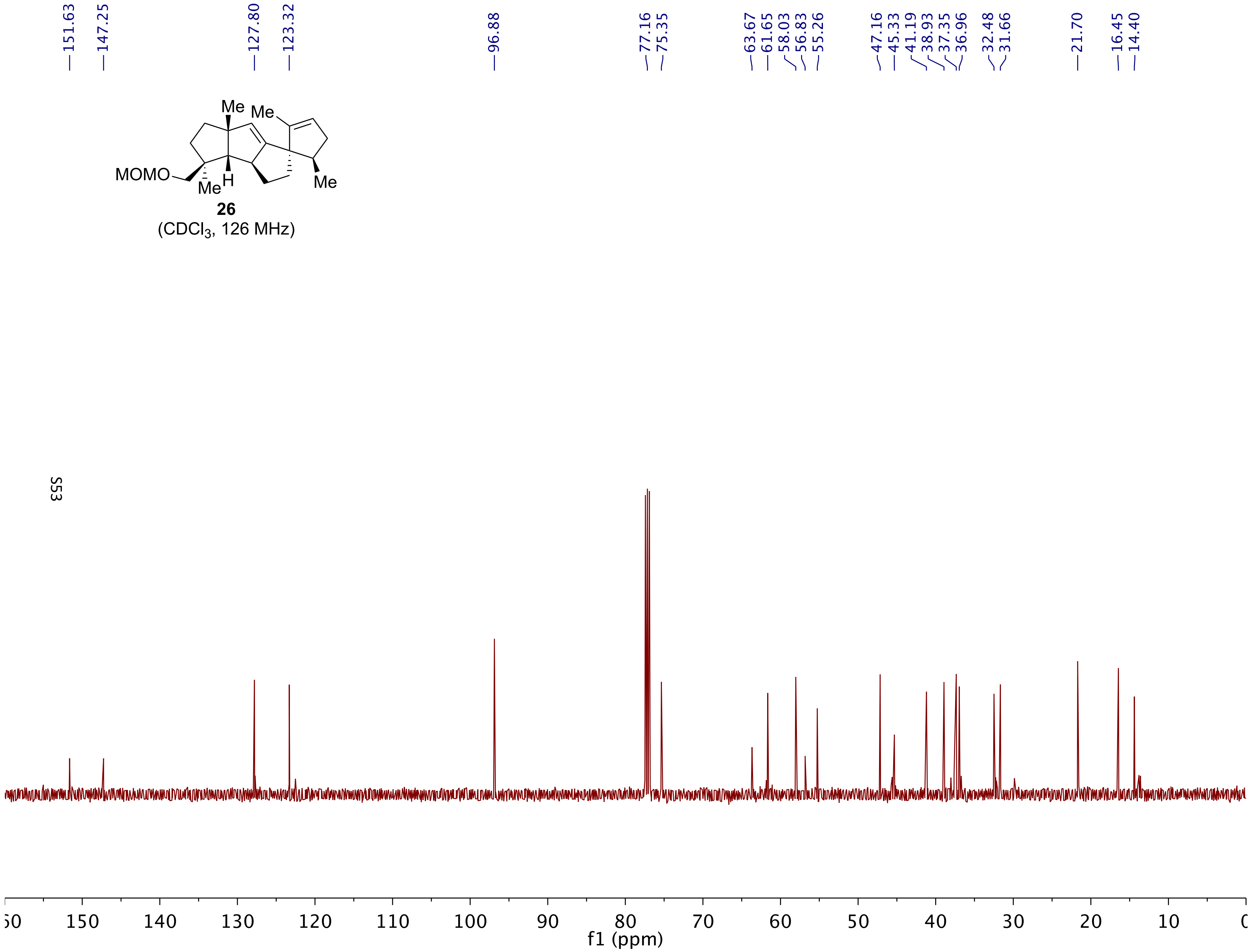
151.63
147.25
127.80
123.32
96.88
77.16
75.35
63.67
61.65
58.03
56.83
55.26
47.16
45.33
41.19
38.93
37.35
36.96
32.48
31.66
21.70
16.45
14.40
Me
Me
MOMO
Me
H
Me
26
(CDCl3, 126 MHz)
150
140
130
120
110
100
90
80
70
60
50
40
30
20
10
f1 (ppm)

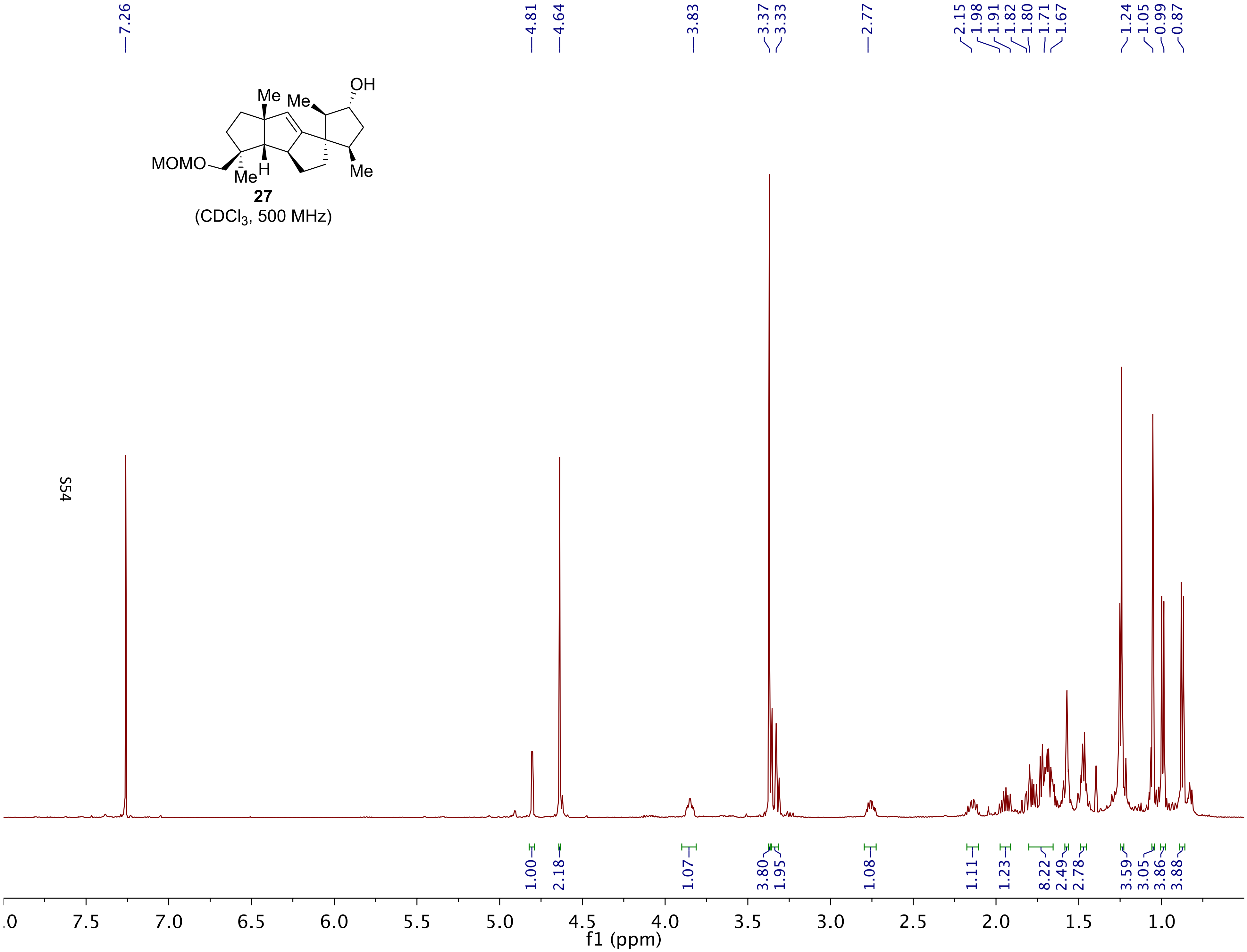

27

($CDCl_3$, 500 MHz)

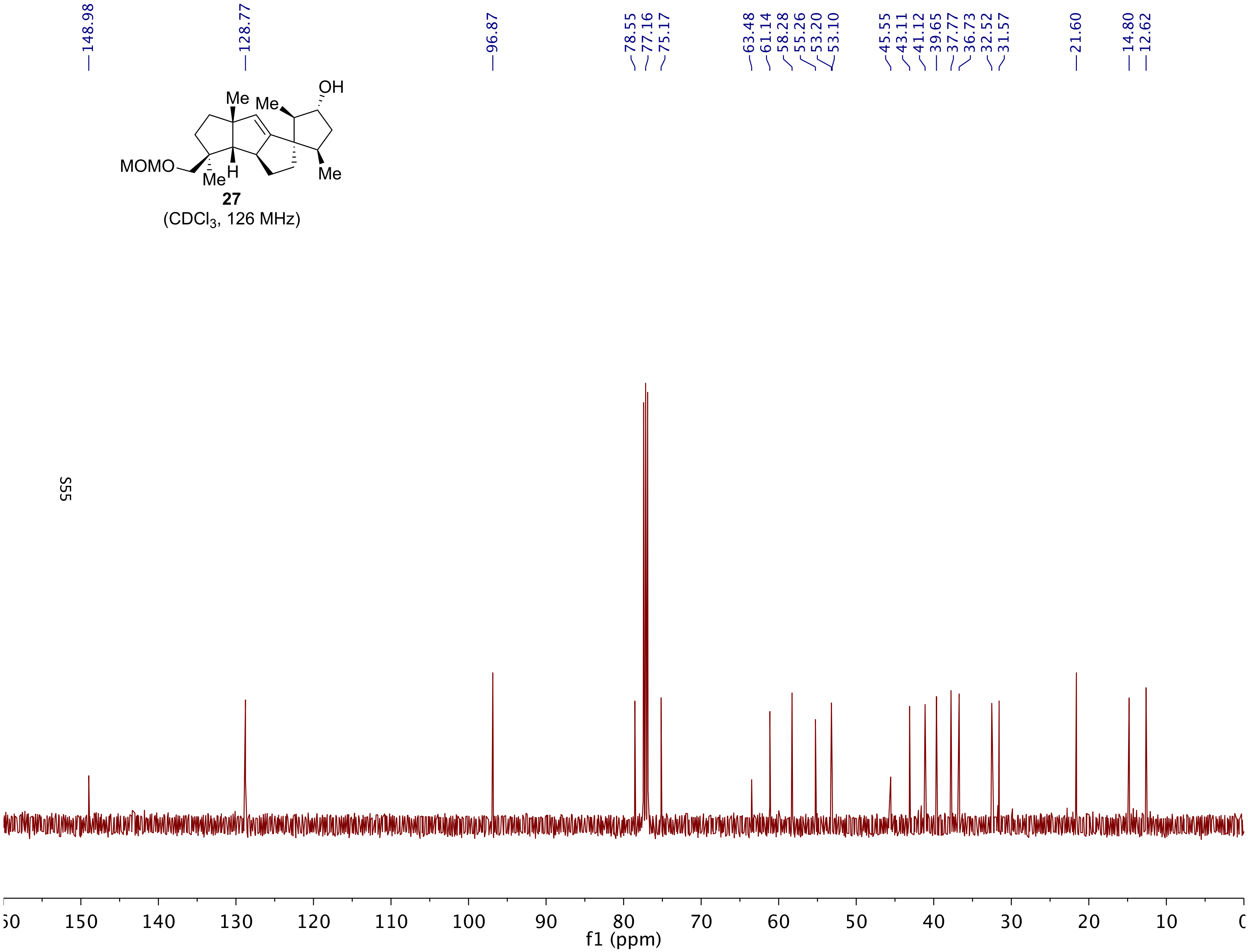
148.98
128.77
96.87
78.55
77.16
75.17
63.48
61.14
58.28
55.26
53.20
53.10
45.55
43.11
41.12
39.65
37.77
36.73
32.52
31.57
21.60
14.80
12.62
Me
Me
OH
MOMO
Me
H
Me
27
(CDCl3, 126 MHz)
f1 (ppm)

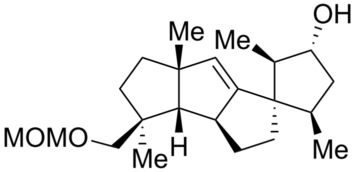

27
($CDCl_3$, 500 MHz)

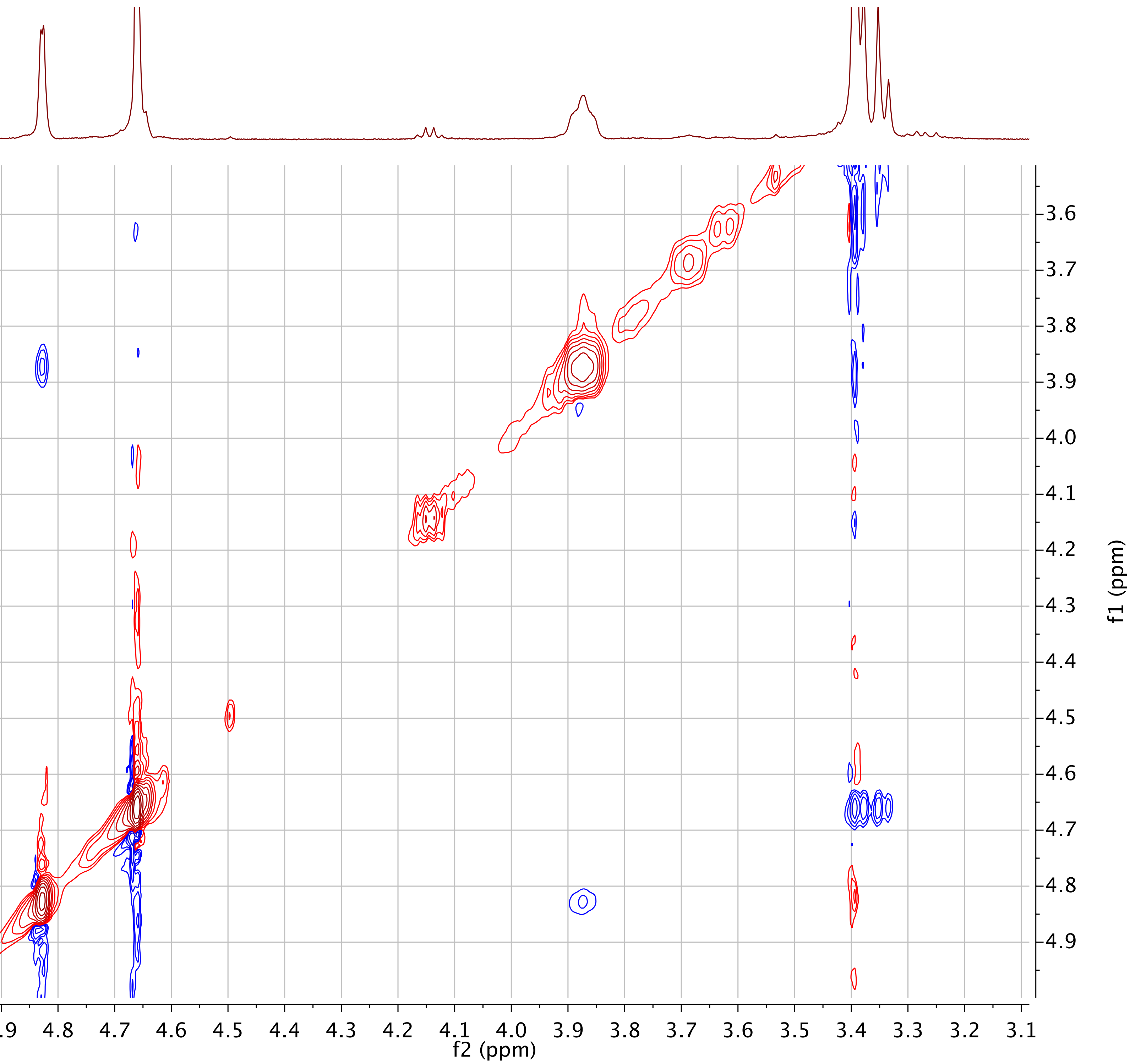

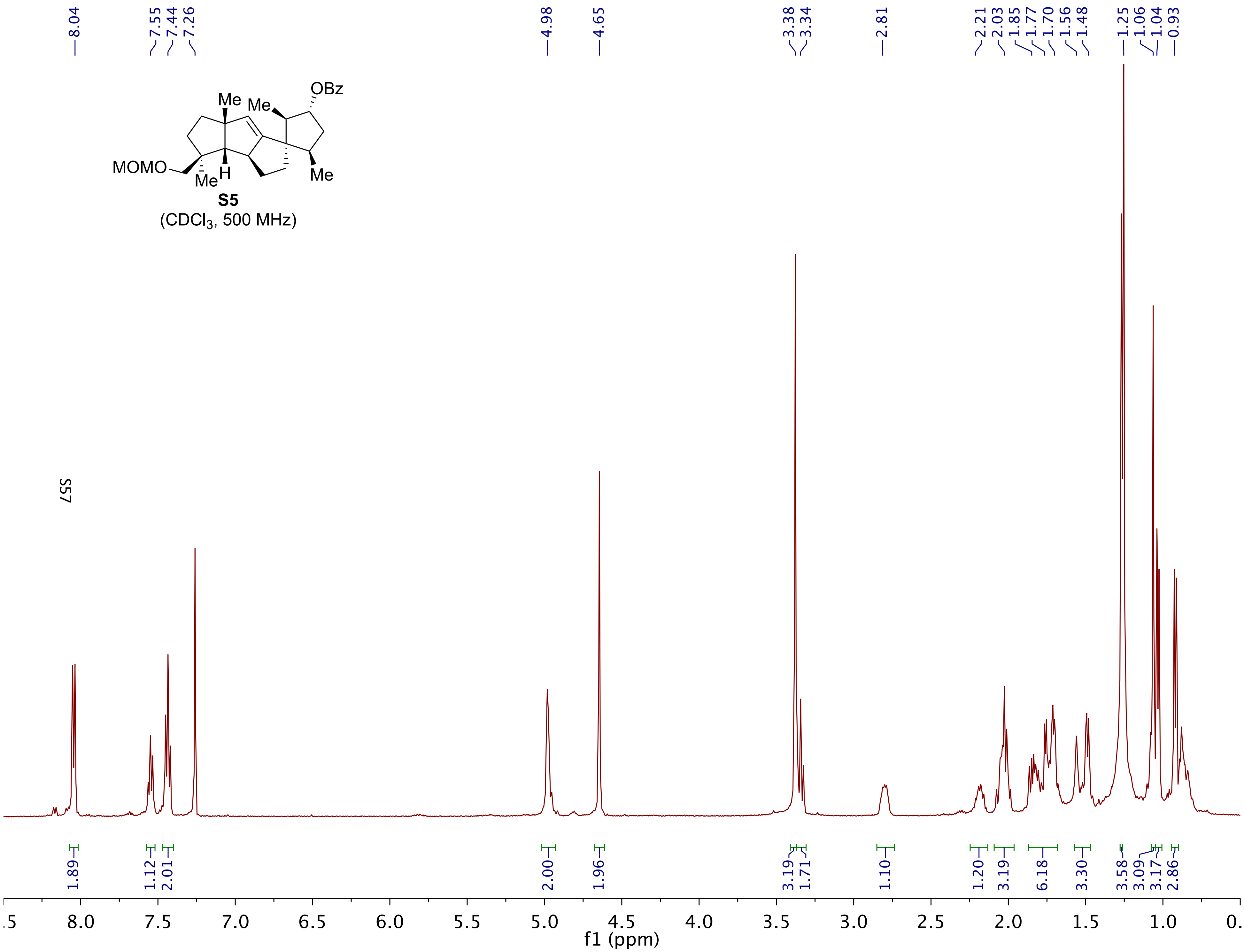

S5
($CDCl_3$, 500 MHz)

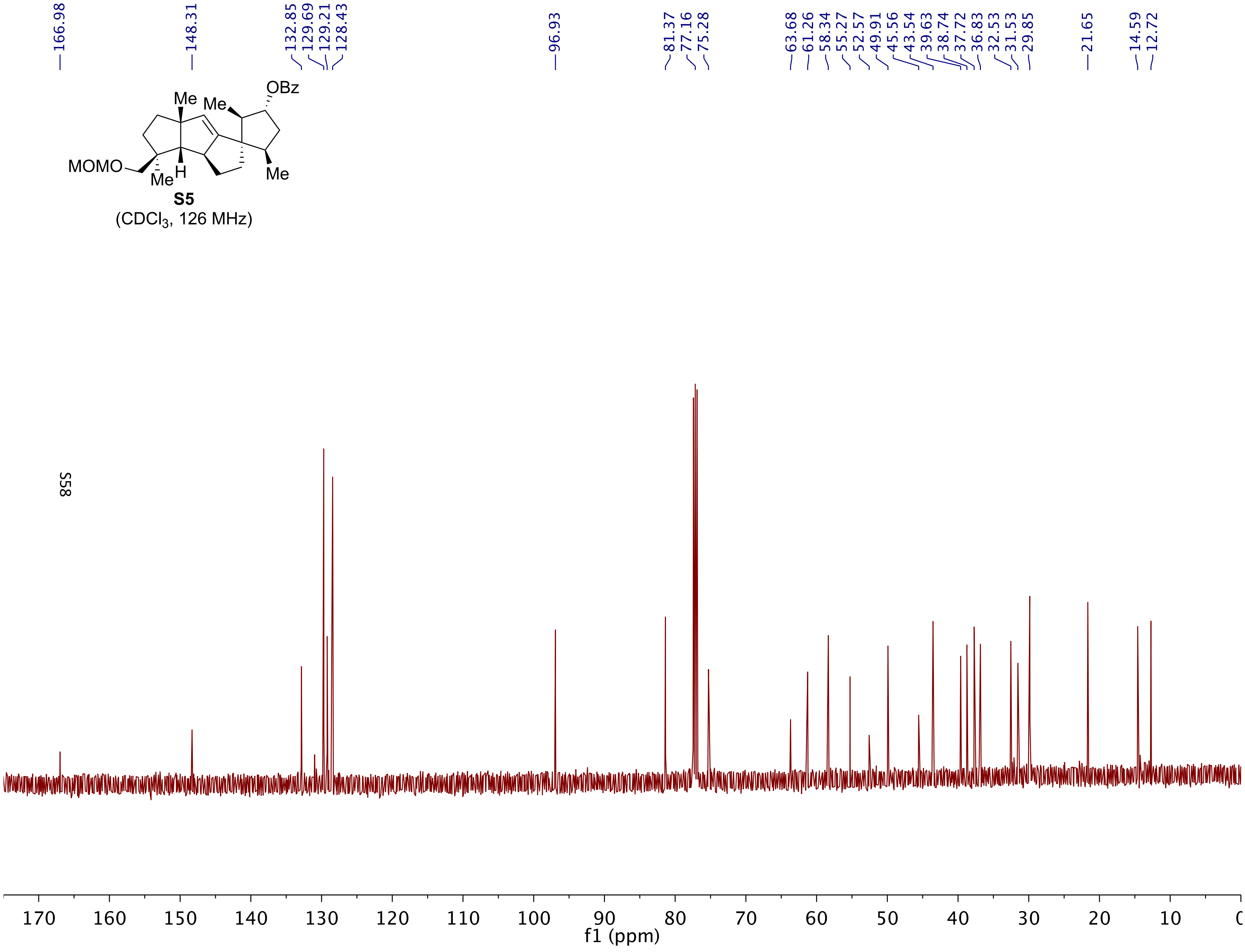

S5
($CDCl_3$, 126 MHz)

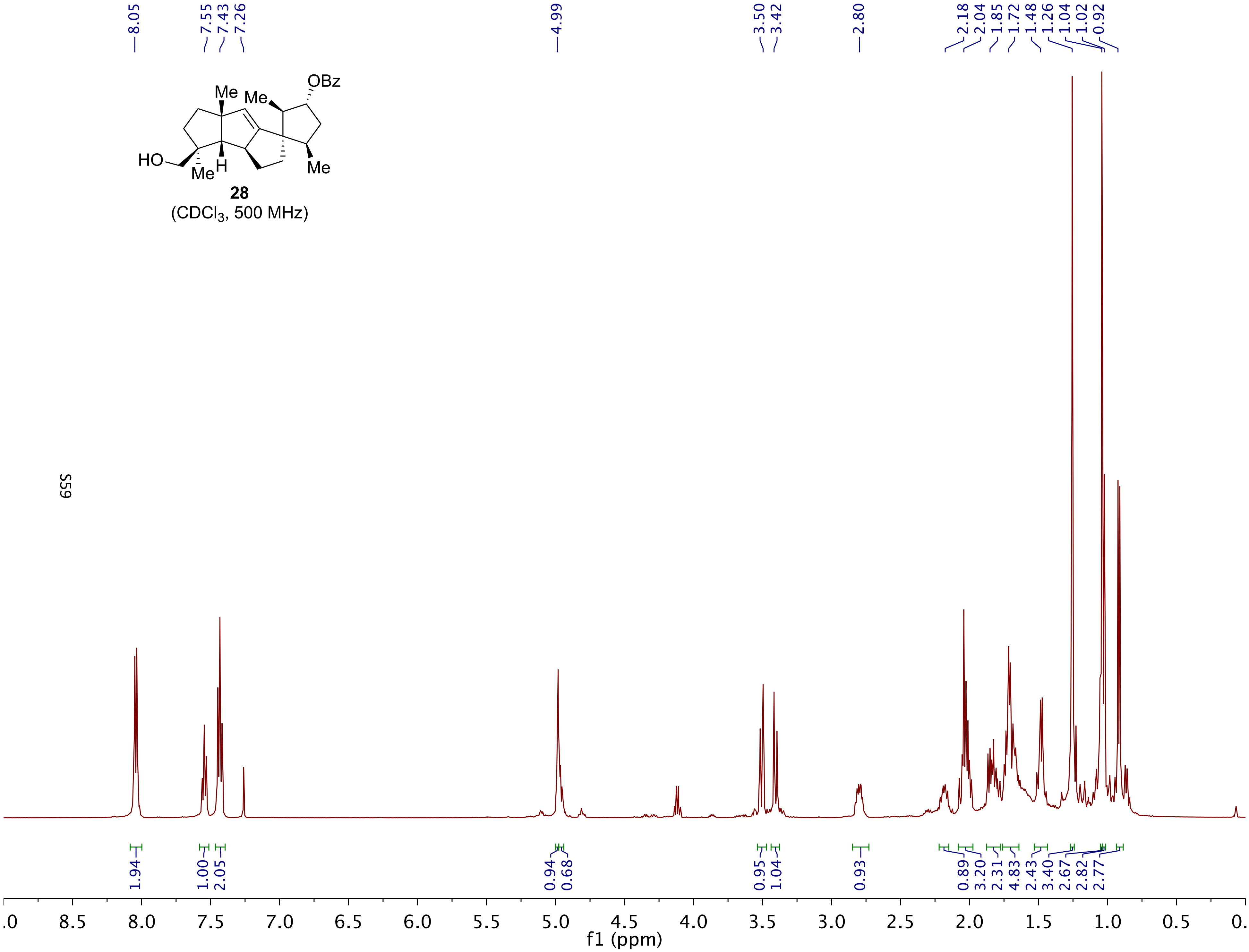

28
($CDCl_3$, 500 MHz)

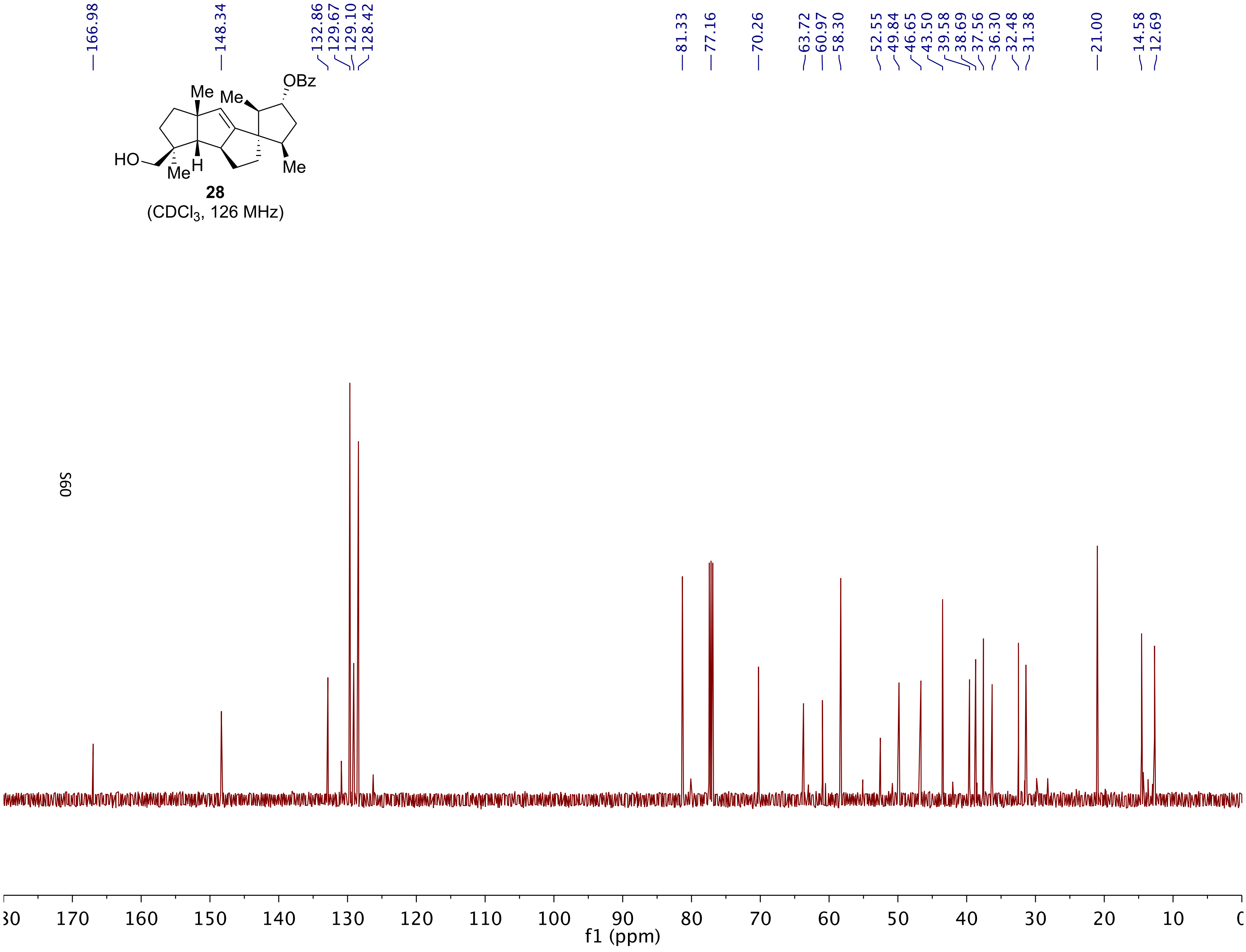
166.98
148.34
132.86
129.67
129.10
128.42
81.33
77.16
70.26
63.72
60.97
58.30
52.55
49.84
46.65
43.50
39.58
38.69
37.56
36.30
32.48
31.38
21.00
14.58
12.69
OBz
Me
Me
HO
Me
H
Me
28
(CDCl3, 126 MHz)
f1 (ppm)

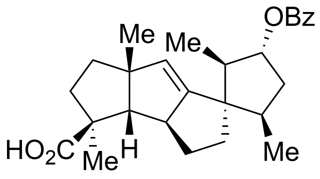

29
($CDCl_3$, 500 MHz)

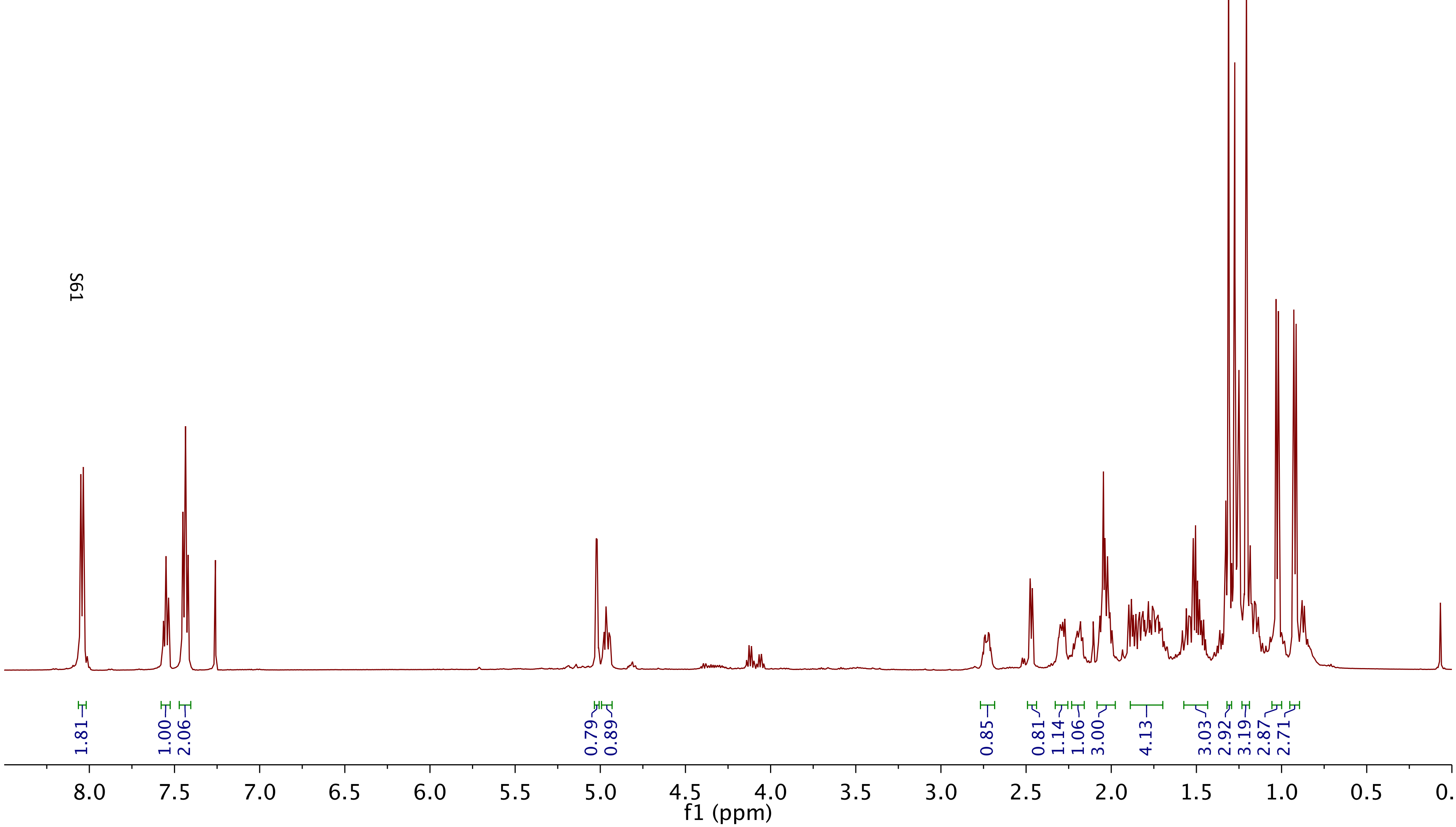

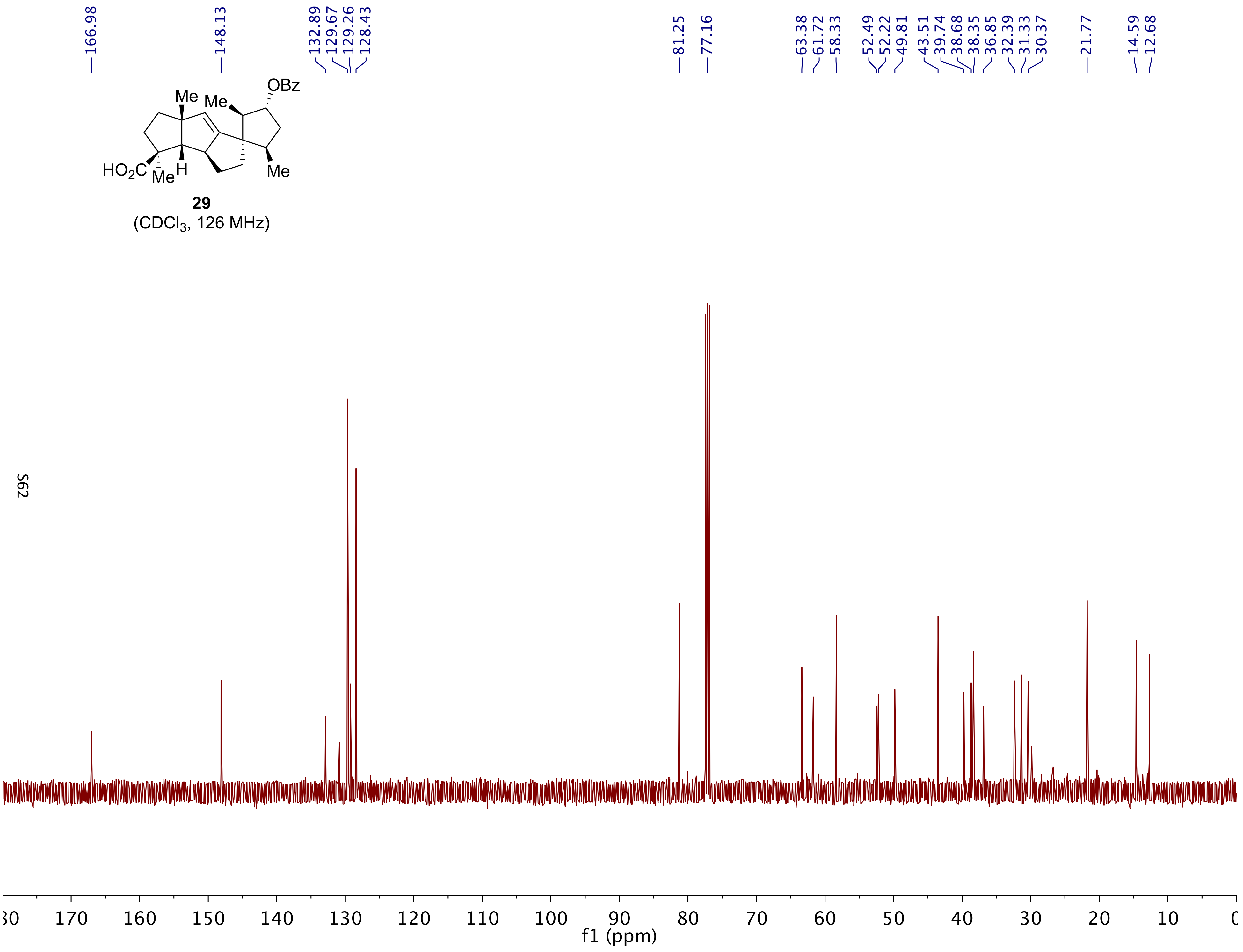

29
($CDCl_3$, 126 MHz)

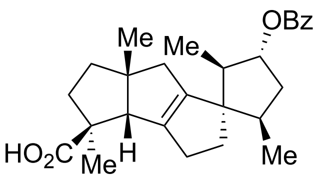

30
($CDCl_3$, 500 MHz)

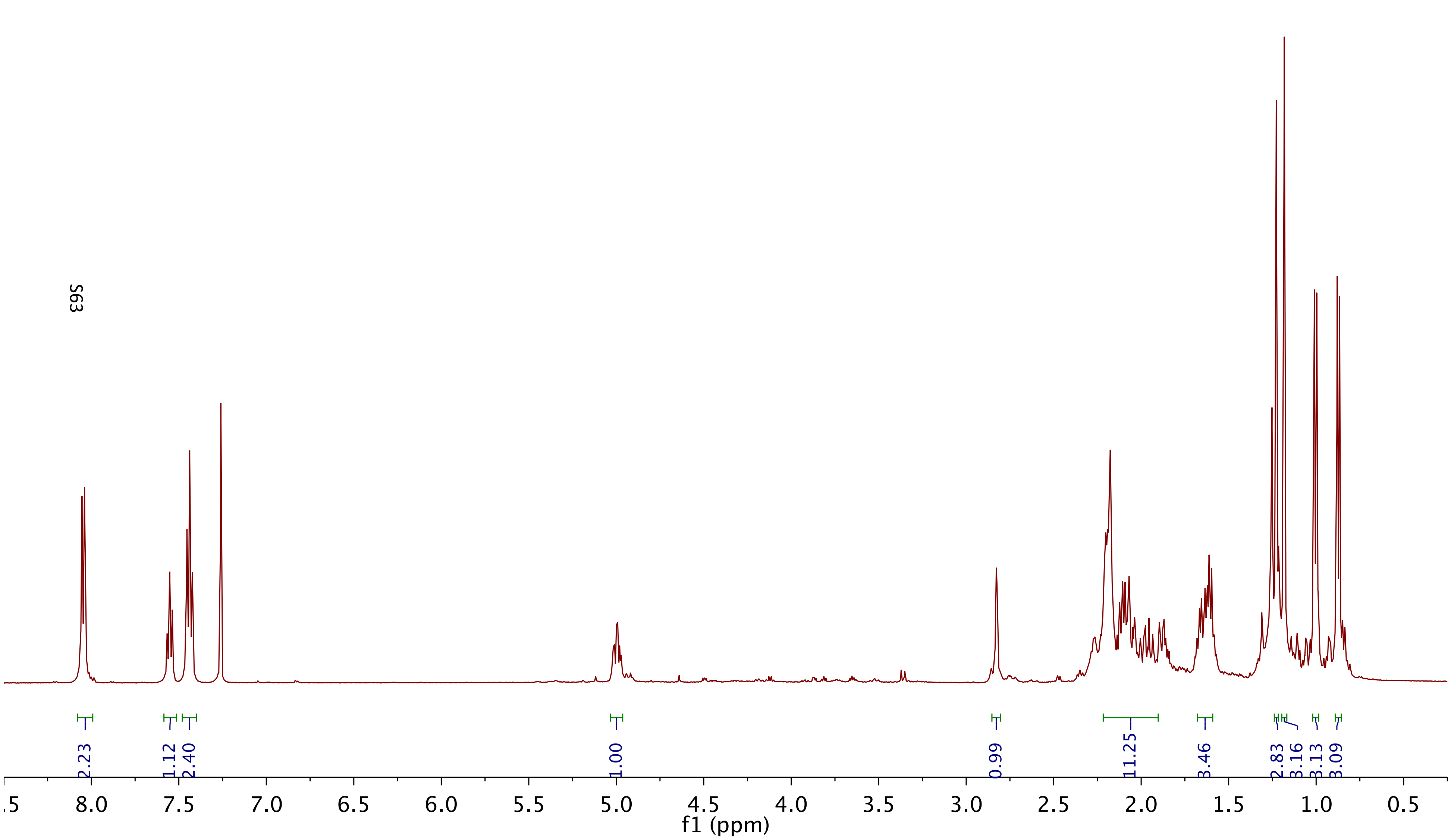

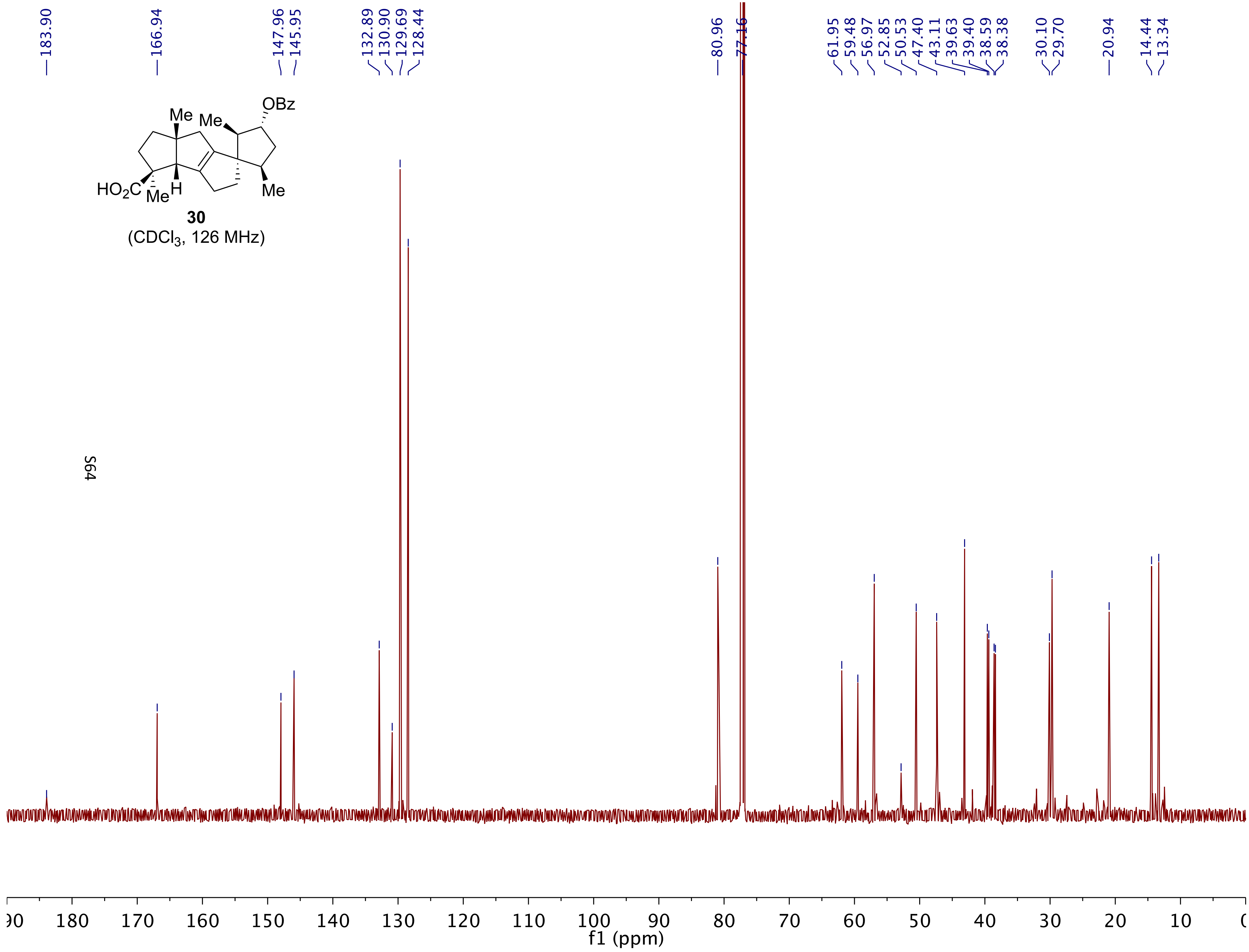

30
($CDCl_3$, 126 MHz)

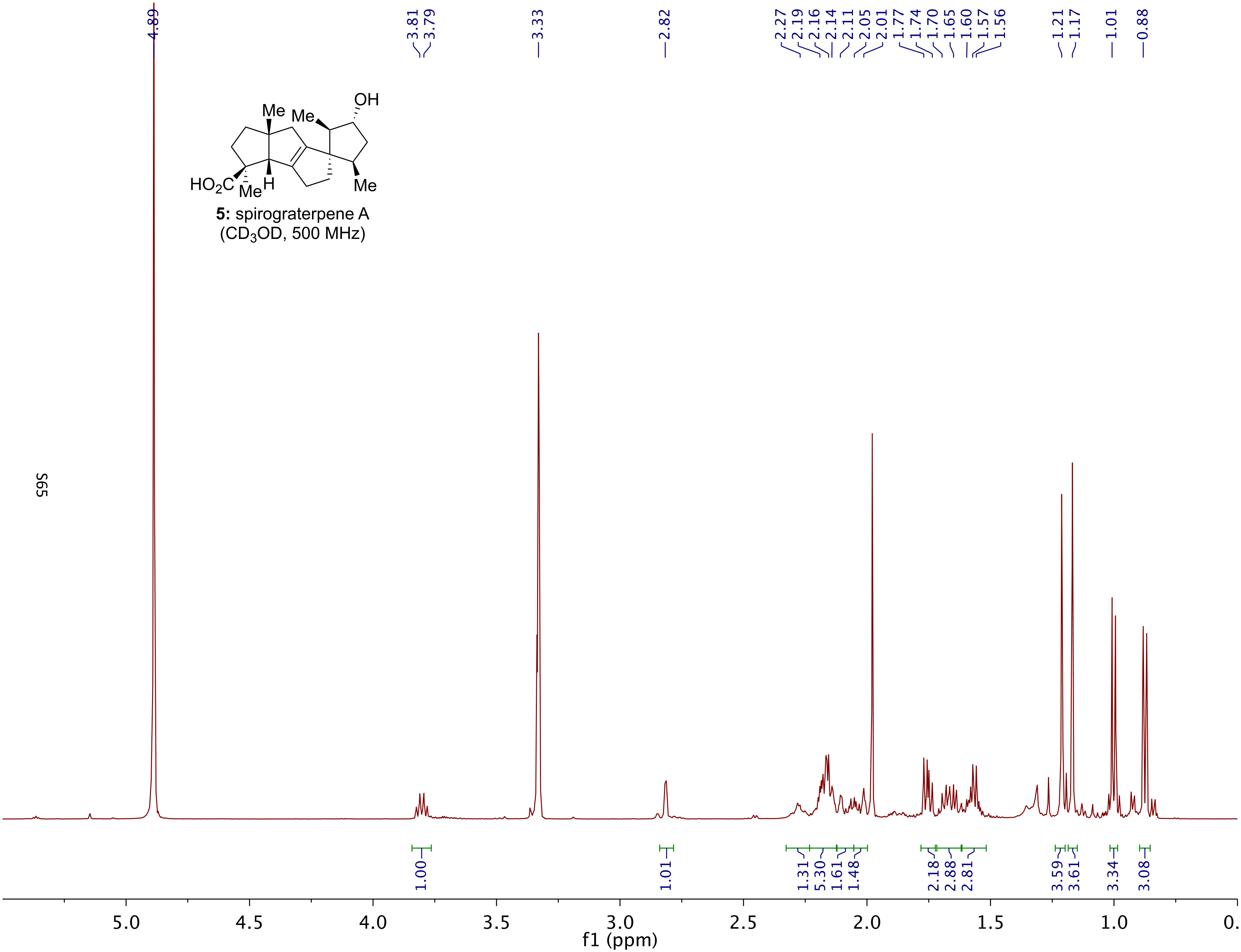

5: spirograterpene A
(CD_3OD, 500 MHz)

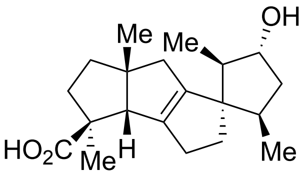

5: spirograterpene A
(CD_3OD, 126 MHz)

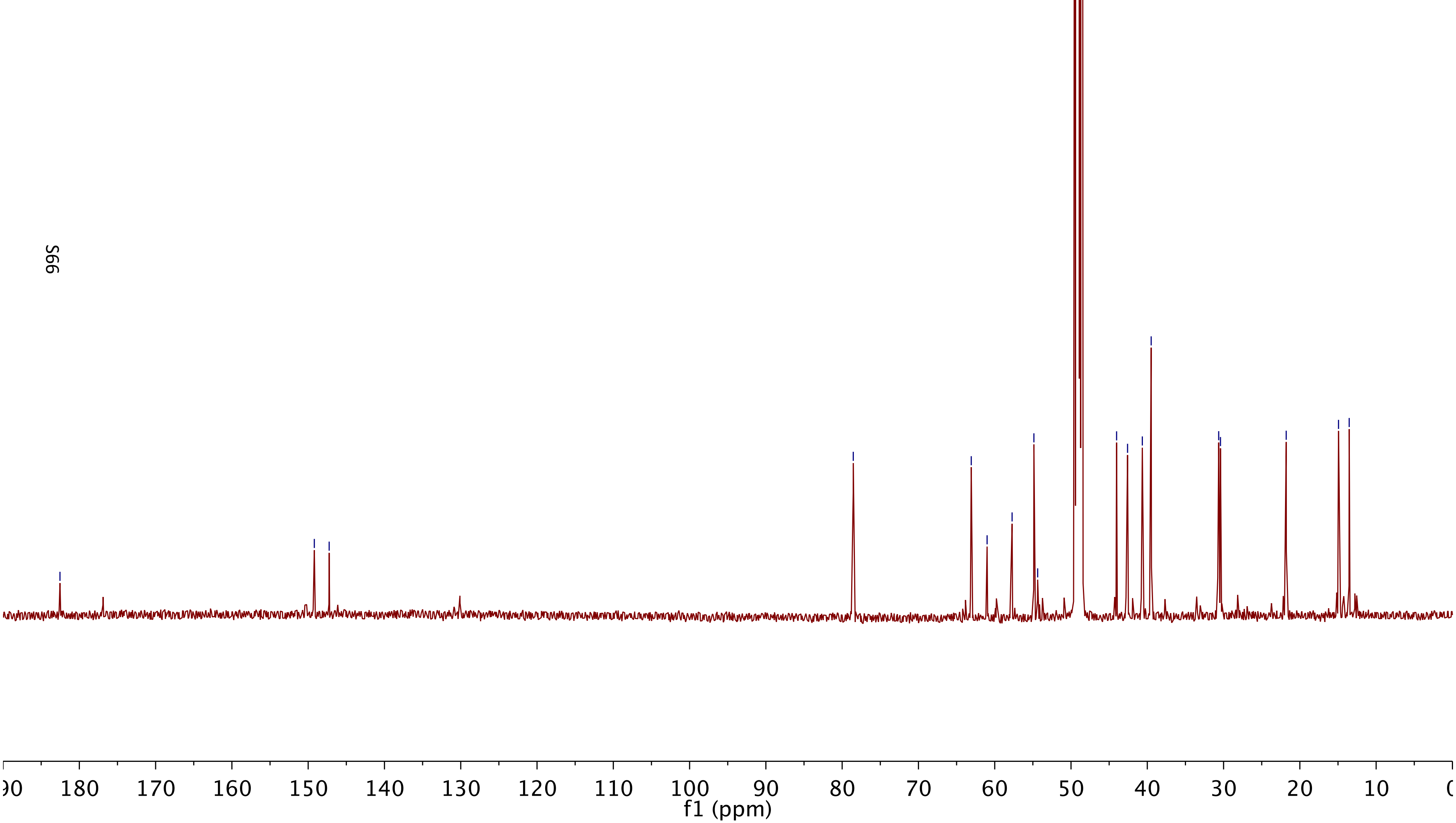

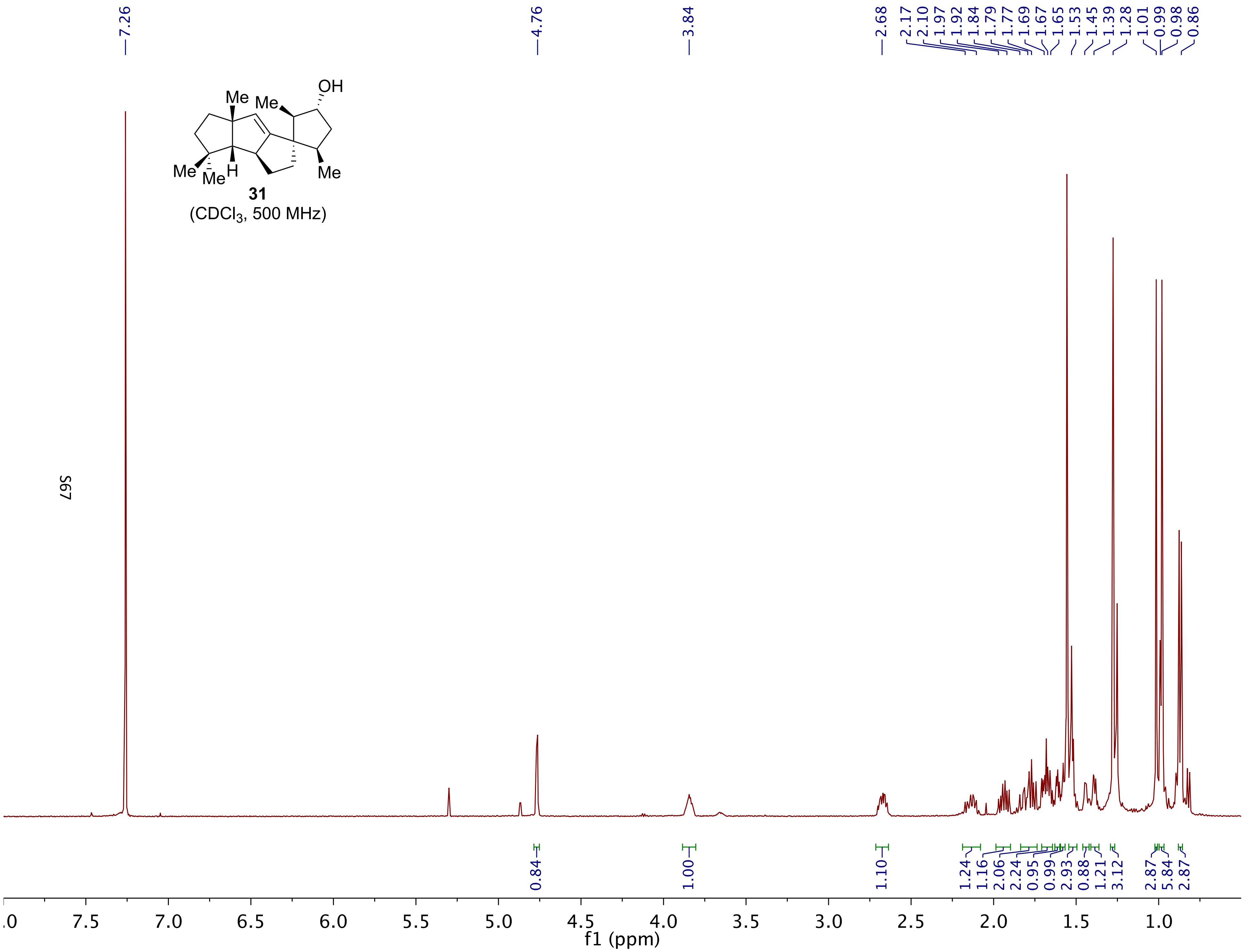
7.26
4.76
3.84
2.68
2.17
2.10
1.97
1.92
1.84
1.79
1.77
1.69
1.67
1.65
1.53
1.45
1.39
1.28
1.01
0.99
0.98
0.86
Me
Me
OH
Me
Me
Me
H
Me
31
(CDCl$_3$, 500 MHz)
0.84
1.00
1.10
1.24
1.16
2.06
2.24
0.95
0.99
2.93
0.88
1.21
3.12
2.87
5.84
2.87
f1 (ppm)

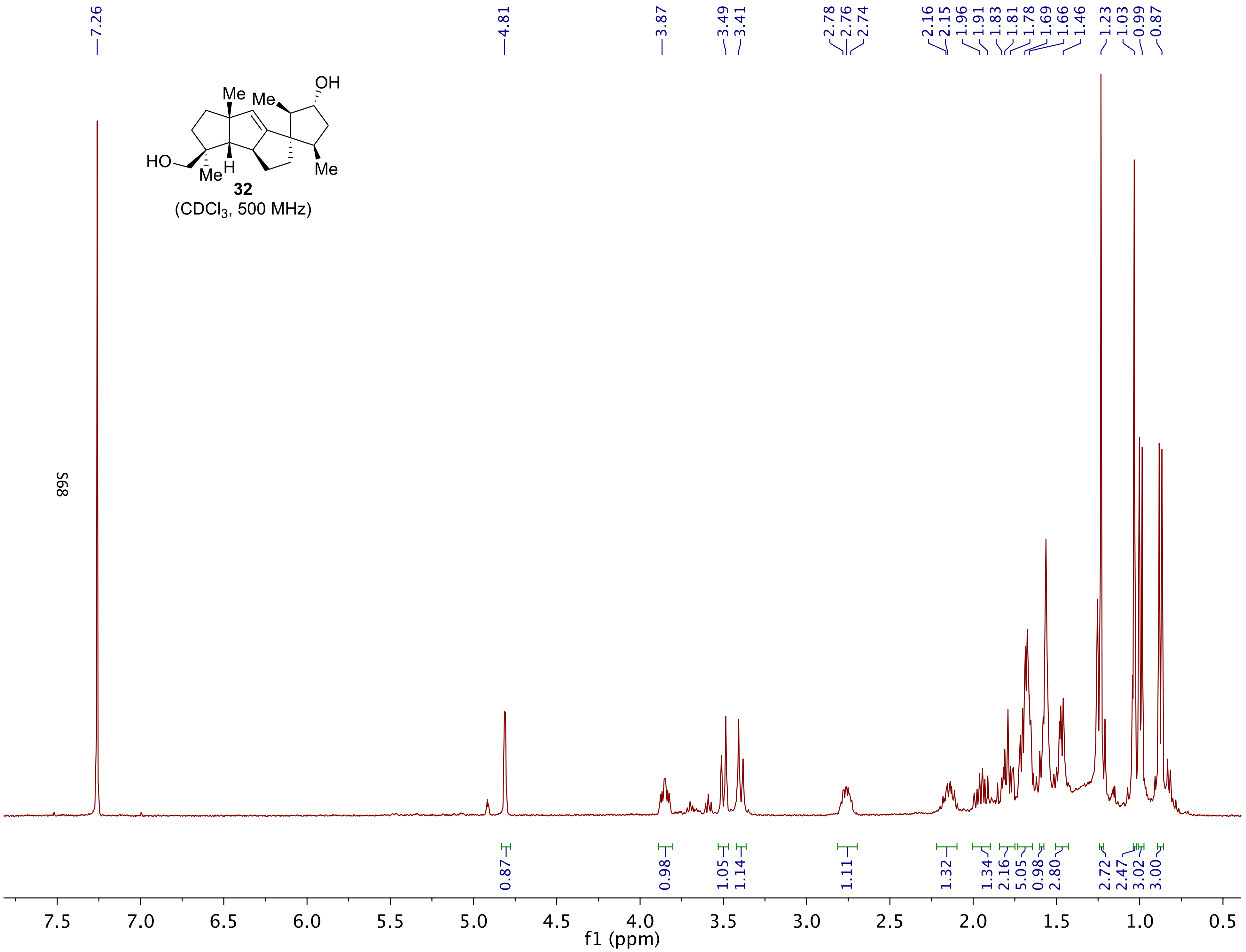

32

($CDCl_3$, 500 MHz)

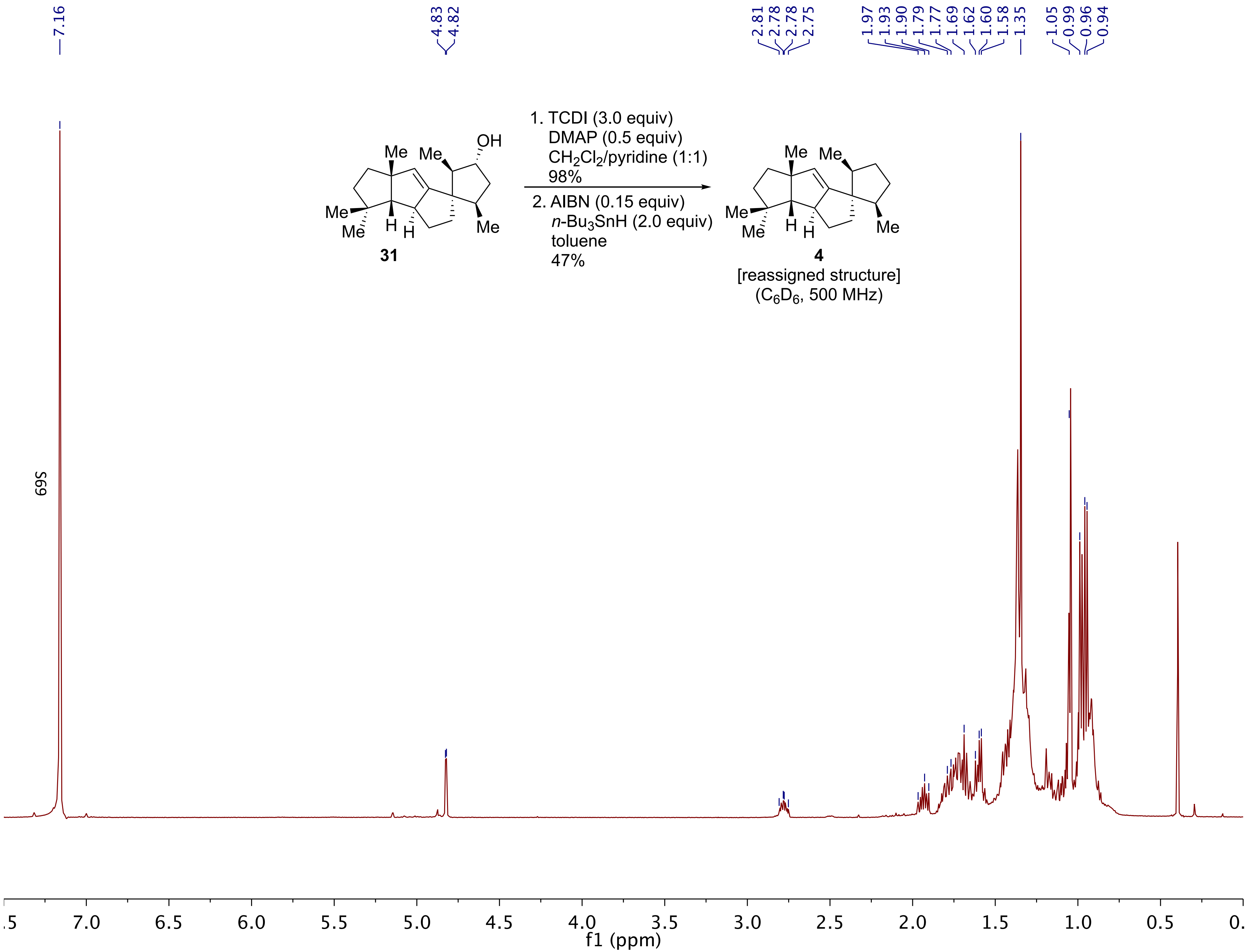
1. TCDI (3.0 equiv)
DMAP (0.5 equiv)
CH_2Cl_2/pyridine (1:1)
98%
2. AIBN (0.15 equiv)
n-Bu_3SnH (2.0 equiv)
toluene
47%
Me
OH
H
31
4
[reassigned structure]
(C_6D_6, 500 MHz)
7.16
4.83
4.82
2.81
2.78
2.78
2.75
1.97
1.93
1.90
1.79
1.77
1.69
1.62
1.60
1.58
1.35
1.05
0.99
0.96
0.94
f1 (ppm)

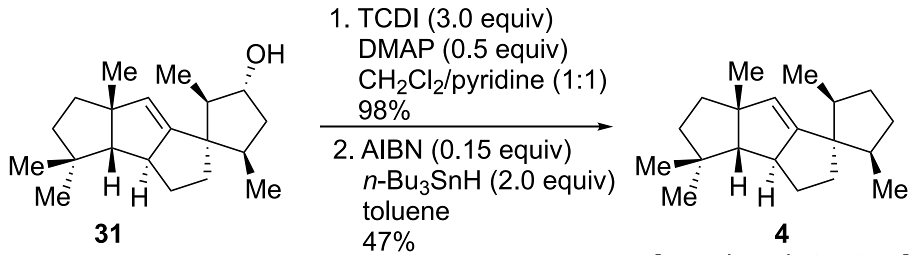

[reassigned structure]
(C_6D_6, 126 MHz)

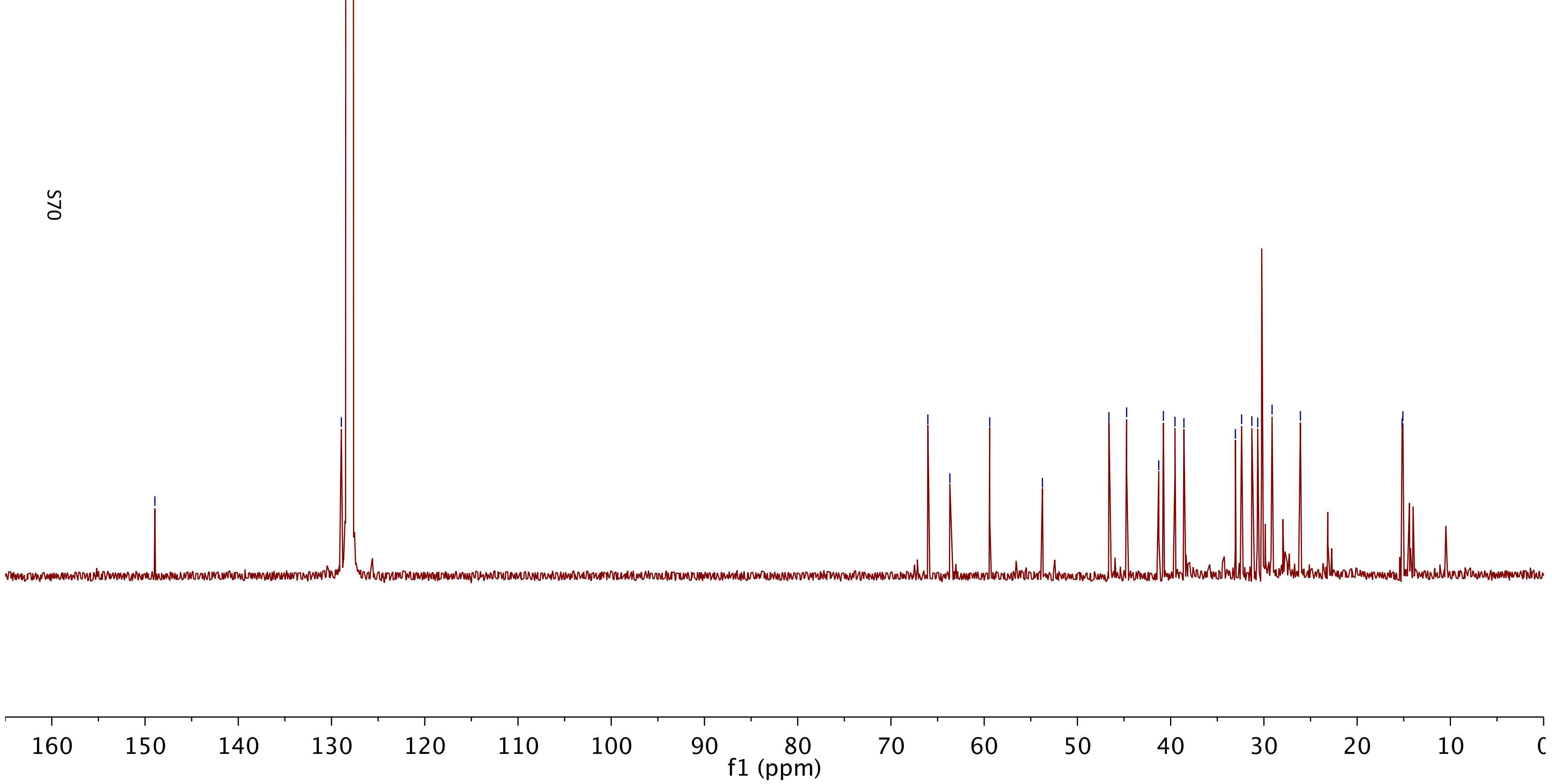

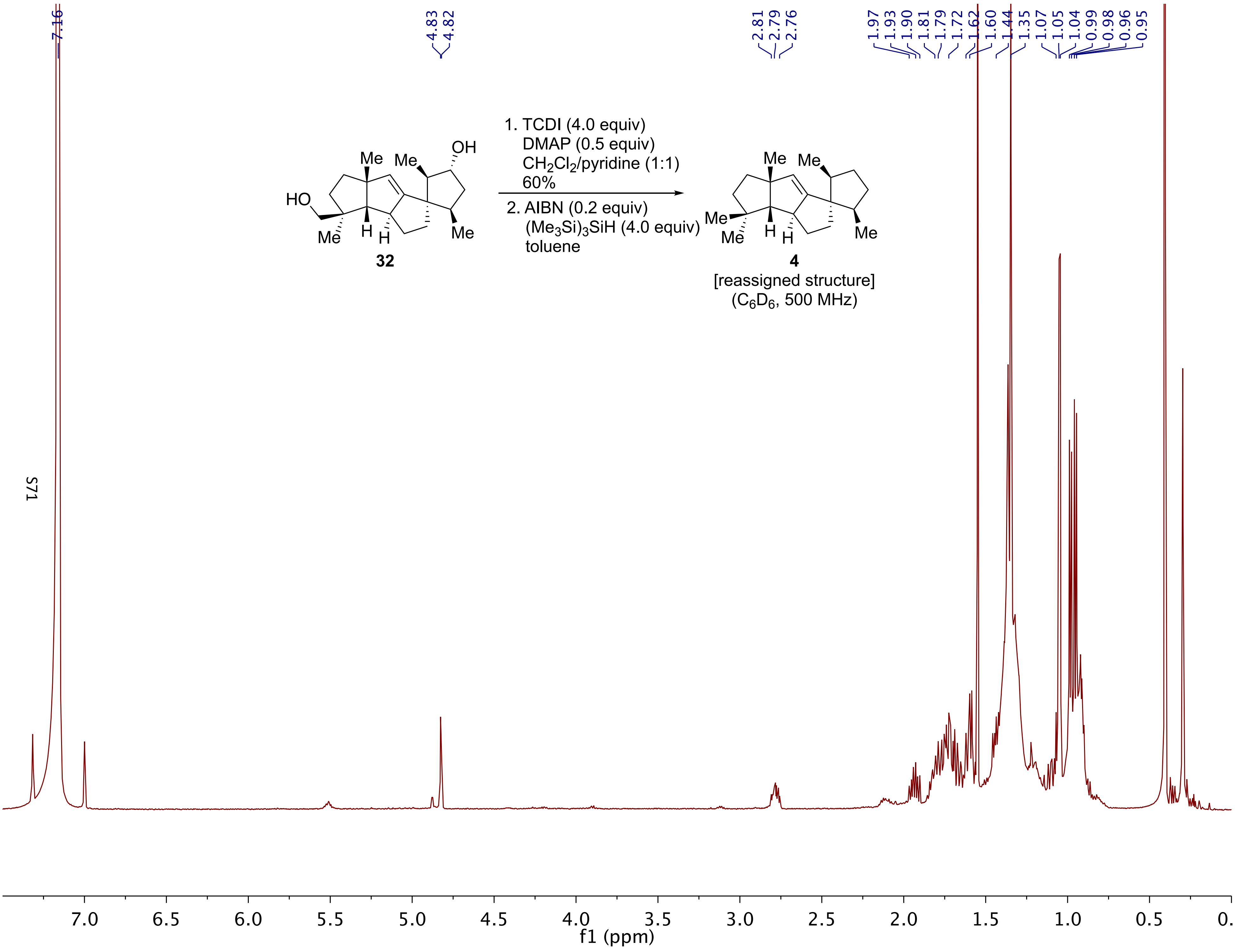
7.16
4.83
4.82
2.81
2.79
2.76
1.97
1.93
1.90
1.81
1.79
1.72
1.62
1.60
1.44
1.35
1.07
1.05
1.04
0.99
0.98
0.96
0.95
HO
Me
Me
Me
OH
H
H
Me
32
1. TCDI (4.0 equiv)
DMAP (0.5 equiv)
CH_2Cl_2/pyridine (1:1)
60%
2. AIBN (0.2 equiv)
$(Me_3Si)_3SiH$ (4.0 equiv)
toluene
4
[reassigned structure]
(C_6D_6, 500 MHz)
7.0
6.5
6.0
5.5
5.0
4.5
4.0
3.5
3.0
2.5
2.0
1.5
1.0
0.5
0.
f1 (ppm)

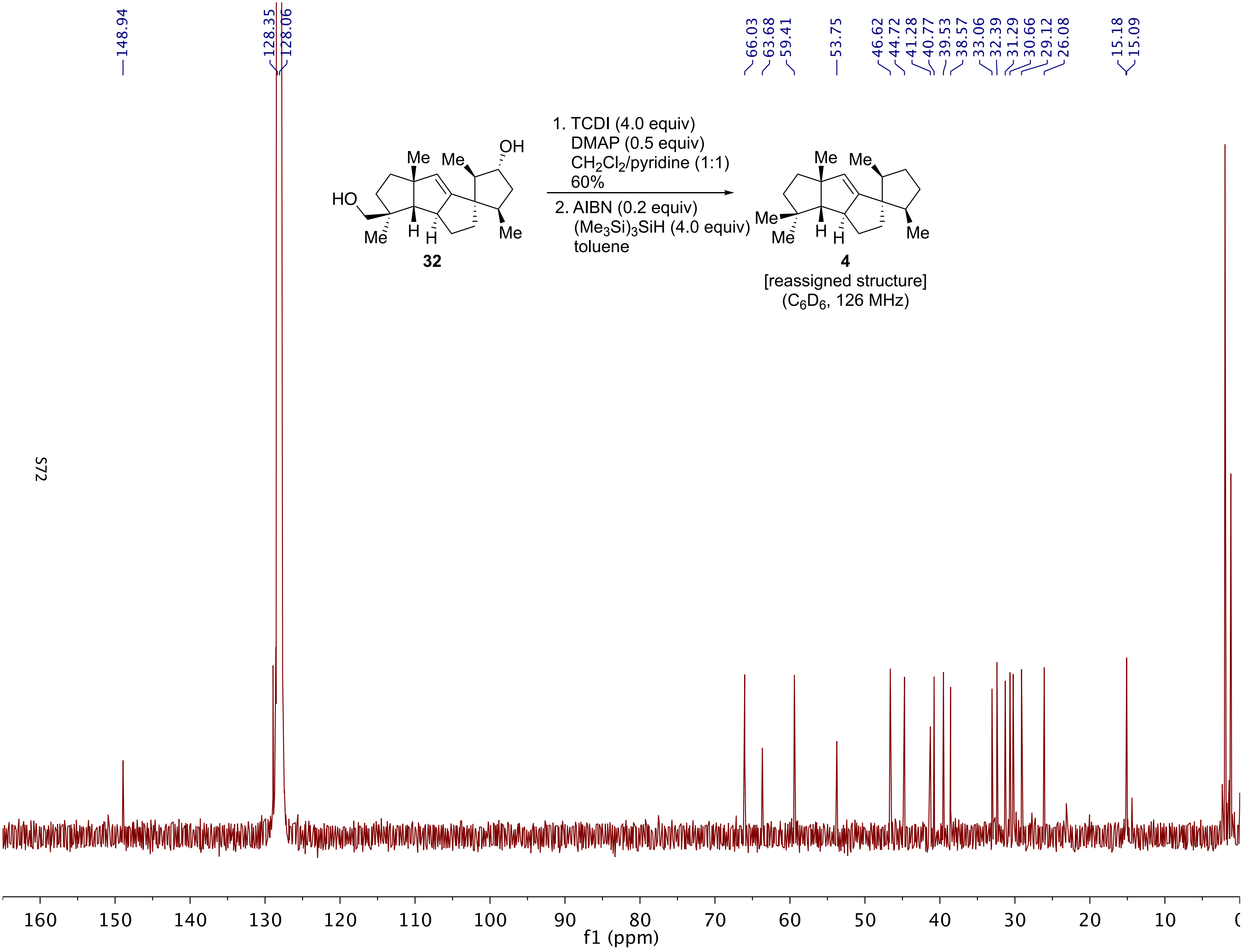
148.94
128.35
128.06
66.03
63.68
59.41
53.75
46.62
44.72
41.28
40.77
39.53
38.57
33.06
32.39
31.29
30.66
29.12
26.08
15.18
15.09
HO
Me
Me
Me
OH
H
H
Me
32
1. TCDI (4.0 equiv)
DMAP (0.5 equiv)
CH_2Cl_2/pyridine (1:1)
60%
2. AIBN (0.2 equiv)
$(Me_3Si)_3SiH$ (4.0 equiv)
toluene
Me
Me
Me
Me
H
H
Me
4
[reassigned structure]
(C_6D_6, 126 MHz)
160
150
140
130
120
110
100
90
80
70
60
50
40
30
20
10
f1 (ppm)

H. HPLC Traces

Racemic **S7** (Chiralpak AD-H, Hexanes/*i*PrOH 99:1, 240 nm)

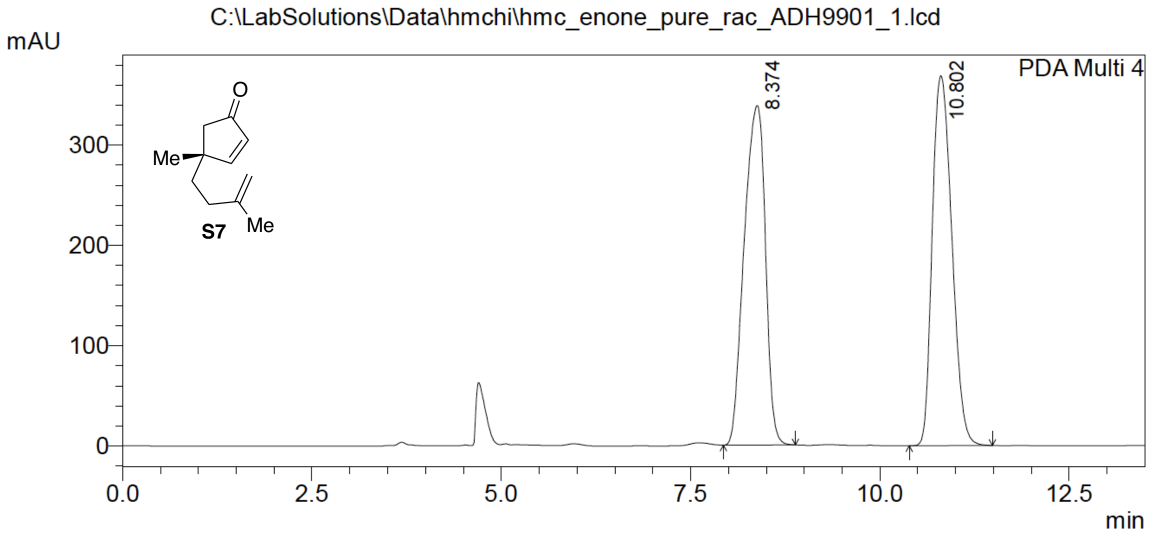

1 PDA Multi 4/240nm,4nm

Peak Table

PDA Ch4 240nm

Peak#	Ret. Time	Area	Height	Area%
1	8.374	6592268	338557	49.776
2	10.802	6651515	369269	50.224
Total		13243783	707825	100.000

Enantioenriched **S7** (Chiralpak AD-H, Hexanes/*i*PrOH 99:1, 240 nm)

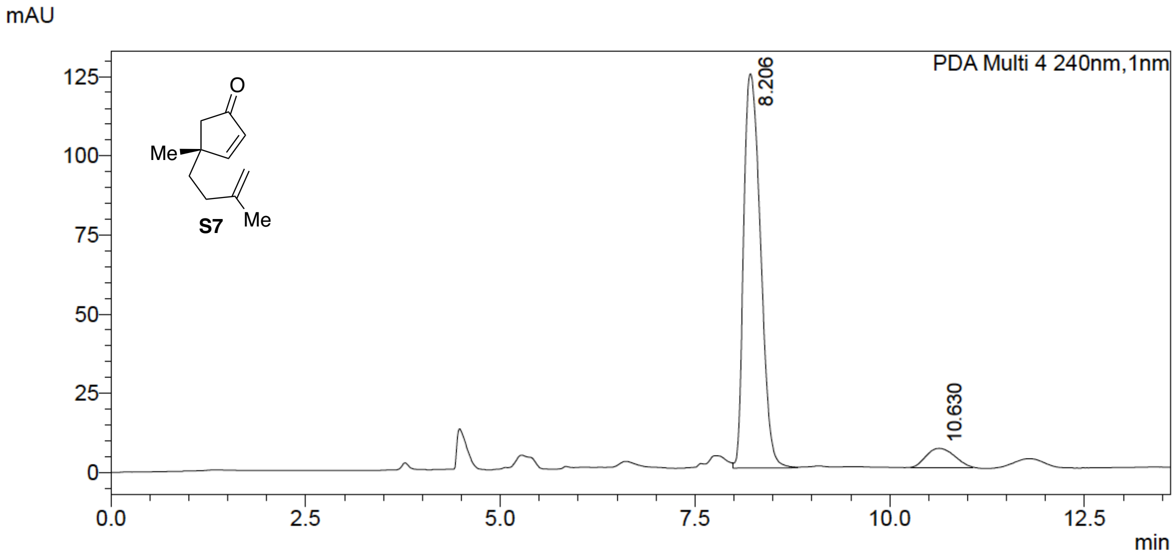

<Peak Table>

PDA Ch4 240nm

Peak#	Ret. Time	Area	Height	Area%
1	8.206	1875709	124550	92.648
2	10.630	148846	5965	7.352
Total		2024556	130515	100.000
